# Phylogenetic affiliation of endophytic actinobacteria associated with red gum tree grown in salinity area and their plant growth promoting properties and suppression of phytopathogens, and genome data mining of selected strains

**DOI:** 10.3389/fpls.2025.1610327

**Published:** 2025-11-26

**Authors:** Onuma Kaewkla, Kawintip Kiakhunthod, Sumalee Chookhampaeng, Busayarat Klinjantasorm, Piriya Klankeo, Winya Dungkaew

**Affiliations:** 1 Department of Biology, Faculty of Science, Mahasarakham University, Kantharawichai, Maha Sarakham, Thailand; 2 The Northeastern Soil Salinity Research Unit, Faculty of Science, Mahasarakham University, Kantharawichai, Maha Sarakham, Thailand; 3 Faculty of Science, Omics Science and Bioinformatics Center, Chulalongkorn University, Bangkok, Thailand

**Keywords:** endophyte, *Eucalyptus camaldulensis*, saline soil, plant growth promoting, *Streptomyces*, genome insight

## Abstract

Eucalyptus is an economic plant of Thailand that can tolerate salt and drought stress. This work aims to report on the study of biodiversity among endophytic actinobacteria isolated from *Eucalyptus camaldulensis* grown in saline soil, as well as their properties for promoting plant growth and inhibiting fungal pathogens *in vitro*. Root, twig, and leaf samples of five plants, *E. camaldulensis* grown in Kalasin Province, Thailand, were collected. The soil samples of each plant were collected, and soil salinity was evaluated by the electrical conductivity of a saturated soil extract (ECe). It was found that the ECe of the soil was between 4.5 and 12 dS/m, and the pH of the soil ranged between 3.9 and 5.2. Based on their morphology and 16S rRNA gene sequences, the majority of the isolates (552, 96.7%) were identified as the genus *Streptomyces*. The remaining isolates (19, 3.3%), which included ten genera: *Cellulosimicrobium* (3), *Kocuria* (3), *Brevibacterium* (2), *Micrococcus* (2), *Microbacterium* (2), *Peterkaempfera* (2), *Tsukamurella* (2), *Brachybacterium* (1), *Curtobacterium* (1), and *Gordonia* (1). Two hundred and seventy-three isolates were tested for antifungal activity against two eucalyptus pathogens, *Pseudoplagiostroma eucalypti* LS6 and *Cladosporium* sp. LB1. Most isolates showed antifungal activity against *P. eucalypti* LS6. The plant growth-promoting (PGP) study of 154 selected strains showed that 154 (100%), 18 (11.7%), 14 (9.1%), and 7 (4.5%) isolates could produce indole-3-acetic acid (IAA), 1-aminocyclopropane (ACC) deaminase, solubilize phosphate, and fix nitrogen *in vitro*, respectively. Identification of non-actinobacteria isolates based on 16S rRNA gene sequence analysis indicated that 10 genera were obtained: *Aureimonas, Bacillus, Chryseobacterium, Deinococcus, Massilia, Methylobacterium, Pseudomonas, Serratia, Staphylococcus*, and *Stenotrophomonas*. One selected *Streptomyces* strain, EWL5.1, was selected for a seed germination test in salinity stress and PGP *in planta*. The result indicated that this strain could support the seedling length vigor index (SLVI) of eucalyptus seedlings in salinity conditions and significantly increase the fresh weight of eucalyptus seedlings *in planta*. Four representative *Streptomyces* strains and one strain of *Micrococcus* were sequenced for their genomes. The result indicated that these four *Streptomyces* strains comprise various biosynthesis gene clusters (BGCs) of antibiotic production. Genome data mining also reveals that all strains contain genes encoding PGP properties. These potential strains can be applied to be used as PGPB to support eucalyptus growth in the future.

## Introduction

Endophytes are microorganisms living in the intercellular spaces of plant tissues without causing any disease symptoms. Some of these endophytes may benefit their hosts by producing plant growth-promoting (PGP) agents and inhibiting plant pathogens. Endophytes can be found in plants ranging from woody tree species to herbaceous crops. Actinomycetes, also known as Actinobacteria, are Gram-staining positives that comprise the Phylum Actinomycetota. These are spore-forming filamentous bacteria whose spores can survive in dry and stressed environments (Singh and Dubey, 2018). Actinobacteria are a major group that produce known antibiotics (more than 70%) to inhibit bacteria and fungi, including PGP properties. Actinobacteria produce phytohormones like auxin, cytokinin, and gibberellin; ACC deaminase to get rid of ethylene gas; osmoprotectants like proline, glycine-betaine, and polyamine; exopolysaccharides (EPS); and antioxidative enzymes to lower oxidative stress. These properties make it possible for plants to grow in salty conditions (Atouei et al., 2019; Feikema and Baker, 2011). Soil salinity distribution in wide areas of Thailand is a major problem for land use in agricultural areas. The majority area of saline soil is the Northeast, especially Kalasin and Maha Sarakham provinces. The high soil salinity affects the growth of any plants and leads to low productivity yield. Gum tree, or *Eucalyptus*, is an economic plant of Thailand. *Eucalyptus camaldulensis*, a widely distributed species in Thailand, produces wood pulp for the paper industry. There are many applications of eucalyptus, including paper pulp, cooking charcoal, crutch, timber, furniture, fly wood, chopped wood, and industrial fuel. *E. camaldulensis* can grow quickly and has the ability to tolerate drought and salinity (Marcar et al., 2002). Eucalyptus tree is a high-potential plant species with a deep root system for water absorption to prevent the salt rise-up, a large bushy cover to cover the surface area preventing the evaporation rate, and to detoxify the toxic salt ions by organic matter. It was known that planting gum trees at the ridge of the paddy field could protect it from salt erosion. However, plant diseases are the major problems of eucalyptus plantations by reducing plant yield. There were about 30 species of fungi that can cause eucalyptus diseases. Fungi can destroy eucalyptus plants in different stages of growth, ranging from seedling, cutting, and tree. The severity of diseases is different depending on fungal strains, susceptible plant variety, and climate in each area. It was reported that the most serious disease in *Eucalyptus* trees was leaf and shoot blight caused by *Pseudoplagiostroma eucalypti*, *Cylindrocladium reteaudii*, and *Teratosphaeria destructans* (Old et al., 2003). After the infection of these fungi, plants are weak and may be infected by other fungi such as *Kirramyces destructans*, *Cytospora* sp., *Lasiodiplodia theobromae*, and *Phomopsis* sp. to destruct stem and bark (Old et al., 2003). These fungal pathogens can cause severe symptoms and reduce plant yield dramatically. The bacterial pathogen, *Ralstonia solanacearum*, which causes eucalyptus wilt, is a secondary infection that occurs after fungi infect the plant in younger trees. Plant symptoms showed wilted and foliar necrosis or defoliation of the lower portion of the canopy (Santiago et al., 2014).

To the best of our knowledge, there is no report of studying the biodiversity of endophytic actinobacteria isolated from *Eucalyptus* grown in a salinity area. This is the first study of the biodiversity of endophytic actinobacteria from eucalyptus grown in a salinity area. The project’s goal is to find the beneficial endophytic actinobacteria that will promote growth of *Eucalyptus* trees and inhibit fungal pathogens in salinity conditions. This study focused on studying species biodiversity of endophytic actinobacteria from surface sterilized *E. camaldulensis* tissues grown at moderately and highly saline soil at Yang Talat district, Kalasin province, Thailand, studying antimicrobial activity of actinobacteria against important fungal pathogens, *Pseudoplagiostroma eucalypti* and opportunistic fungi causing leaf blight of eucalyptus*, Cladosporium* sp. *in vitro*, and studying plant growth-promoting properties of selected actinobacteria *in vitro.* We also tested the selected strain for seedling growth under salinity conditions and its ability to promote plant growth *in Planta*. Additionally, we studied the genomes of five selected strains from each plant sample.

## Materials and methods

### Plant sample collection

Root, twig, and leaf samples of *Eucalyptus camaldulensis* were collected from Ponsim Village, Tumbol Hua Nakham, Yang Talat District, Kalasin Province. Plant samples were collected in April 2022. We collected four plant samples from moderately saline soil and one from highly saline soil. Plant samples were collected, kept in paper bags, and processed within 24 hr. The information on salt contents in each area in this village is based on the previous study of Dr. Winya Dungkaew (data not reported). **Supplementary Figure S1** displays the latitudes and longitudes of each plant sample, EB, EC, EK, ES, and EW, along with its corresponding pictures.

### Collection of soil and salt concentration analysis

We collected soil samples from the eucalyptus growing area by digging 30 cm below the soil surface. Three soil samples were collected per sample site. Soil was air-dried for 2 weeks and then ground by mortar and pestle. Soil was sieved to discard contaminants by using a sieve size of 2 x 2 mm. The evaluation of soil salinity is measured by the electrical conductivity of a saturated soil extract (ECe) following the method of Rhoades et al. (1999). pH of soil samples was measured in Soil-H_2_O system (1:5) followed the method of FAO (2021) and soil solution was measured pH using pH meter.

### Isolation of endophytic actinobacteria

Samples of plants were surface sterilized using the method of Kaewkla and Franco (2013). Samples of roots, leaves, and twigs were properly cleaned using tap water and then in sterile RO water. Barks from twigs and roots were pulled off and dried on paper towels. The samples were surface sterilized by immersing them in 70% ethanol for five minutes, then in a solution of 6% sodium hypochlorite (freshly produced and 6% available chloride) for five minutes. The chemicals were then removed by washing them five times in sterile RO water. To break up plant tissues and slow the growth of endophytic fungi, the samples are soaked in 10% (w/v) NaHCO_3_ for ten minutes. They are then rinsed twice in sterile RO water. The surface sterilization is verified by aseptically rolling surface-sterilized plant tissues over each of the isolation media and tryptone soy agar. Each sample was chopped into small pieces measuring roughly 0.5 x 0.5 cm using a sterile scalpel. They were then crushed with a sterile mortar and pestle and plated on isolation media.

Each sterilized root, twig, and leaf sample was plated in triplicate onto four isolation media. Humic acid vitamin B agar (HVA) (Hayakawa and Nonomura, 1987), VL70 gellan gum with amino acid mixture (AA, comprising 17 amino acids), VL70 gellan gum with carboxymethyl cellulose, and starch casein nitrate agar (Mohseni et al., 2013) were used as the isolation media. 10 µg/mL of icotranazol was added as an antifungal agent. The VL 70 media composition is derived from previous works (Hudson et al., 1989; Joseph et al., 2003; Schoenborn et al., 2004). The plates were incubated at 30°C in small, airtight plastic boxes lined with wet towel paper to retain moisture for eight weeks. Colonies of actinobacteria were selected from isolation media and streaked onto half-strength potato dextrose agar (HPDA) plates to purify cultures. Pure cultures were maintained at 4°C on HPDA slants. The cells and spores were stored in 50% glycerol and frozen at -80°C for a long time.

### Identification of actinobacteria isolates and non-filamentous bacteria based on morphological characterization and molecular techniques

Morphological descriptions of all actinobacteria-like strains on ISP 2, ISP 3, and HPDA are regularly studied following the general guidelines of the International *Streptomyces* Project (ISP) (Shirling and Gottlieb, 1966). These include pigment or melanin production, the presence or absence of sporulation, and the color and growth characteristics of the mycelia. Non-filamentous bacteria grown on isolation plates were purified on nutrient agar and kept in nutrient agar slant at 4°C and prolonged-kept in 50% glycerol at -80°C. They were tested for Gram staining, and both Gram-staining positive and negative were kept for identification by 16S rRNA gene sequence analysis.

For the 16S rRNA gene sequence analysis, we chose strains that were typical of each morphological group of actinobacteria and non-filamentous bacteria. Genomic DNA was extracted from bacterial cells by using the GF1-DNA extraction kit (Vivantis). The 16S rRNA gene was amplified by PCR procedure using primer pairs 27f and 1492r and sequenced as described by Kaewkla and Franco (2013). The resultant sequences were compared to an online database using the EzbioCloud server (https://www.ezbiocloud.net; Yoon et al., 2017).

### Estimated relative abundance in genus level

The relative abundance (RA) at the genus level is the proportional number of each genus in each plant sample. The RA of each genus is expressed as


RA=(ni/N)×100

The variable ni represents the number of individuals within the same genus, while N represents the total number of individuals across all genera.

### Phylogenetic tree construction of 16S rRNA gene

The resulting sequences were compared to an online database using the EzTaxon-e service (Yoon et al., 2017). The 16S rRNA gene sequences of isolates from each genus were compared with the corresponding sequences of their closely related type strains available in GenBank/EMBL using CLUSTAL X (Thompson et al., 1997). The 16S rRNA gene phylogenetic trees of isolates belonging to genera *Streptomyces* and *Peterkaempfera* were generated using neighbor joining (NJ) (Saitou and Nei, 1987), maximum likelihood (ML) (Tamura and Nei, 1993), and maximum parsimony (MP) (Nei and Kumar, 2000) methods using the MEGA version 11 software tool (Tamura et al., 2021). *Embleya scabrisporus* DSM 41855^T^ was the outgroup.

The NJ tree computed the evolutionary distances using the Maximum Composite Likelihood method (Tamura et al., 2004), and the rate variation among sites was modeled with a gamma distribution (shape parameter=1). The ML tree was inferred by using the Maximum Likelihood method, and initial tree(s) for the heuristic search were obtained automatically by applying Neighbor-Join and BioNJ algorithms to a matrix of pairwise distances estimated using the Tamura-Nei model. The MP tree was obtained using the Tree-Bisection-Regrafting (TBR) algorithm with search level 1. The tree’s topology was assessed by a bootstrap approach (Felsenstein, 1985) including 1000 replications.

The 16S rRNA gene phylogenetic trees of isolates belonging to non-*Streptomyces* genera (9 genera) were generated using NJ, ML, and MP methods as described for the *Streptomyces* group with *Nocardia nova* JCM 6044^T^ as the outgroup.

### Antifungal and antibacterial assay *in vitro*

The dual culture method described by Kampapongsa and Kaewkla (2016) was used to test all actinobacteria to evaluate antifungal activity against *Pseudoplagiostroma eucalypti* LS6 and *Cladosporium* sp. LB1. The percent inhibition was calculated as follows:


$$\begin{array}{cc} \geq 75\text{ percent;Strong inhibition }\left(++++\right) & 51–74\text{ percent;Good inhibition }\left(+++\right) \end{array}$$

$$\begin{array}{cc} 26-50\text{ percent;Moderate inhibition}\left(++\right) & 1–25\text{ percent;Week inhibition }\left(+\right) \end{array}$$

< 0 percent; no inhibition(−)

Antibacterial activity against leaf wilt disease of eucalyptus, *Ralstonia solanacearum* TISTR 2069, was tested by the dual culture technique described by Kampapongsa and Kaewkla (2016).

### Screening for plant growth promoting traits of selected strains

Actinobacterial strains that showed good and strong inhibition against at least one tested fungal pathogen were selected to test for 1-aminocyclopropane-1-carboxylic acid (ACC) deaminase production, indole acetic acid (IAA) production, nitrogen fixation, phosphate solubilization, and cellulase production *in vitro.*

### ACC deaminase production and indole acetic acid (IAA production)

The capacity of actinobacteria to produce ACC deaminase was evaluated utilizing DF agar according to methods of Penrose and Glick (2003) and Kaewkla et al. (2022).

IAA production was investigated in accordance with the methodology of Kaewkla et al. (2022). One 8 mm actinomycete disc from a 7-day culture on HPDA was inoculated into ISP 2 broth with 0.2% (w/v) *L*-tryptophan and incubated on a rotary shaker at 150 rpm for 7 days in the dark at 28°C. The cultures were centrifuged at 6000 g for 10 minutes. The one milliliter of supernatant was mixed with two milliliters of Salkowski’s reagent and incubated in the dark for 30 minutes at ambient temperature. The reaction mixture’s absorbance was measured at 530 nm using a spectrophotometer. The concentration of IAA generated per milliliter of culture (µg/ml) was determined utilizing a standard curve of indole acetic acid (20–100 µg/ml).

### Phosphate solubilization and Nitrogen fixation assay

The capacity of actinobacteria for phosphate solubilization was evaluated using the methodology of Castagno et al. (2011). NBRIP agar was utilized, and a positive result was determined by measuring the halo zone surrounding the colony of each strain after a 14-day incubation duration. The method described by Baldani et al. (1997) was used to apply the nitrogen fixation *in vitro*. Nitrogen-free semi-solid (NFb) medium was used to inoculate actinobacteria and incubated it at 30°C for 14 days. The dark blue color of the media and the robust growth of the bacteria indicated a positive result. The culture was sub-cultured to the same media three times if it obtained a positive result.

### Cellulase production

The potential of actinobacteria to degrade cellulose was evaluated using ISP 2 agar supplemented with carboxymethyl cellulose 1%. Each bacterial isolate was cultivated for 14 days by spotting at the middle of the Petri dish. The hydrolysis of CMC was evaluated using a flood agar plate containing 0.1% Congo red, incubated for 10 minutes. Congo red was eliminated, and 10 ml of 1 M NaCl was flooded to the agar plate. The positive result demonstrated a halo zone surrounding the colony of actinobacteria.

### Growth on different sodium chloride concentration

Thirty-five selected strains of actinobacteria, which showed good PGP traits, were tested for their growth on different concentrations of sodium chloride at 1, 3, 5, 7, 9, and 11% (w/v) on ISP 2 medium pH 7.2. Actinobacterial cells were streaked in three lines on each agar plate and incubated at 30°C for 14 days. Growth was observed and evaluated at scores 0, 1, 2, and 3 for no growth, weak, moderate, and good growth, respectively. The experiment was done in triplicate.

### Seed germination test

*Streptomyces* strain EWL5.16, which showed good antifungal activity and PGP *in vitro*, was selected for a seed germination test with salinity stress in different sodium chloride concentrations. Also, the genome of this strain comprises many BGCs of antifungal compounds. *E. camaldulensis* seeds were gained from the forest research and development office, the Royal Forest Department, Thailand. Seeds were separated from seed debris by hand and soaked in RO water overnight and surface sterilized. Briefly, seeds were soaked in 70% ethanol for one min and rinsed three times with sterilized RO water (1 min in each wash). After that, seeds were immersed in 8% sodium hypochlorite for 5 mins and washed with sterilized RO water four times. Then, seeds were soaked in 2% sodium thiosulfate for 3 mins and washed in sterilized RO water. Strain EWL5.16 was cultured on HPDA for seven days, and spore suspension was prepared in sterilized RO water. Spore suspension was filtered and passed through a sterilized 10 mL syringe filled with sterilized cotton to separate the spores from the mycelia, and a hemocytometer was used to count the spores to 10^8^ spores per ml. After that, surface-sterilized *Eucalyptus* seeds were soaked in spore suspension for 1 hr., and RO water was used as a control.

Different concentrations of NaCl (0, 50, 100, 150, and 200 mM) were used in this study. Fifteen bacterized seeds from the above experiment were placed on sterilized Whatman No. 1 paper lined on a sterilized glass Petri dish (9 cm diameter) and moistened with 5 mL of each concentration of salt. The experiment was carried out in five replicates, and Petri dishes were located in a growth chamber in the dark two days before being exposed to light. After that, plates were exposed with cool daylight (12/12 light/dark cycles) at 28°C for 8 days. Seedlings were measured for seedling length (SL) (shoot and root lengths) at day 8. The experiment was done in a factorial design (2 factors with 5 levels of salt), and plates were arranged in a completely randomized design (CRD). Seeds were counted every day for eight days. The Seeding Length Vigor Index (SLVI) after eight days was calculated following some modifications from Zou et al. (2023).

(1) $$\begin{array}{cc} \text{Germination potential }\left(\text{GP}\%\right) & =\left(\text{m}1/\text{M}\right)\times 100 \end{array}$$

(2) $$\begin{array}{cc} \text{Germination rate }\left(\text{GR}\%\right) & =\left(\text{m}2/\text{M}\right)\times 100 \end{array}$$

(3) $$\begin{array}{cc} \text{Seed Length Vigor Index }\left(\text{SLVI}\right) & =\text{ GR}\times \text{SL} \end{array}$$

In the Equations 1–3, M is the total number of test seeds (M=15); m1 is the number of normally germinated seeds (root length ≥ 2 mm) within 3 days; m2 is the number of normally germinated seeds within 8 days; SL is the average seedling length (cm) after 8 days of germinated seeds. Germination potential (GP) in Equation 1 was followed (Zou et al., 2023), while germination rate (GR) in Equation 2 and SLVI in Equation 3 were described by Kerbab et al. (2021).

### Plant growth promoting *in planta*

Strain EWL5.16 was also selected to study the growth of eucalyptus seedlings in soil. The spore suspension of strain EWL5.16 was filtered and counted to 10^8^ spores/ ml according to a method described above. *E. camaldulensis* seeds were prepared and surface sterilized according to the method of seed germination test. Surface-sterilized eucalyptus seeds were soaked with strain EWL5.16 for 60 minutes and immersed in sterile RO water as a control experiment according to the method described above. Bacterized seeds were germinated in a sterilized plastic box (22 x 33 x 10 cm) lined with sterilized Whatman paper no. 1 and moistened with sterilized RO water pH 7.2 by placing 200 seeds per box for 7 days at 28°C in the dark.

Fine sand was sieved and autoclaved at 121°C for 30 minutes and left overnight and completely autoclaved for two other times. Sterilized sand was packed into 17 x 26 cm trays, 7 x 5 blocks per tray (3 x 3 cm per block). The experiment was conducted in six replicates per treatment (water and strain EWL5.16). The seedlings with even growth grown on filter paper prepared above were planted (2 seedlings per block). The experiment was arranged in a CRD. Plants were kept in the dark for 2 days before being exposed to cool daylight. The planting trays were kept in a growth chamber with cool daylight (12/12 light/dark cycles) at 28°C for fourteen days. Plants were watered with sterilized RO water one time per day. Although seedlings were surface sterilized, some seed-borne fungi destroyed seedlings, and plants started dying with root rots and decoloring leaves on day 7 after sowing. After fourteen days, survival seedlings with normal growth, considering plants with green leaves, were counted. Seedlings were gently taken from sand, and their roots were thoroughly washed to discard sand. Plants were measured for seedling lengths (shoot and root lengths) and plant fresh weight. As seedlings were still small, the dry weight could not be measured after drying the plant.

### Statistical analysis

The seed germination test applied a factorial design with two treatments (bacteria and without bacteria) and five levels of salt with a completely randomized design arrangement. IBM SPSS Statistics version 29 (Mahasarakham University License) was applied for statistical analysis. Data were submitted to normality and homogeneity of variances tests before the analysis of multivariate analysis of variance (MANOVA). The statistical analyses were carried out using GLM (General Linear Model) to determine the effectiveness of bacteria (seed priming with bacteria and salt) on seeding length vigor index. The Tukey method compared the means to identify a significant group with a *P*-value of less than 0.05 (*P*<0.05). For the PGP test in planta, data were submitted to normality and homogeneity of variances tests before analysis. The Independent-samples T-test was used to analyze data, and a *P*-value of less than 0.05 (*P<*0.05) showed significant differences.

### Genome sequencing, assembly, and annotation

As a model strain for genome sequencing, *Streptomyces* strain EKR5.2 was taken from sample EK. This strain isolated from plant sample grew in high-salinity soil and produced ACC deaminase and IAA. We chose strain ESS7.8 as the representative strain of *Streptomyces* from sample ES because it was good at killing fungal pathogens and was closely related to *Streptomyces ardesiacus* NRRL B-1773^T^, which was the most common species in four plant samples. We selected the representative strains of *Streptomyces* from the EC and EW strains, ECR2.10 and EWL5.16, for genome sequencing. These strains were a unique species of *Streptomyces* spp., closely related to *Streptomyces roietensis* WES2^T^. Also, these two strains showed activities of ACC deaminase and IAA production and good activity against fungal pathogens.

The representative strain of the non-*Streptomyces* genus from sample EW which contained the highest number of non-*Streptomyces* genera, *Micrococcus* strain EWR3.9.1, was selected for genome sequencing. We extracted the genomic DNA from *Streptomyces* strains EKR5.2, ESS7.8, ECR2.10, EWL5.16, and *Micrococcus* strain EWR3.9.1, using the previously described method as above. A short insert size library was prepared for genome sequencing of these five strains. Samples were sequenced at the Omics, Chulalongkorn University, using an Illumina NovaSeq sequencer (2 x 150 bp paired-end reads). We applied Unicycler (0.5.1) for *de novo* assembly of the reads (Wick et al., 2017). Genomes of these five strains were annotated using the Clusters of Orthologous Groups (COGs) on the Eggnog-Mapper database (V.2.1.9) (Cantalapiedra et al., 2021) and were evaluated for functional protein categories.

### Genome comparison study

The Genome-to-Genome Distance calculator (GGDC 2.1; BLAST + method) was used to analyze the digital DNA-DNA hybridization (dDDH) value between *Streptomyces* strains EKR5.2, ESS7.8, ECR2.10, EWL5.16, and *Micrococcus* strain EWR3.9.1 and its closely related type strains of the genus *Streptomyces* and *Micrococcus.* Formula 2 (identities/HSP length) was applied to the analysis (Meier-Kolthoff et al., 2013).

The phylogenetic tree of the genomes of *Streptomyces* strains EKR5.2, ESS7.8, ECR2.10, and EWL5.16 was constructed by the Type (Strain) Genome Server (TYGS) with other closely related type strains, with *Embleya scabrispora* DSM 41855^T^ as the outgroup (Lefort et al., 2015; Meier-Kolthoff and Göker, 2019). Also, the phylogenetic tree of the genome of *Micrococcus* strain EWR3.9.1 and its closely related type strains of the genus *Micrococcus* with *Tersicoccus phoenicis* DSM 30849^T^ as the outgroup was constructed by the Type (Strain) Genome Server (TYGS) (Lefort et al., 2015; Meier-Kolthoff and Göker, 2019).

The Average Nucleotide Identity values (ANI) blast (ANIb) and ANI MUMmer (ANIm) with pairwise genome alignment between *Streptomyces* strains EKR5.2, ESS7.8, ECR2.10, EWL5.16, and *Micrococcus* strain EWR3.9.1 and its closely related type strain of the genus *Streptomyces* and *Micrococcus* were analyzed using the JSpeicesWS web service (Richter and Rosselló-Móra, 2009; Richter et al., 2016).

### Biosynthetic Gene cluster analysis and *in silico* gene prediction

Secondary metabolite analysis Shell (anti-SMASH) version 7.0 (Blin et al., 2023) was used to predict biosynthetic gene clusters (BGCs) of *Streptomyces* strains EKR5.2, ESS7.8, ECR2.10, and EWL5.16 and *Micrococcus* strain EWR3.9.1. The genomes of these five strains were then annotated by the Clusters of Orthologous Groups (COGs). These genomes were then used to search for genes encoding for metabolite products that support plant growth, produce antibiotics and bioactive compounds, break down proteins, etc (Cantalapiedra et al., 2021).

## Results and discussion

### Soil salinity test and pH of soil

The EW, EC, ES, and EB soil samples were all moderately saline soil because their saturation extract conductivity was 4.5, 6.6, 7.1, and 7.4 dS/m, respectively. The pH of these soil samples was 4.6, 5.2, 4.2, and 5.0, respectively, which is strongly acid soil. The EK soil sample, on the other hand, was a strongly saline soil because its ECe value was 12 dS/m. The pH of the EK soil sample was 3.9, which was extremely acidic (**Table 1**). The pH of soil was classified as extremely acid (pH ≤ 4.0), very strongly or strongly acid soils (4.0 < pH ≤ 5.5), moderately acid, slightly acid and neutral soils (5.5 < pH ≤ 7.3), slightly alkaline and moderately alkaline soils (7.3 < pH ≤ 8.5), and strongly and very strongly alkaline soils (pH > 8.5) (Batjes, 1995; Mosley et al., 2024). It was reported that moderately saline soil (ECe 4–8 dS/m) affects yields of many crops, while strongly saline soil (ECe 8–16 dS/m) affects many plants, and only salt-tolerant crops can grow satisfactorily (Abrol et al., 1998). In this study, the soil sample of the EK plant was extremely acidic, and the soil of the other four plants (pH ≤ 5.5) was strongly acidic soil. Acidic soils primarily contribute to reductions in plant productivity due to deficits in important nutrients, including phosphorus (P), calcium (Ca), and magnesium (Mg). Moreover, metal toxicities, specifically from manganese (Mn) and aluminum (Al), intensify these difficulties. Soil acidification continues to be a significant concern in sustainable agriculture, negatively impacting soil health, crop yield, and environmental stability (Chen and Huang, 2024).

**Table 1:** The isolation numbers of endophytic actinobacteria isolated from five eucalyptus samples from three different media and plant parts.

Isolates	ECe of soil salinity (ds/m)	pH	Number		Part of plants			Isolation media				Total
			ST	Non ST	L	S	R	HV	SCNA	CMC	AA	
EB	7.4	5.0	123		28	26	69	37	23	30	33	123 (21.5%)
				2	2	0	0	0	2	0	0	2 (0.2%)
EC	6.6	5.2	102		40	24	38	34	29	20	19	102 (17.9%)
				0	0	0	0	0	0	0	0	0 (0)
EK	12	3.9	118		58	28	32	39	36	19	24	118 (20.7%)
				4	2	2	0	2	1	1	0	4 (0.7%)
ES	7.1	4.2	115		29	37	49	29	22	29	35	115 (20.0%)
				5	1	2	2	2	3	0	0	5 (0.8%)
EW	4.5	4.6	94		27	32	35	26	20	17	31	94 (16.6%)
				8	2	3	3	0	4	3	1	8 (1.6%)
Total			552 (96.7%)	19 (3.3%)	189 (33.1%)	154 (27.0%)	228 (39.9%)	169 (29.6%)	140 (24.5%)	119 (20.8%)	143 (25%)	571

ST, *Streptomyces*; L, leaf; S, twig; R, root. HVA, humic acid vitamin B agar; SCNA, starch casein nitrate agar; CMC, VL70 gellan gum with carboxymethyl cellulose; AA, VL70 gellan gum with amino acid mixture.

### Number of endophytic actinobacteria

Five hundred and seventy-one isolates of endophytic actinobacteria were isolated from five eucalyptus plants. Sample EB yielded the highest number of isolates at 125 (21.9%), with the rest of the isolates coming from samples EK (n=122), ES (n=120), EC (n=102), and EW (n=102). Most isolates came from roots (228), while the remaining isolates, 189 and 154, came from leaves and twigs, respectively. Three plant samples, EB, ES, and EW, gave the highest numbers of actinobacteria from roots, while plant samples EC and EK yielded the highest numbers of actinobacteria from leaves (**Table 1**). There were many studies reporting that most endophytic actinobacteria were obtained from root tissue (Kaewkla and Franco, 2013; Saikia et al., 2022; El-Akshar et al., 2024). The EK sample grows in highly saline soil (12 dS/m) and extremely acidic soil, which might affect actinobacteria to adapt to colonize in leaf tissues for their survival. This correlates with the work of Shan et al. (2018), who isolated most strains of endophytic actinobacteria from leaf and stem tissue. The other report showed that most isolates of endophytic actinobacteria were obtained from the leaf tissues of *E. camaldulensis* (Kaewkla and Franco, 2013).

### Identification of endophytic actinobacteria

**Figure 1:**
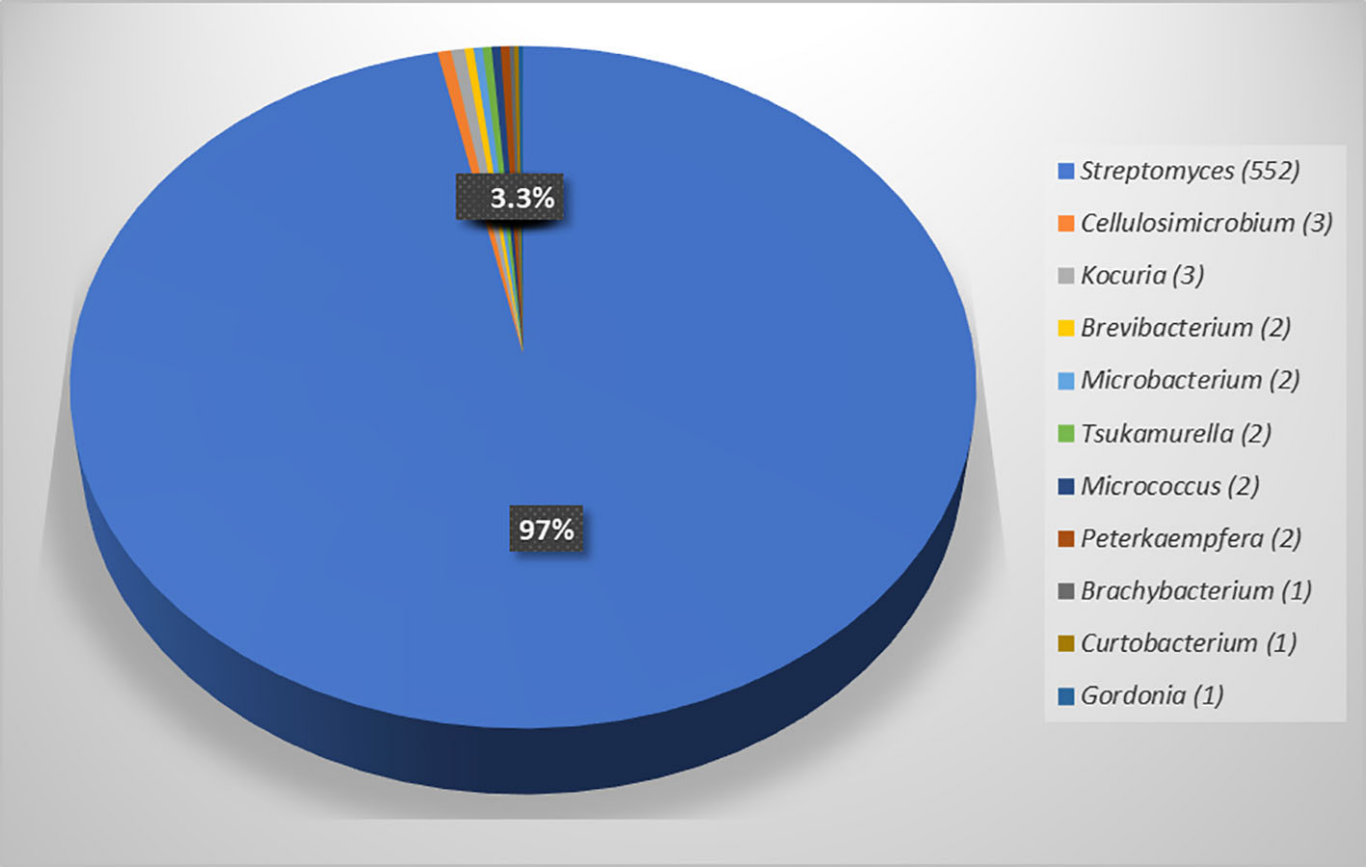
Numbers of endophytic actinobacteria in eleven genera and percentage of genus *Streptomyces* and non-*Streptomyces* genera isolated from five plant samples of *Eucalyptus camaldulensis.*.

Identification of all actinobacteria based on their morphology and 16S rRNA gene sequences showed that most isolates belonged to the genus *Streptomyces* (552, 96.7%), and the rest of the isolates were non-*Streptomyces* (19, 3.3%), comprising ten genera: *Cellulosimicrobium* (3), *Kocuria* (3), *Brevibacterium* (2), *Micrococcus* (2), *Microbacterium* (2)*, Peterkaempfera* (2), *Tsukamurella* (2), *Brachybacterium* (1), *Curtobacterium* (1), and *Gordonia* (1) (**Table 2**; **Figure 1**). The details of the closest match of each isolate are shown in **Table 2**. It was found that *Streptomyces* was the most common genus of endophytic actinobacteria, which was also found in other studies (Himaman et al., 2016; Shan et al., 2018; Hazarika et al., 2022; Saikia et al., 2022).

**Table 2:** The three closest matches of type strains of endophytic actinobacteria isolated from surface-sterilized tissues based on 16S rRNA gene similarity.

Isolate/genus^#^	The 1^st^ closest match	%	The 2^nd^ closest match	%	The 3rd closest match	%
*Streptomyces*						
	*Streptomyces rochei* NRRL B-2410^T^ (25 strains)					
EBS5.2	*Streptomyces rochei* NRRL B-2410^T^	98.9	*Streptomyces tuirus* NBRC 15617^T^	98.3	*Streptomyces djakartensis* NBRC 15409^T^	98.3
EBL7.9	*Streptomyces rochei* NRRL B-2410^T^	99.8	*Streptomyces mutabilis* NBRC 12800^T^	99.5	*Streptomyces tuirus* NBRC 15617^T^	99.4
EBS5.1	*Streptomyces rochei* NRRL B-2410^T^	99.7	*Streptomyces mutabilis* NBRC 12800^T^	99.3	*Streptomyces tuirus* NBRC 15617^T^	99.0
EBR3.2	*Streptomyces rochei* NRRL B-2410^T^	99.6	*Streptomyces mutabilis* NBRC 12800^T^	99.4	*Streptomyces tuirus* NBRC 15617^T^	99.0
ECS6.12	*Streptomyces rochei* NRRL B-2410^T^	100	*Streptomyces mutabilis* NBRC 12800^T^	99.8	*Streptomyces tuirus* NBRC 15617^T^	99.3
ECR5.32	*Streptomyces rochei* NRRL B-2410^T^	99.9	*Streptomyces mutabilis* NBRC 12800^T^	99.5	*Streptomyces tuirus* NBRC 15617^T^	99.2
EK7.15	*Streptomyces rochei* NRRL B-2410^T^	100	*Streptomyces mutabilis* NBRC 12800^T^	99.8	*Streptomyces tuirus* NBRC 15617^T^	99.3
EKL7.21	*Streptomyces rochei* NRRL B-2410^T^	99.6	*Streptomyces mutabilis* NBRC 12800^T^	99.2	*Streptomyces tuirus* NBRC 15617^T^	98.9
EKL6.13	*Streptomyces rochei* NRRL B-2410^T^	100	*Streptomyces mutabilis* NBRC 12800^T^	99.6	*Streptomyces tuirus* NBRC 15617^T^	99.3
EKR6.13	*Streptomyces rochei* NRRL B-2410^T^	100	*Streptomyces mutabilis* NBRC 12800^T^	99.6	*Streptomyces tuirus* NBRC 15617^T^	99.3
EKS3.5	*Streptomyces rochei* NRRL B-2410^T^	99.4	*Streptomyces mutabilis* NBRC 12800^T^	99.0	*Streptomyces tuirus* NBRC 15617^T^	98.7
EKS3.12	*Streptomyces rochei* NRRL B-2410^T^	99.4	*Streptomyces mutabilis* NBRC 12800^T^	99.0	*Streptomyces tuirus* NBRC 15617^T^	98.8
ESL8.11	*Streptomyces rochei* NRRL B-2410^T^	99.3	*Streptomyces mutabilis* NBRC 12800^T^	99.1	*Streptomyces tuirus* NBRC 15617^T^	98.7
ESL3.11	*Streptomyces rochei* NRRL B-2410^T^	97.8	*Streptomyces mutabilis* NBRC 12800^T^	97.6	*Streptomyces tuirus* NBRC 15617^T^	97.0
ESR5.20	*Streptomyces rochei* NRRL B-2410^T^	99.9	*Streptomyces mutabilis* NBRC 12800^T^	99.1	*Streptomyces tuirus* NBRC 15617^T^	99.1
ESL4.13	*Streptomyces rochei* NRRL B-2410^T^	100	*Streptomyces mutabilis* NBRC 12800^T^	99.8	*Streptomyces tuirus* NBRC 15617^T^	99.3
EWR8.17	*Streptomyces rochei* NRRL B-2410^T^	99.7	*Streptomyces mutabilis* NBRC 12800^T^	99.3	*Streptomyces tuirus* NBRC 15617^T^	99.0
EWS5.2	*Streptomyces rochei* NRRL B-2410^T^	99.5	*Streptomyces mutabilis* NBRC 12800^T^	99.1	*Streptomyces tuirus* NBRC 15617^T^	98.8
EWR7.9	*Streptomyces rochei* NRRL B-2410^T^	99.3	*Streptomyces mutabilis* NBRC 12800^T^	99.0	*Streptomyces tuirus* NBRC 15617^T^	98.6
EWL3.20	*Streptomyces rochei* NRRL B-2410^T^	100	*Streptomyces mutabilis* NBRC 12800^T^	99.8	*Streptomyces tuirus* NBRC 15617^T^	99.3
EWRD8.25	*Streptomyces rochei* NRRL B-2410^T^	98.8	*Streptomyces mutabilis* NBRC 12800^T^	98.3	*Streptomyces tuirus* NBRC 15617^T^	98.1
EWS1.12	*Streptomyces rochei* NRRL B-2410^T^	98.9	*Streptomyces mutabilis* NBRC 12800^T^	98.5	*Streptomyces tuirus* NBRC 15617^T^	98.2
EWL6.4	*Streptomyces rochei* NRRL B-2410^T^	100	*Streptomyces mutabilis* NBRC 12800^T^	99.6	*Streptomyces tuirus* NBRC 15617^T^	99.3
EWR7.15	*Streptomyces rochei* NRRL B-2410^T^	98.5	*Streptomyces mutabilis* NBRC 12800^T^	98.1	*Streptomyces tuirus* NBRC 15617^T^	97.8
EWS3.17.1	*Streptomyces rochei* NRRL B-2410^T^	100	*Streptomyces mutabilis* NBRC 12800^T^	99.6	*Streptomyces tuirus* NBRC 15617^T^	99.4
	*Streptomyces ardesiacus* NRRL B-1773^T^ (13 strains)					
ECL8.1	*Streptomyces ardesiacus* NRRL B-1773^T^	99.8	*Streptomyces coelicoflavus* NBRC 15399^T^	99.1	*Streptomyces salinarius* SS06011^T^	98.7
EKR1.2	*Streptomyces ardesiacus* NRRL B-1773^T^	99.0	*Streptomyces salinarius* SS06011^T^	98.9	*Streptomyces coelicoflavus* NBRC 15399^T^	98.8
ESS7.8	*Streptomyces ardesiacus* NRRL B-1773^T^	98.8	*Streptomyces salinarius* SS06011^T^	97.9	*Streptomyces coelicoflavus* NBRC 15399^T^	97.7
ESS7.22	*Streptomyces ardesiacus* NRRL B-1773^T^	99.8	*Streptomyces coelicoflavus* NBRC 15399^T^	99.0	*Streptomyces salinarius* SS06011^T^	98.9
ESL4.15	*Streptomyces ardesiacus* NRRL B-1773^T^	99.8	*Streptomyces coelicoflavus* NBRC 15399^T^	99.1	*Streptomyces chilikensis* RC 1830^T^	99.0
ESS5.21	*Streptomyces ardesiacus* NRRL B-1773^T^	98.6	*Streptomyces salinarius* SS06011^T^	97.6	*Streptomyces coelicoflavus* NBRC 15399^T^	97.5
ESL5.5	*Streptomyces ardesiacus* NRRL B-1773^T^	99.8	*Streptomyces salinarius* SS06011^T^	98.8	*Streptomyces chilikensis* RC 1830^T^	98.7
ESR1.3	*Streptomyces ardesiacus* NRRL B-1773^T^	99.8	*Streptomyces coelicoflavus* NBRC 15399^T^	99.2	*Streptomyces chilikensis* RC 1830^T^	99.0
ESR5.15	*Streptomyces ardesiacus* NRRL B-1773^T^	98.9	*Streptomyces coelicoflavus* NBRC 15399^T^	98.9	*Streptomyces chilikensis* RC 1830^T^	98.2
EWS5.6	*Streptomyces ardesiacus* NRRL B-1773^T^	99.9	*Streptomyces coelicoflavus* NBRC 15399^T^	99.2	*Streptomyces chilikensis* RC 1830^T^	99.2
EWR1.23	*Streptomyces ardesiacus* NRRL B-1773^T^	99.2	*Streptomyces coelicoflavus* NBRC 15399^T^	98.7	*Streptomyces nigra* 452^T^	98.6
EWR1.13	*Streptomyces ardesiacus* NRRL B-1773^T^	98.9	*Streptomyces chilikensis* RC 1830^T^	98.4	*Streptomyces coelicoflavus* NBRC 15399^T^	98.3
EWS8.8	*Streptomyces ardesiacus* NRRL B-1773^T^	99.5	*Streptomyces coelicoflavus* NBRC 15399^T^	98.7	*Streptomyces chilikensis* RC 1830^T^	98.3
	*Streptomyces thermoviolaceus* subsp. *apingens* DSM 41392^T^ (10 strains)					
ECS7.17	*Streptomyces thermoviolaceus* subsp. *apingens* DSM 41392^T^	99.2	*Streptomyces mexicanus* CH-M-1035^T^	98.9	*Streptomyces chromofuscus* NBRC 12851^T^	98.7
ECS7.22	*Streptomyces thermoviolaceus* subsp. *apingens* DSM 41392^T^	98.8	*Streptomyces mexicanus* CH-M-1035^T^	98.3	*Streptomyces chromofuscus* NBRC 12851^T^	98.3
ECL5.20	*Streptomyces thermoviolaceus* subsp. *apingens* DSM 41392^T^	99.2	*Streptomyces mexicanus* CH-M-1035^T^	98.9	*Streptomyces chromofuscus* NBRC 12851^T^	98.7
ECL8.19	*Streptomyces thermoviolaceus* subsp. *apingens* DSM 41392^T^	99.0	*Streptomyces mexicanus* CH-M-1035^T^	98.8	*Streptomyces chromofuscus* NBRC 12851^T^	98.4
EKL1.1	*Streptomyces thermoviolaceus* subsp. *apingens* DSM 41392^T^	98.4	*Streptomyces mexicanus* CH-M-1035^T^	98.4	*Streptomyces chromofuscus* NBRC 12851^T^	98.4
EKS5.11	*Streptomyces thermoviolaceus* subsp. *apingens* DSM 41392^T^	99.1	*Streptomyces mexicanus* CH-M-1035^T^	98.8	*Streptomyces thermocarboxydovorans* DSM 44296^T^	98.5
ESR3.4	*Streptomyces thermoviolaceus* subsp. *apingens* DSM 41392^T^	98.1	*Streptomyces chromofuscus* NBRC 12851^T^	98.1	*Streptomyces mexicanus* CH-M-1035^T^	97.8
ESR4.19	*Streptomyces thermoviolaceus* subsp. *apingens* DSM 41392^T^	98.2	*Streptomyces chromofuscus* NBRC 12851^T^	98.2	*Streptomyces mexicanus* CH-M-1035^T^	98.0
ESR4.20	*Streptomyces thermoviolaceus* subsp. *apingens* DSM 41392^T^	98.9	*Streptomyces mexicanus* CH-M-1035^T^	98.6	*Streptomyces chromofuscus* NBRC 12851^T^	98.6
EWL8.17	*Streptomyces thermoviolaceus* subsp. *apingens* DSM 41392^T^	99.1	*Streptomyces mexicanus* CH-M-1035^T^	98.7	*Streptomyces chromofuscus* NBRC 12851^T^	98.7
	*Streptomyces thermocarboxydus* DSM 44293^T^ (7 strains)					
ECL6.4	*Streptomyces thermocarboxydus* DSM 44293^T^	99.5	*Streptomyces lusitanus* NBRC 13464^T^	99.4	*Streptomyces coerulescens* ISP 5146^T^	98.9
ECL7.29	*Streptomyces thermocarboxydus* DSM 44293^T^	99.4	*Streptomyces lusitanus* NBRC 13464^T^	98.9	*Streptomyces coerulescens* ISP 5146^T^	98.3
EKL5.11	*Streptomyces thermocarboxydus* DSM 44293^T^	99.9	*Streptomyces lusitanus* NBRC 13464^T^	99.8	*Streptomyces coerulescens* ISP 5146^T^	99.2
ESS3.9	*Streptomyces thermocarboxydus* DSM 44293^T^	99.8	*Streptomyces lusitanus* NBRC 13464^T^	99.4	*Streptomyces indiaensis* NBRC 13964^T^	99.0
ESS5.2	*Streptomyces thermocarboxydus* DSM 44293^T^	99.6	*Streptomyces lusitanus* NBRC 13464^T^	99.2	*Streptomyces indiaensis* NBRC 13964^T^	98.8
ESR2.15	*Streptomyces thermocarboxydus* DSM 44293^T^	99.3	*Streptomyces werraensis* NBRC 13404^T^	99.0	*Streptomyces cellulosae* NBRC 13027^T^	99.0
ESR5.16	*Streptomyces thermocarboxydus* DSM 44293^T^	99.8	*Streptomyces lusitanus* NBRC 13464^T^	99.5	*Streptomyces indiaensis* NBRC 13964^T^	98.8
	*Streptomyces violaceorectus* NBRC 13102^T^ (6 strains)					
ESS8.20	*Streptomyces violaceorectus* NBRC 13102^T^	99.7	*Streptomyces bikiniensis* NRRL B-1049^T^	99.4	*Streptomyces cinereoruber* NBRC 12756^T^	99.1
ESR5.40	*Streptomyces violaceorectus* NBRC 13102^T^	99.7	*Streptomyces bikiniensis* NRRL B-1049^T^	99.4	*Streptomyces cinereoruber* NBRC 12756^T^	99.1
ESL7.2	*Streptomyces violaceorectus* NBRC 13102^T^	100	*Streptomyces bikiniensis* NRRL B-1049^T^	99.7	*Streptomyces cinereoruber* NBRC 12756^T^	99.4
ESL1.1	*Streptomyces violaceorectus* NBRC 13102^T^	100	*Streptomyces bikiniensis* NRRL B-1049^T^	99.7	*Streptomyces cinereoruber* NBRC 12756^T^	99.4
ESL6.5	*Streptomyces violaceorectus* NBRC 13102^T^	99.7	*Streptomyces bikiniensis* NRRL B-1049^T^	99.4	*Streptomyces cinereoruber* NBRC 12756^T^	99.1
ESL3.1	*Streptomyces violaceorectus* NBRC 13102^T^	99.7	*Streptomyces bikiniensis* NRRL B-1049^T^	99.4	*Streptomyces cinereoruber* NBRC 12756^T^	99.1
	*Streptomyces jiujiangensis* JXJ 0074^T^ (5 strains)					
EB1.5	*Streptomyces jiujiangensis* JXJ 0074^T^	95.7	*Streptomyces shenzhenensis* subsp. *oryzicola* W18L9^T^	95.5	*Streptomyces shenzhenensis* subsp. *shenzhenensis* 172115^T^	95.4
EBR3.8	*Streptomyces jiujiangensis* JXJ 0074^T^	99.2	*Streptomyces shenzhenensis* subsp. *oryzicola* W18L9^T^	99.0	*Streptomyces shenzhenensis* subsp. *shenzhenensis* 172115^T^	98.9
EBR4.16	*Streptomyces jiujiangensis* JXJ 0074^T^	99.3	*Streptomyces shenzhenensis* subsp. *oryzicola* W18L9^T^	99.1	*Streptomyces shenzhenensis* subsp. *shenzhenensis* 172115^T^	99.1
ESS7.7	*Streptomyces jiujiangensis* JXJ 0074^T^	99.4	*Streptomyces shenzhenensis* subsp. *oryzicola* W18L9^T^	99.2	*Streptomyces shenzhenensis* subsp. *shenzhenensis* 172115^T^	99.1
ESS3.11	*Streptomyces jiujiangensis* JXJ 0074^T^	96.8	*Streptomyces shenzhenensis* subsp. *oryzicola* W18L9^T^	96.5	*Streptomyces lucensis* NBRC 13056^T^	96.4
	*Streptomyces chromofuscus* NBRC 12851^T^ (4 strains)					
ESR3.38	*Streptomyces chromofuscus* NBRC 12851^T^	99.0	*Streptomyces cadmiisoli* ZFG47^T^	98.9	*Streptomyces gossypiisoli* TRM 44567^T^	98.8
ESR7.3	*Streptomyces chromofuscus* NBRC 12851^T^	99.4	*Streptomyces gossypiisoli* TRM 44567^T^	99.1	*Streptomyces cadmiisoli* ZFG47^T^	98.7
ESR3.26	*Streptomyces chromofuscus* NBRC 12851^T^	99.3	*Streptomyces gossypiisoli* TRM 44567^T^	98.9	*Streptomyces cadmiisoli* ZFG47^T^	98.8
ESR3.25	*Streptomyces chromofuscus* NBRC 12851^T^	98.9	*Streptomyces cadmiisoli* ZFG47^T^	98.6	*Streptomyces fumigatiscleroticus* NBRC 12999^T^	98.6
	*Streptomyces coelicoflavus* NBRC 15399^T^ (5 strains)					
ECR5.7	*Streptomyces coelicoflavus* NBRC 15399^T^	99.4	*Streptomyces ardesiacus* NRRL B-1773^T^	99.2	*Streptomyces fragilis* NRRL 2424^T^	99.1
ECL7.10	*Streptomyces coelicoflavus* NBRC 15399^T^	99.3	*Streptomyces ardesiacus* NRRL B-1773^T^	99.1	*Streptomyces fragilis* NRRL 2424^T^	99.0
ECS5.14	*Streptomyces coelicoflavus* NBRC 15399^T^	99.4	*Streptomyces ardesiacus* NRRL B-1773^T^	99.2	*Streptomyces fragilis* NRRL 2424^T^	99.1
ESR6.16	*Streptomyces coelicoflavus* NBRC 15399^T^	99.4	*Streptomyces ardesiacus* NRRL B-1773^T^	99.3	*Streptomyces fragilis* NRRL 2424^T^	99.3
EWR6.5	*Streptomyces coelicoflavus* NBRC 15399^T^	98.9	*Streptomyces ardesiacus* NRRL B-1773^T^	98.8	*Streptomyces fragilis* NRRL 2424^T^	98.8
	*Streptomyces albogriseolus* NRRL B-1305^T^ (3 strains)					
EWS6.1	*Streptomyces albogriseolus* NRRL B-1305^T^	100	*Streptomyces griseoincarnatus* LMG 19316^T^	99.6	*Streptomyces labedae* NBRC 15864^T^	99.6
EWS6.11	*Streptomyces albogriseolus* NRRL B-1305^T^	100	*Streptomyces griseoincarnatus* LMG 19316^T^	99.7	*Streptomyces labedae* NBRC 15864^T^	99.7
EWR1.4	*Streptomyces albogriseolus* NRRL B-1305^T^	99.5	*Streptomyces coeruleorubidus* ISP 5145^T^	99.3	*Streptomyces nigra* 452^T^	99.3
	*Streptomyces nigra* 452^T^					
ECN6	*Streptomyces nigra* 452^T^	99.2	*Streptomyces longispororuber* NBRC 13488^T^	98.9	*Streptomyces coerulescens* ISP 5146^T^	98.9
ECR7.1	*Streptomyces nigra* 452^T^	99.3	*Streptomyces longispororuber* NBRC 13488^T^	99.0	*Streptomyces coerulescens* ISP 5146^T^	99.0
	*Streptomyces* sp*iralis* NBRC 14215^T^					
ECR3.25	*Streptomyces* sp*iralis* NBRC 14215^T^	99.9	*Streptomyces fumigatiscleroticus* NBRC 12999^T^	99.0	*Streptomyces minutiscleroticus* NBRC 13000^T^	99.0
ESR1.8	*Streptomyces* sp*iralis* NBRC 14215^T^	99.5	*Streptomyces sennicomposti* RCPT1-4^T^	99.3	*Streptomyces cellulosae* NBRC 13027^T^	99.0
	*Streptomyces roietensis*WES2^T^					
ECR2.10	*Streptomyces roietensis*WES2^T^	98.8	*Streptomyces leeuwenhoekii* C34^T^	98.7	*Streptomyces pluripotens* MUSC135^T^	98.7
EWL5.16	*Streptomyces roietensis*WES2^T^	98.8	*Streptomyces leeuwenhoekii* C34^T^	98.7	*Streptomyces pluripotens* MUSC135^T^	98.7
	*Streptomyces yeochonensis* CN732^T^					
ESL2.7	*Streptomyces yeochonensis* CN732^T^	98.0	*Streptomyces epipremni* PRB2–1^T^	97.9	*Streptomyces rubida* 13C15^T^	97.8
ESS2.7	*Streptomyces yeochonensis* CN732^T^	98.5	*Streptomyces rubida* 13C15^T^	98.3	*Streptomyces cocklensis* BK168^T^	98.0
	Other single species					
EBS7.9	*Streptomyces viridochromogenes* NBRC 3113^T^	99.8	*Streptomyces paradoxus* NBRC 14887^T^	99.3	*Streptomyces ambofaciens* ATCC 23877^T^	99.2
EBS5.3	*Streptomyces spinoverrucosus* NBRC 14228^T^	99.1	*Streptomyces lusitanus* NBRC 13464^T^	99.0	*Streptomyces thermocarboxydus* DSM 44293^T^	98.9
EBR7.15	*Streptomyces shenzhenensis* 172115^T^	98.7	*Streptomyces jiujiangensis* JXJ 0074^T^	98.4	*Streptomyces hyaluromycini* NBRC 110483^T^	98.4
ECR3.35	*Streptomyces echinatus* NBRC 12763^T^	98.9	*Streptomyces actinomycinicus* RCU-197^T^	98.7	*Streptomyces crystallinus* NBRC 15401^T^	98.4
ECL5.16	*Streptomyces althioticus* NRRL B-3981^T^	99.7	*Streptomyces griseoflavus* LMG 19344^T^	99.6	*Streptomyces griseoincarnatus* LMG 19316^T^	99.6
ECR3.81	*Streptomyces hyderabadensis* OU-40^T^	99.7	*Streptomyces parvulus* NBRC 13193^T^	99.4	*Streptomyces malachitospinus* NBRC 101004^T^	99.3
ECR3.3	*Streptomyces drozdowiczii* NBRC 101007^T^	99.3	*Streptomyces laculatispora* BK166^T^	99.0	*Streptomyces brevispora* BK160^T^	99.0
EKS8.28	*Streptomyces chiangmaiensis* TA4-1^T^	98.7	*Streptomyces lannensis* TA4-8^T^	98.4	*Streptomyces caeni* HA15955^T^	98.4
EKR5.2	*Streptomyces lannensis* TA4-8^T^	98.2	*Streptomyces chiangmaiensis* TA4-1^T^	96.8	*Streptomyces leeuwenhoekii* C34^T^	96.6
EKR7.5	*Streptomyces albus* NBRC 13014^T^	99.6	*Streptomyces gibsonii* NRRL B-1335^T^	99.6	*Streptomyces almquistii* NRRL B-1685^T^	99.6
EKS4.1	*Streptomyces pratensis* ch24^T^	99.6	*Streptomyces silvae* For3^T^	99.5	*Streptomyces badius* NRRL B-2567^T^	99.4
EKR6.15	*Streptomyces ginkgonis* KM-1-2^T^	99.2	*Streptomyces carpaticus* NBRC 15390^T^	99.2	*Streptomyces harbinensis* NEAU-Da3^T^	98.8
EWR6.5.1	*Streptomyces puniceus* NBRC 1281^T^	100	*Streptomyces microflavus* NBRC 13062^T^	99.8	*Streptomyces fulvorobeus* NBRC 15897^T^	99.8
EWL3.9	*Streptomyces chartreusis* NBRC 12753^T^	98.8	*Streptomyces kunmingensis* NBRC 14463^T^	98.6	*Streptomyces osmaniensis* OU-63^T^	98.5
EWS3.1	*Streptomyces griseorubiginosus* DSM 40469^T^	99.4	*Streptomyces cinnabarigriseus* JS360^T^	98.3	*Streptomyces griseochromogenes* ATCC 14511^T^	98.2
Non-*Streptomyces*						
ESS3.1	*Peterkaempfera griseoplanus* NRRL B-3064^T^	99.6	*Peterkaempfera bronchialis* DSM 106435^T^	98.5	*Kitasatospora paracochleata* IFO 14769^T^	97.2
ESS2.4	*Peterkaempfera griseoplanus* NRRL B-3064^T^	99.6	*Peterkaempfera bronchialis* DSM 106435^T^	98.3	*Streptomyces baliensis* ID03-0915^T^	97.7
ESR6.10	*Brachybacterium phenoliresistens* phenol-A^T^	99.6	*Brachybacterium sacelli* LMG 20345^T^	97.3	*Brachybacterium kimchi* CBA3104^T^	97.0
ESR5.3	*Brevibacterium luteolum* CF87^T^	99.5	*Brevibacterium gallinarum* Re57^T^	99.5	*Brevibacterium otitidis *NCFB 3053^T^	98.3
EWS3.12	*Cellulosimicrobium cellulans* LMG 16121^T^	99.6	*Cellulosimicrobium funkei* ATCC BAA-886^T^	99.6	*Cellulosimicrobium composti* BIT-GX5^T^	99.3
EWL6.1	“*Curtobacterium oceanosedimentum*” ATCC 31317^T^	99.9	*Curtobacterium* *citreum* JCM 1345^T^	99.8	*Curtobacterium citri* JCM 34829^T^	99.8
EKL3.2	*Gordonia terrae* NBRC 100016^T^	99.6	*Gordonia didemni* B204^T^	99.8	*Gordonia hongkongensis* HKU50^T^	99.6
EBL3.4	*Kocuria arsenatis* CM1E1^T^	99.9	*Kocuria rhizophila* TA68^T^	99.8	*Kocuria tytonis* 442^T^	99.5
EBAL27	*Kocuria arsenatis* CM1E1^T^	99.8	*Kocuria rhizophila* TA68^T^	99.7	*Kocuria tytonis* 442^T^	99.4
EWS3.8.2	*Kocuria arsenatis* CM1E1^T^	99.8	*Kocuria rhizophila* TA68^T^	99.7	*Kocuria tytonis* 442^T^	99.4
EKS6.13	*Microbacterium album* SYSU D8007^T^	98.3	*Microbacterium paludicola* US15^T^	98.0	*Microbacterium marinilacus* YM11-607^T^	97.9
EKS8.26	*Microbacterium album* SYSU D8007^T^	98.5	*Microbacterium paludicola* US15^T^	98.3	*Microbacterium marinilacus* YM11-607^T^	98.0
EWR3.9.1	*Micrococcus luteus* NCTC 2665^T^	99.5	*Micrococcus endophyticus* YIM 56238^T^	99.3	*Micrococcus porci* KD337-16^T^	98.9
EKL2.3	*Tsukamurella paurometabola* DSM 20162^T^	98.7	*Tsukamurella inchonensis* DSM 44067^T^	98.6	*Tsukamurella sputi* HKU70^T^	98.5
EWR2.8	*Tsukamurella paurometabola* DSM 20162^T^	99.0	*Tsukamurella inchonensis* DSM 44067^T^	98.9	*Tsukamurella sputi* HKU70^T^	98.6

^#^The GenBank accession numbers are presented in **Supplementary Table S12**.

### 16S rRNA gene and phylogenetic tree analysis of *Streptomyces* and *Peterkaempfera*

**Figure 2:**
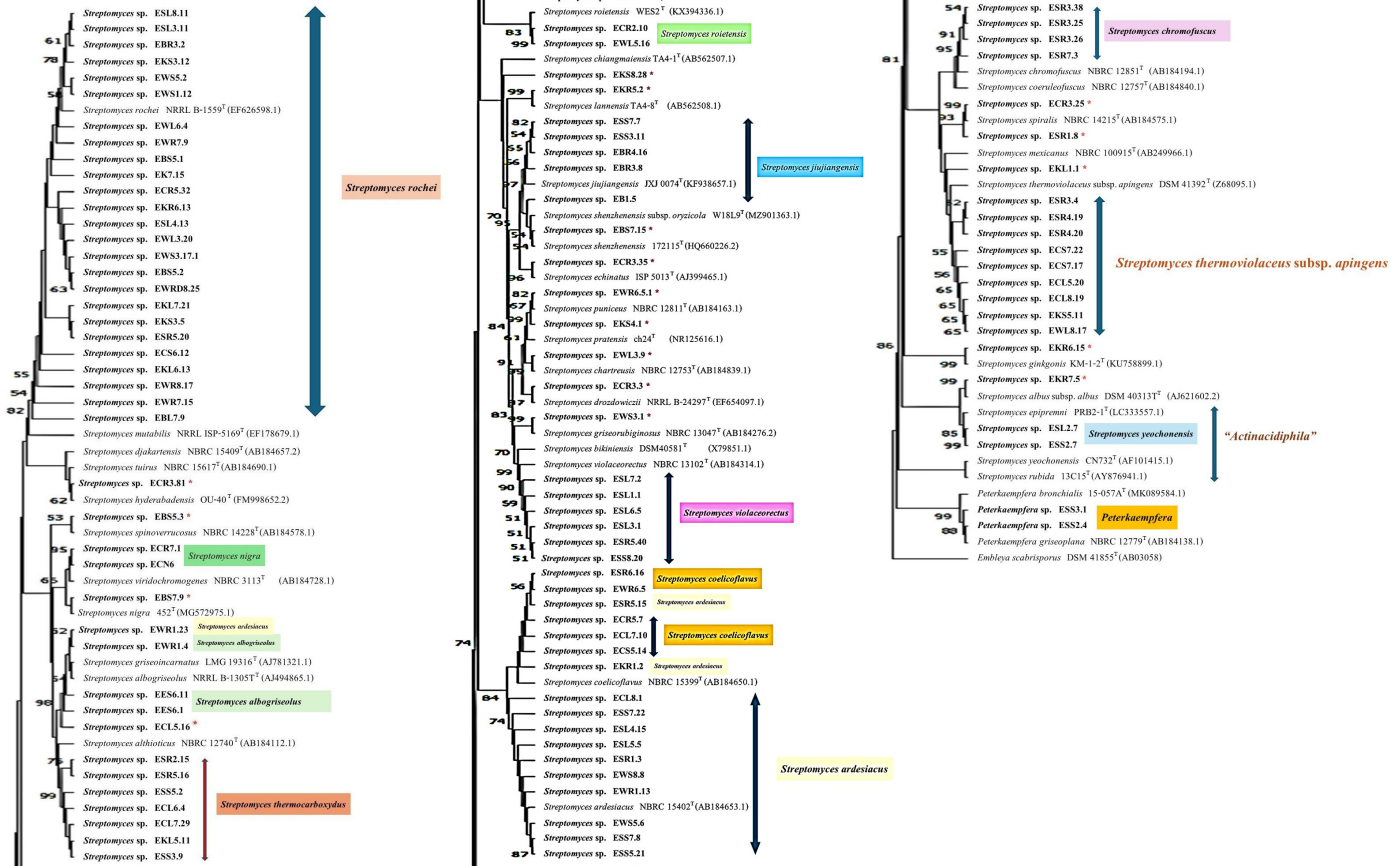
The neighbor-joining phylogenetic tree is based on 16S rRNA gene sequences of 101 strains of *Streptomyces* and 2 strains of the genus *Peterkaemfera* and their closely related members in the genera *Streptomyces* and *Peterkaemfera* and *Embleya scabrisporus* DSM 41855^T^ as the out-group. Bootstrap values based on 1000 replicates are shown at the branch nodes. *indicate a single strain of a unique species of *Streptomyces.*.

One hundred and one isolates of the *Streptomyces* morphological groups were sequenced, and the 16S rRNA gene sequence analysis showed that these strains shared 16S rRNA gene similarity with the validly published species in the genus *Streptomyces* between 95.7% and 100% (**Table 3**). NJ, ML, and MP phylogenetic trees of the 16S rRNA gene constructed of all the strains of *Streptomyces* and *Peterkaempfera* along with their most closely related type strains are shown in **Figures 2**; **Supplementary Figures S2**, **S3**. The result showed that most of the *Streptomyces* strains from eucalyptus tissues position in the same clade or cluster with their closest type strains on three phylogenetic trees.

**Table 3:** Numbers of endophytic actinobacteria isolated from each plant and each medium between week 1 and week 8.

Medium/plant	Numbers of isolates in each week																
	1	1	2	2	3	3	4	4	5	5	6	6	7	7	8	8	Total number
	ST	NST	ST	NST	ST	NST	ST	NST	ST	NST	ST	NST	ST	NST	ST	NST	
EB																	
HVA	2	0	2	0	11	0	5	0	2	0	3	0	5	0	7	0	37
SCNA	1	0	1	1	0	1	3	0	8	0	4	0	1	0	5	0	25
CMC	2	0	2	0	5	0	8	0	3	0	3	0	2	0	5	0	30
AA	4	0	2	0	3	0	5	0	7	0	1	0	3	0	8	0	33
Total EB	9	0	7	1	19	1	21	0	20	0	11	0	11	0	25	0	125
**EC**																	0
HVA	1	0	0	0	5	0	5	0	5	0	6	0	8	0	4	0	34
SCNA	3	0	1	0	7	0	5	0	4	0	6	0	2	0	1	0	29
CMC	1	0	1	0	4	0	0	0	1	0	3	0	7	0	3	0	20
AA	0	0	2	0	2	0	1	0	5	0	4	0	4	0	1	0	19
Total EC	5	0	4	0	18	0	11	0	15	0	19	0	21	0	9	0	102
**EK**																	0
HVA	0	0	1	0	3	0	4	0	9	0	3	1	12	0	7	1	41
SCNA	1	0	2	0	5	1	2	0	7	0	8	0	6	0	5	0	37
CMC	1	0	2	1	1	0	5	0	4	0	3	0	0	0	3	0	20
AA	0	0	0	0	2	0	2	0	5	0	5	0	2	0	8	0	24
Total EK	2	0	5	1	11	1	13	0	25	0	19	1	20	0	23	1	122
**ES**																	0
HVA	2	0	2	0	7	0	5	1	5	1	2	0	2	0	4	0	31
SCNA	3	0	2	1	3	1	5	0	4	0	0	1	2	0	3	0	25
CMC	1	0	1	0	5	0	4	0	5	0	1	0	6	0	6	0	29
AA	2	0	6	0	4	0	4	0	5	0	4	0	7	0	3	0	35
Total ES	8	0	11	1	19	1	18	1	19	1	7	1	17	0	16	0	120
**EW**																	0
HVA	5	0	0	0	6	0	3	0	3	0	3	0	3	0	3	0	26
SCNA	4	0	0	1	3	1	2	1	3	0	2	0	1	0	5	1	24
CMC	3	0	0	0	3	2	2	0	3	0	2	1	2	0	2	0	20
AA	4	0	1	0	8	1	2	0	2	0	2	0	1	0	11	0	32
Total EW	16	0	1	1	20	4	9	1	11	0	9	1	7	0	21	1	102

Medium/plant	Total number in each week																
	1	1	2	2	3	3	4	4	5	5	6	6	7	7	8	8	Total
	ST	NST	ST	NST	ST	NST	ST	NST	ST	NST	ST	NST	ST	NST	ST	NST	
HVA	10	0	5	0	32	0	22	1	24	1	17	1	30	0	25	1	169
SCNA	12	0	6	3	18	4	17	1	26	0	20	1	12	0	19	1	140
CMC	8	0	6	1	18	2	19	0	16	0	12	1	17	0	19	0	119
AA	10	0	11	0	19	1	14	0	24	0	16	0	17	0	31	0	143
Total	40	0	28	4	87	7	72	2	90	1	65	3	76	0	94	2	
	40		32		94		74		91		68		76		96		571

ST, *Streptomyces*; NST, non-*Streptomyces* genera. HVA, humic acid vitamin B agar; SCNA, starch casein nitrate agar; CMC, VL70 gellan gum with carboxymethyl cellulose; AA, VL70 gellan gum with amino acid mixture. EB, EC, EK, ES, and EW indicate *Eucalyptus camaldulensis* plant samples located in five different locations.

There were 25 isolates that were closely related to *Streptomyces rochei* NRRL B-2410^T^, sharing 16S rRNA gene similarity with this type strain between 97.8% and 100%. All plant samples contained isolates that were closely related to this type strain. These isolates were clustered with the closest type strain on the NJ tree with low bootstrap numbers. However, strains EBS5.1 and EBL7.9 were positioned in a different cluster with the type strain, *S. rochei* NRRL B-2410^T^, on the MP tree, while strain EBL7.9 was positioned in a different cluster with the closest type strain on the ML tree with low bootstrap numbers.

The next largest group comprised 13 isolates that shared the highest 16S rRNA gene similarity with *Streptomyces ardesiacus* NRRL B-1773^T^, between 98.8% and 99.9%. The members of this group were divergent. Ten isolates were grouped in the same cluster with this closest type strain with low bootstrap numbers. However, two isolates, ESR5.15 and EKR1.2, were positioned in the same cluster with *Streptomyces coelicoflavus* NBRC 15399^T^ on the NJ tree. Strain ESR5.15 is also positioned in the same cluster of *S. coelicoflavus* NBRC 15399^T^ on ML and MP trees with low bootstrap numbers (**Supplementary Figures S2**, **S3**). Isolate EWR1.23 was positioned in the same cluster of type strain, *Streptomyces albogriseolus* NRRL B-1305^T^, on NJ, ML, and MP trees.

Ten isolates shared the highest 16S rRNA gene similarity with the type strain, *Streptomyces thermoviolaceus* subsp. *apingens* DSM 41392^T^, between 98.1% and 99.2%. Nine isolates were grouped together and positioned in the same cluster with the type strain with low bootstrap numbers on NJ, ML, and MP trees. One isolate, EKL1.1, formed a separate clade with these nine isolates, but it was positioned close to the closest type strain on all phylogenetic trees. There were seven isolates that were closely related to *Streptomyces thermocarboxydus* DSM 44293^T^, sharing 16S rRNA gene similarity between 99.3% and 99.8%. These isolates were clustered together with low bootstrap numbers on NJ, ML, and MP trees. They were isolated from EC, ES, and EK samples.

There were 6, 5, 5, 4, and 3 isolates that were closely related to *Streptomyces violaceorectus* NBRC 13102^T^, *Streptomyces jiujiangensis* JXJ 0074^T^, *Streptomyces coelicoflavus* NBRC 15399^T^, *Streptomyces chromofuscus* NBRC 12851^T^, and *Streptomyces albogriseolus* NRRL B-1305^T^, respectively. The six isolates, closely related to *S. violaceorectus* NBRC 13102^T^, were positioned in the same cluster as the type strains on NJ trees, which have high bootstrap numbers. However, isolate ESR5.40 was located in a separate phylogenetic tree, distant from the other isolates on both the ML and MP trees. Five isolates, which were closely related to *S. jiujiangensis* JXJ 0074^T^, were divergent. Four isolates were positioned in the same cluster and close to the type strain with high bootstrap numbers on the NJ and ML trees but showed low bootstrap numbers on the MP tree. One isolate, EB1.5, was positioned in a different cluster with the type strains, and the other four isolates were on NJ, ML, and MP trees.

Five isolates, which were closely related to *S. coelicoflavus* NBRC 15399^T^, were positioned in the same cluster with the type strain on NJ, ML, and MP trees with high bootstrap numbers. They were closely related to members of the *S. ardesiacus* group. Three isolates, which were closely related to *S. albogriseolus* NRRL B-1305^T^, were positioned in the same cluster of this type strain on NJ, ML, and MP trees with low bootstrap numbers.

There were two isolates; each belonged to *Streptomyces spiralis* NBRC 14215^T^ and *Streptomyces roietensis* WES2^T^. The position of these isolates was close to their type strains on NJ, ML, and MP trees with high bootstrap support. Two isolates each were closely related to *Streptomyces yeochonensis* CN732^T^ and *Streptomyces nigra* 452^T^, but they were positioned in the different cluster of these closest type strains on NJ, ML, and MP trees with low bootstrap numbers. *S. yeochonensis* CN732^T^ was previously reclassified as the genus *Actinacidiphila*, but it was a heterotypic synonym of *Streptomyces* (Madhaiyan et al., 2022).

There were fifteen isolates of *Streptomyces*, which were a unique species isolated from each plant sample. All of them are positioned close to their closest type strain on the NJ tree with high bootstrap numbers. Only strain ECL5.16 was positioned in the different cluster with its closest type strain, *Streptomyces althioticus* NBRC 12740^T^, on the ML and MP trees with low bootstrap numbers. Two isolates of the ES sample belonged to the genus *Peterkaempfera*, which are closely related to the closest type strain*, Peterkaempfera griseoplana* NBRC 12779^T^, and formed the same cluster with this type strain with high bootstrap numbers on NJ, ML, and MP trees. *Peterkaempfera* was previously identified as the genus *Streptomyces* and later reclassified as the genus *Peterkaempfera* (Madhaiyan et al., 2022).

### 16S rRNA gene and phylogenetic analysis of non-*Streptomyces* strains

**Figure 3:**
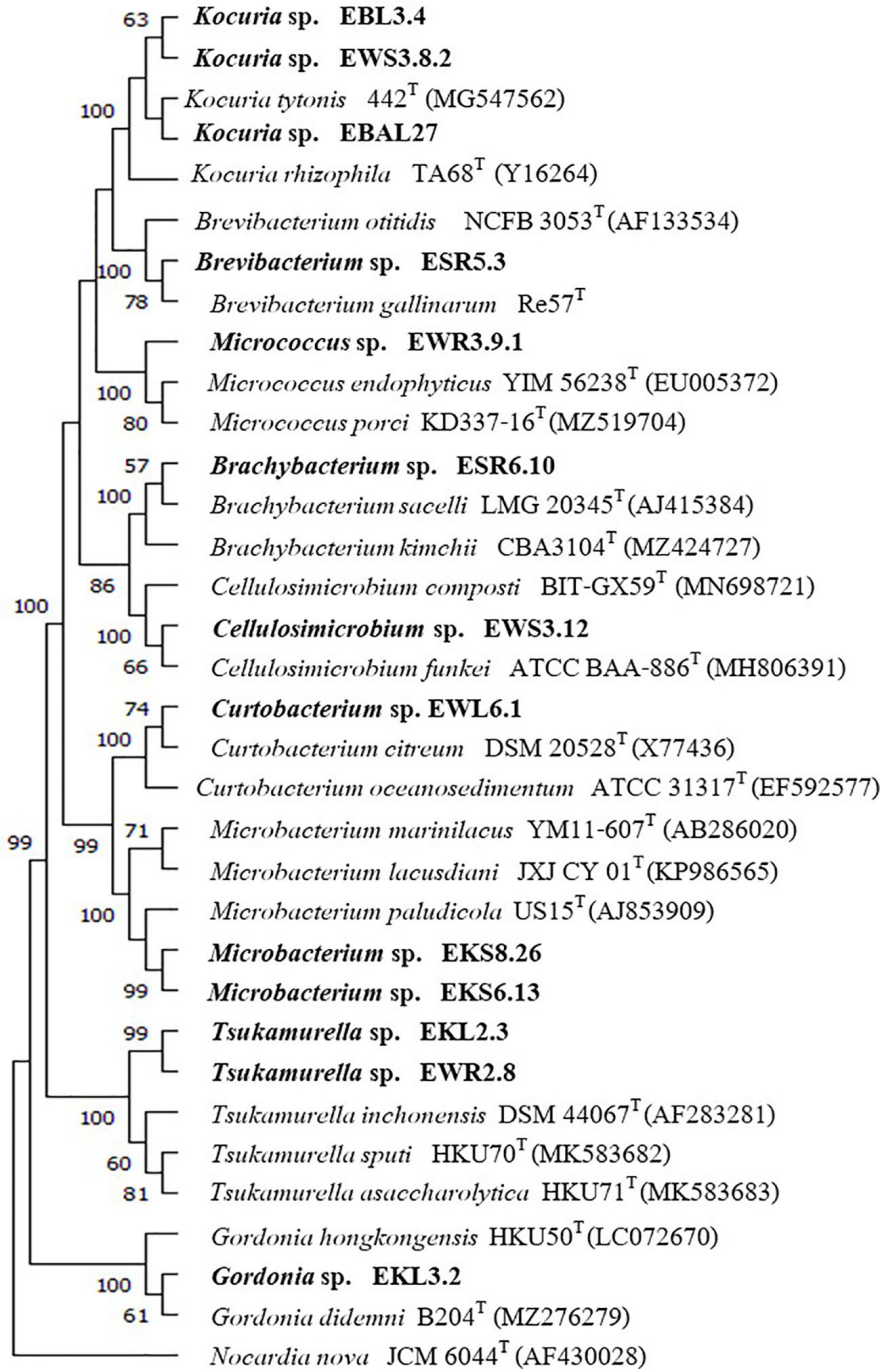
Neighbor-joining phylogenetic tree is based on the 16S rRNA gene sequences of 13 strains from 9 genera and their closely related members, with *Nocardia nova* JCM6044^T^ serving as the out-group. Bootstrap values based on 1000 replicates are shown at the branch nodes.

We constructed 16S rRNA gene NJ, ML, and MP phylogenetic trees that included all strains from ten non-*Streptomyces* genera. The result showed that each isolate was in the same cluster with its closest type strain with high bootstrap support on NJ, ML, and MP trees (**Figures 3**; **Supplementary Figures S4**, **S5**). In addition, two isolates of *Kocuria* from different plant samples (EB and EW) were in the same clades. Similarly, two isolates of *Tsukamurella* from plants (EK and EW) were in the same clade with high bootstrap support on NJ, ML, and MP trees, but they are in a different clade with the closest type strain on the NJ tree.

### Identification of non-actinobacteria isolates

We identified non-actinobacteria isolates based on 16S rRNA gene sequence analysis. There were 10 genera obtained: *Aureimonas*, *Bacillus, Chryseobacterium, Deinococcus, Massilia, Methylobacterium, Pseudomonas, Serratia, Staphylococcus*, and *Stenotrophomonas.***Supplementary Table S1** displays the details of the closest match for each isolate. The EK sample, which was grown in high-salinity soil and extremely acidic soil, comprised the most different genera of non-actinobacteria, at 8 genera: *Bacillus, Chryseobacterium, Deinococcus, Methylobacterium, Pseudomonas, Seratia, Staphylococcus*, and *Stenotrophomonas.* The EW sample was the second-best plant, which comprised five different genera, while the ES and EB samples contained four and three different genera, respectively. The non-actinobacteria strain was not isolated from the EC sample. EK and EW samples contained a unique genus of *Stenotrophomonas* and *Aureimonas*, respectively.

The study reported isolating the *Aureimonas* strain C2P003 from the leaf of the *Fraxinus excelsior* tree. This strain may suppress the colonization of ash dieback caused by the invasive pathogen, *Hymenoscyphus fraxineus* (Becker et al., 2022). *Deinococcus* was isolated from EK and EW plants. It was reported that *Deinococcus* are known for their resistance to extreme stresses, including radiation, oxidative stress, desiccation, and high temperature (Gerber et al., 2015). It was correlated from this study that the EK plant grown in high-salt soil yielded extremophile bacteria.

### Biodiversity of endophytic actinobacteria isolated from each medium

HVA was the best medium for yielding endophytic actinobacteria at 170 isolates, while SCNA and AA media yielded similar numbers at 140 and 143 isolates, respectively. CMC medium yielded the lowest numbers of endophytic actinobacteria at 118 isolates (**Table 1**). The majority of *Streptomyce*s isolates emerged from plant tissues in weeks 3, 5, and 8 at 87, 90, and 94 isolates, respectively. Most of the non*-Streptomyces* isolates emerged at week 3 at seven isolates.

HVA gave the highest numbers of *Streptomyces* at weeks 3, 4, and 7. SCNA gave the highest numbers of *Streptomyces* at weeks 1, 5, and 6, while AA medium had the highest numbers at weeks 2 and 8. SCNA was the best medium to yield the highest numbers of non-*Streptomyces* at weeks 2 and 3 (**Table 3**).

**Figure 4:**
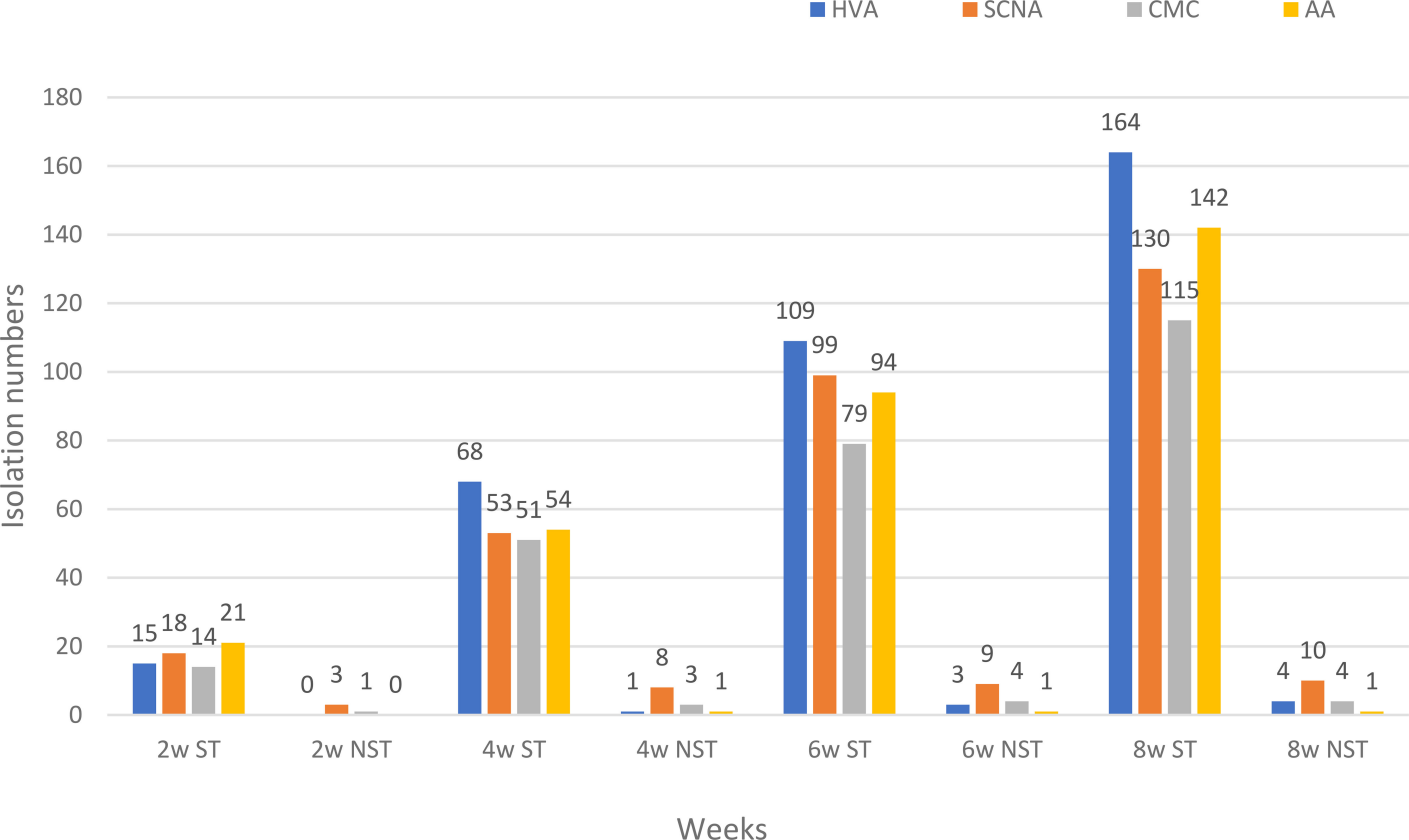
The total numbers of endophytic actinobacteria isolated from each plant sample at weeks 2, 4, 6, and 8 on four different media. HVA, humic acid vitamin B agar; SCNA, starch casein nitrate agar; CMC, VL70 gellan gum with carboxymethyl cellulose; AA, VL70 gellan gum with amino acid mixture; w, week; ST, genus *Streptomyces*; NST, Non-*Streptomyces* genera.

**Figure 4** shows the total numbers of isolates at weeks 2, 4, 6, and 8. In the first two weeks, AA medium was the best to give the highest numbers of *Streptomyces*. HVA gave the highest numbers of total *Streptomyces* isolates at weeks 4, 6, and 8 (N=68, 109, and 164). SCNA was the best medium to give the total numbers of non-*Streptomyces* isolates at weeks 2, 4, 6, and 8 (N=3, 8, 9, and 10). HVA gave the highest numbers of *Streptomyces* for the EK plant, which had a soil sample that was strongly saline and extremely acidic. Soil samples from the EB, EC, ES, and EW plants were moderately saline, with the highest numbers of isolates obtained from HVA for EB and EC, and from AA for ES and EW (**Table 3**).

The influence of media on the biodiversity of unique species of *Streptomyces* and non-*Streptomyces* isolates was studied. The result indicated that SCNA was the best to yield seven unique species of *Streptomyces*, followed by HVA (n=4), AA (n=3), and CMC (n=1) (**Table 4**). Furthermore, SCNA was the best medium to gain the highest number of non-*Streptomyces* at 10, followed by HVA (n=4), CMC (n=4), and AA (n=1) (**Table 4**). SCNA also gave the highest numbers of different genera of non-*Streptomyces* at seven, in which *Kocuria, Cellulosimicrobium, Tsukamurella, Micrococcus, Peterkaempfera*, and *Brachybacterium* are rare genera. HVA and AA media gave only 2 and 1 genera of non-*Streptomyces* isolates, respectively. Although CMC gave the lowest number of unique species of *Streptomyces*, it gave one unique isolate of *Curtobacterium*, which was a rare genus. Currently, there are only thirteen validly published species of *Curtobacterium* (Parte et al., 2020) (accessed July 10, 2025).

**Table 4:** Numbers of unique species of *Streptomyces* and all non-*Streptomyces* isolates isolated from each part of plants and each isolation medium.

Genus	Total	Part of plants			Isolation media			
		L	S	R	HVA	SCNA	CMC	AA
Unique species of *Streptomyces*	15	2	5	8	4	7	1	3
Non streptomycetes								
*Kocuria*	3	1	1	0	0	2	1	0
*Cellulosimicrobium*	3	0	2	1	0	2	1	0
*Tsukamurella*	2	1	0	1	0	1	1	0
*Microbacterium*	2	0	2	0	2	0	0	0
*Micrococcus*	2	1	0	1	0	1	0	1
*Peterkaempfera*	2	0	2	0	0	2	0	0
*Brevibacterium*	2	1	0	1	2	0	0	0
*Brachybacterium*	1	0	0	1	0	1	0	0
*Curtobacterium*	1	1	0	0	0	0	1	0
*Gordonia*	1	1	0	0	0	1	0	0
Total number	19	6	7	5	4	10	4	1

HVA, humic acid vitamin B agar; SCNA, starch casein nitrate agar; CMC, VL70 gellan gum with carboxymethyl cellulose; AA, VL70 gellan gum with amino acid mixture. L, leaf; S, twig; R, root.

Overall, for this study, HVA and SCNA were suitable media to isolate the highest number of *Streptomyces* and non*-Streptomyces* isolates, respectively. AA medium was the second one, which gave the highest number of *Streptomyces* isolates. Moreover, SCNA was the best media to yield the different genera of actinobacteria, including different species of *Streptomyces*. In addition, CMC media also gave the unique member of a rare genus. Therefore, the variety of isolation media can influence both numbers of isolates and numbers of different genera, including rare genera. We suggested that HVA and SCNA were the first choices to isolate endophytic actinobacteria from plants grown in saline soil. Also, CMC and AA media should be included as isolation media to enhance the number of rare genera and substantial biodiversity of *Streptomyces* species.

According to Qin et al. (2011) and Kaewkla and Franco (2013), isolation media have influenced the biodiversity of endophytic actinobacteria, including high numbers of unique species and rare genera. There were many reports showing that HVA was the best medium for isolating actinobacteria (Kaewkla and Franco, 2013; Himaman et al., 2016; Vo et al., 2021). Sardi et al. (1992) used starch casein medium to isolate endophytic actinobacteria from the roots of 28 plant species and obtained a good number of non-*Streptomyces* strains.

### Biodiversity of endophytic actinobacteria isolated from each plant sample

Plant samples in this study have influenced the biodiversity of both *Streptomyces* and non-*Streptomyces* isolates. EB gave the highest number of *Streptomyces* isolates at 123, with the rest from EK, ES, EC, and EW at 118, 115, 102, and 94 isolates, respectively. Although the EB sample comprised the highest numbers of *Streptomyces*, the biodiversity of *Streptomyces* species groups was the lowest at 4 groups. The EC sample contained the highest number of *Streptomyces* species groups at 12 groups, while the ES, EK, and EW samples contained 10, 9, and 9 *Streptomyces* species groups, respectively. Moreover, EC and EK samples comprised the highest numbers of unique *Streptomyces* species groups at 5, while the EW sample comprised unique species groups at 4. ES and EB samples comprised 3 and 2 unique *Streptomyces* species groups, respectively (**Table 5**).

**Table 5:** *Streptomyces* species groups based on 16S rRNA gene similarity and numbers of non-*Streptomyces* from each plant sample.

Genus/ the closest type strains		Plant samples				
		EB	EC	EK	ES	EW
*Streptomyces*						
*Streptomyces rochei* NRRL B-2410^T^		Y	Y	Y	Y	Y
*Streptomyces ardesiacus* NRRL B-1773^T^			Y	Y	Y	Y
*Streptomyces thermoviolaceus* subsp*. apingens* DSM 41392^T^			Y	Y	Y	Y
*Streptomyces thermocarboxydus* DSM 44293^T^			Y	Y	Y	
*Streptomyces coelicoflavus* NBRC 15399^T^			Y		Y	Y
*Streptomyces roietensis* WES2^T^			Y			Y
*Streptomyces jiujiangensis* JXJ 0074^T^		Y			Y	
*Streptomyces spiralis* NBRC 14215^T^			Y		Y	
*Streptomyces nigra* 452^T^			Y			
*Streptomyces althioticus* NRRL B-3981^T^			Y			
*Streptomyces hyderabadensis* OU-40^T^			Y			
*Streptomyces drozdowiczii* NBRC 101007^T^			Y			
*Streptomyces echinatus* DSM 40013^T^			Y			
*Streptomyces viridochromogenes* NBRC 3113^T^		Y				
*Streptomyces shenzhenensis* 172115^T^		Y				
*Streptomyces chiangmaiensis* TA4-1^T^				Y		
*Streptomyces lannensis* TA4-8^T^				Y		
*Streptomyces albus* NBRC 13014^T^				Y		
*Streptomyces pratensis* ch24^T^				Y		
*Streptomyces ginkgonis* KM-1-2^T^				Y		
*Streptomyces violaceorectus* NBRC 13102^T^					Y	
*Streptomyces chromofuscus* NBRC 12851^T^					Y	
*Streptomyces yeochonensis* CN732^T^					Y	
*Streptomyces puniceus* NBRC 12811^T^						Y
*Streptomyces albogriseolus* NRRL B-1305^T^						Y
*Streptomyces chartreusis* NBRC 12753^T^						Y
*Streptomyces griseorubiginosus* DSM 40469^T^						Y
Number of different groups		4	12	9	10	9
Number of unique groups		2	5	5	3	4
Non-*Streptomyces* genera	Total number	EB	EC	EK	ES	EW
*Gordonia*	1			1		
*Microbacterium*	2			2		
*Tsukamurella*	2			1		1
*Kocuria*	3	2				1
*Micrococcus*	2					2
*Cellulosimicrobium*	3					3
*Curtobacterium*	1					1
*Peterkaempfera*	2				2	
*Brevibacterium*	2				2	
*Brachybacterium*	1				1	
Number of different genera		1	0	3	3	5
Total number	19	2	0	4	5	8

Y; Isolates from each plant sample were classified as this species group. EB, EC, EK, ES, and EW indicate *Eucalyptus camaldulensis* plant samples located in five different locations.

Endophytic actinobacteria from the EK sample that were grown in highly salty soil at 12 dS/m and low pH at 3.9 may have adapted to survive in stressful conditions. Then, it would contain a greater biodiversity of actinobacteria than other moderately saline areas. A study by Himaman et al. (2016) obtained actinomycetes from the roots (23 samples) and rhizosphere soil (27 samples) of healthy *Eucalyptus* (*E. camaldulensis*) trees in different provinces of Thailand. There were 439 isolates that belonged to the genus *Streptomyces*, while 38 isolates were non-*Streptomyces* in nine different genera. Kaewkla and Franco (2013) isolated endophytic actinobacteria from surface-sterilized plant tissues of *Eucalyptus microcarpa* (grey box) and *Eucalyptus camaldulensis* (red gum) by using low-nutrient agar and long incubation of isolation plates. Although these two plant samples were the same genus, there were different numbers of actinobacterial isolates from gray box and red gum: 191 and 39 isolates, respectively. The grey box comprised *Streptomyces* (N=122) and non-*Streptomyces* (N=69) in nine different genera, while the red gum comprised *Streptomyces* (N=37) and non-Streptomyces (N=2) in two different genera.

There were nineteen non-*Streptomyces* isolates obtained in ten genera. The EW sample gave the highest number of non-*Streptomyces* at eight isolates, and the EW sample also gave the highest number of different genera at 5. There were 5, 4, and 2 isolates from ES, EK, and EB samples, respectively. ES, EK, and EB samples gave different numbers of genera at 3, 3, and 1, respectively. The EC sample, which comprised the highest numbers of different group species of *Streptomyces*, did not contain non-*Streptomyces* isolates. The ES plant sample was a valuable source because it yielded two rare genera: *Peterkaempfera* and *Brachybacterium.* These genera had only two and thirty-one valid published species at the time of this writing (Parte et al., 2020) (accessed July 10, 2025). EW and EK plant samples were also prospective sources to yield the rare genus *Tsukamurella*, which comprises 22 validly published species. We isolated *Cellulosimicrobium*, a rare genus with only 10 validly published species, exclusively from the EW plant. This suggests that the EW plant may harbor unique microbial diversity that could be crucial for further taxonomic studies. Additionally, the presence of these rare genera highlights the potential ecological significance of the EW plant’s environment, warranting further investigation into its role in supporting microbial life.

**Figure 5:**
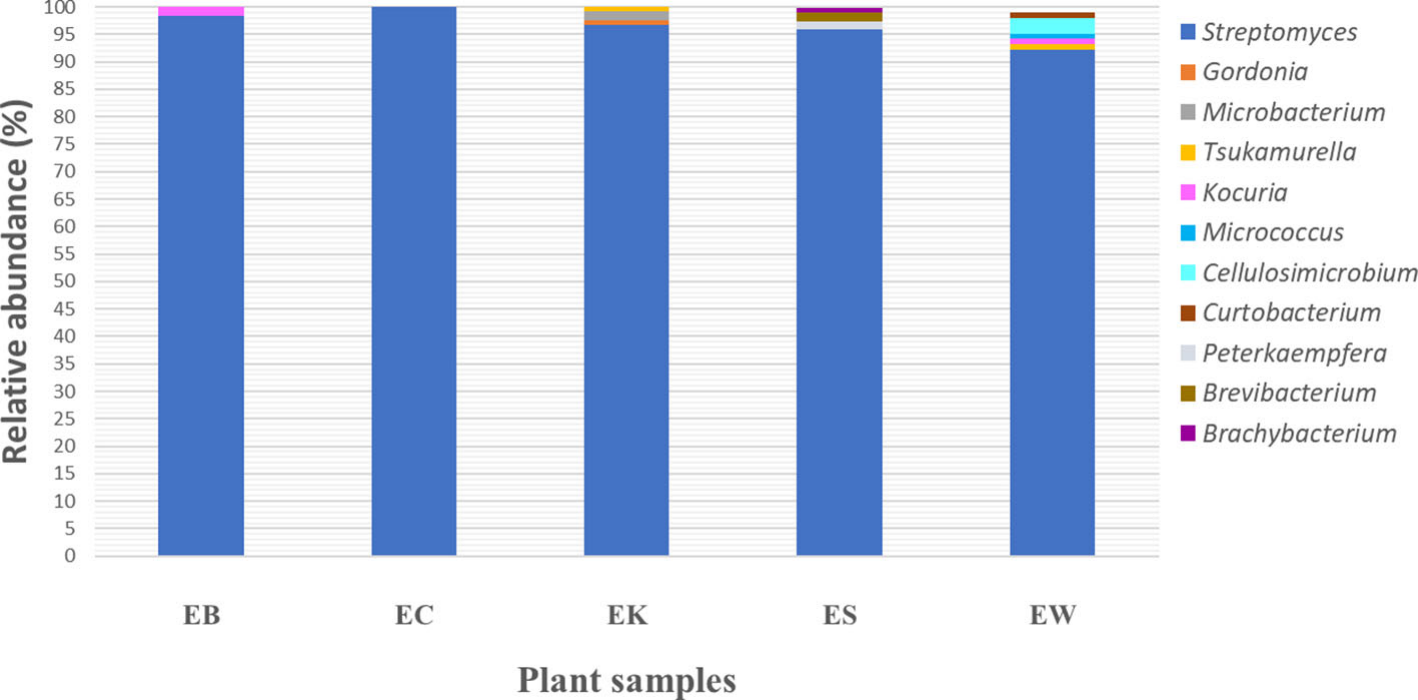
Bacterial community relative abundance analysis at the genus level from each plant sample. Different colors indicate eleven different genera. EB, EC, EK, ES, and EW indicate samples of the *Eucalyptus camaldulensis* plant from five different locations.

The relative genus abundance of endophytic actinobacteria from five plant samples revealed that *Streptomyces* was the predominant genus discovered from all plant samples, which ranged from 92.2 to 100% (**Figure 5**). Moreover, non-*Streptomyces* genera in the order *Micrococcales* were a predominant group recovered from four plant samples: EB, EK, ES, and EW. This group includes seven genera: *Brevibacterium, Brachybacterium, Cellulosimicrobium, Curtobacterium, Kocuria, Microbacterium*, and *Micrococcus*. However, the relative genus abundance of these genera in each plant sample was very low, between 1 and 2%. Moreover, the members of the order *Mycobacteriales*, comprising two genera, *Gordonia* and *Tsukamurella*, were found in low relative abundance in EK and EW plants (1-2%). The members of the genus *Peterkaempfera* were discovered only within the tissues of the ES plant sample with low relative abundance at 1%. This genus was a rare one and belonged to the family *Streptomycetaceae*, which is the same family as the genus *Streptomyces.*

The analysis of plant parts comprising unique species of *Streptomyces* and non-*Streptomyces* genera is presented in **Table 4**. Root tissues gave the highest numbers of unique species of *Streptomyces* at eight different species. Stem tissues gave the highest numbers of non-*Streptomyces* at 7, followed by leaf and root tissues at 6 and 5 isolates, respectively.

There were some reports of non-*Streptomyces* genera that exhibited plant-growth-promoting capabilities. These findings suggest that the diversity of microbial sources for potential plant growth promoters may be broader than previously thought, opening new avenues for an agricultural area. *Microbacterium* was isolated from *Limonium sinense*, enhancing salinity tolerance in the plant (Qin et al., 2018). *Brachybacterium paraconglomeratum* strain SMR20 isolated from *Chlorophytum borivilianum* improved the salt tolerance and yield of the host plant (Barnawal et al., 2016). *Cellulosimicrobium* 60I1 was a promising strain for preparing a bioinoculant to promote the growth of a pepper plant (Lobato-Ureche et al., 2023). *Curtobacterium albidum* strain SRV4 had the ability to fix nitrogen (N_2_) and produce exopolysaccharide (EPS), hydrogen cyanide (HCN), IAA, and ACC deaminase. Moreover, *Curtobacterium* strain SRV4 promotes paddy plants by enhancing plant growth parameters, photosynthetic pigment efficiency, membrane stabilization index, and proline content (Vimal et al., 2019). *Kocuria* strain ST19, which was isolated from halophytes, could show efficiency to mitigate salt stress conditions in tomato plants (Dif et al., 2021). In addition, *Brevibacterium sediminis* strain IBGE3C, which was isolated from a saline area, promoted the seedling growth of rice (Mahmud-Ur-Rahman et al., 2022). Therefore, non-*Streptomyces* strains from this study might have the ability to promote eucalyptus growth in salinity conditions, but it requires further study to prove their properties.

### Antifungal activity

Two hundred and seventy-three isolates of endophytic actinobacteria were tested for antifungal activity against two eucalyptus pathogens, *P. eucalypti* LS6 and *Cladosporium* sp. LB1. There were more isolates showed good activity against *P. eucalypti* LS6 than *Cladosporium* sp. LB1. There were 61 (22.3%) and 104 (38.1%) isolates that showed good and strong inhibition against *P. eucalypti* LS6, while 35 (12.8%) and 5 (1.8%) isolates demonstrated good and strong inhibition against *Cladosporium* sp. LB1 (**Table 6**). There were EB isolates (n=33) that showed strong activity against *P. eucalypti* LS6 (**Supplementary Figure S6**), while there were ES isolates (n=3) that showed strong inhibition against *Cladosporium* sp. LB1 (**Supplementary Figure S7**). We isolated the fungal strains LS6 and LB1 from eucalyptus leaves with leaf spot and leaf blight symptoms, respectively. We tested both strains for disease symptoms on the surface-sterilized eucalyptus leaves using the detached leaf assay. The result showed that *P*. *eucalypti* LS6 and *Cladosporium* sp. LB1 caused severe and moderate lesions on the leaves, respectively (data not shown). *Pseudoplagiostoma eucalypti* was first named *Cryptosporiopsis eucalypti* by Sankaran et al. (1995), but it was later reclassified as *Pseudoplagiostoma eucalypti.* This fungus causes severe leaf spots on eucalyptus trees and is common in both tropical and temperate countries (Cheewangkoon et al., 2010). Lueangpraplut et al. (2013) reported that *P. eucalypti* caused leaf spot and shoot blight diseases on eucalyptus in Thailand. Zauza et al. (2023) reported the severe disease and dieback of *Eucalyptus* spp. in Brazil, caused by *P. eucalypti*. It was recently reported that this fungus showed the first serious outbreak of eucalyptus disease after more than two decades in northern India (Negi et al., 2024). *Cladosporium* spp. are endophytic, or dormant, fungi that have been found to spread disease on dead parts of many different host plants (Bensch et al., 2010). *Eucalyptus ficifolia* was infected by *Cladosporium* sp. in New Zealand (Farm Forestry New Zealand, 1992). All these actinobacterial isolates, which showed good and strong inhibition against these fungal pathogens, belonged to the genus *Streptomyces*. This was in line with other studies that found most isolates of the genus *Streptomyces* could inhibit fungi (Himaman et al., 2016; Pushpalatha et al., 2023; Shahid et al., 2021). It was reported that 273 isolates of endophytic actinobacteria from *Eucalyptus microcarpa* and *Eucalyptus camaldulensis* were tested for their antifungal activity against plant pathogens *Phytophthora palmivora* and *Fusarium oxysporum* (Kaewkla, 2009). The result showed that most strains belonging to the genus *Streptomyces* had good activity inhibiting these two fungal pathogens *in vitro*. Himaman et al. (2016) studied endophytic actinobacteria from eucalyptus by testing them against three types of fungi: *P. eucalypti* (*Cryptosporiopsis eucalypti*)*, Cylindrocladium* sp., and *Teratosphaeria destructans.* As a result, 57%, 24.7%, and 50.5% of the isolates were able to inhibit these fungal pathogens, respectively, and most of these strains belonged to the genus *Streptomyces*.

**Table 6:** Numbers of endophytic actinobacteria inhibited two fungal pathogens.

Isolates	Number of isolates inhibited fungal pathogens										
	*Pseudoplagiostroma eucalypti* LS6					*Cladosporium* sp. LB1					Total
	NI	1+	2+	3+	4+	NI	1+	2+	3+	4+	
EB	0	5	12	4	33	1	17	28	7	1	54
EC	0	6	14	18	14	8	28	16	0	0	52
EK	1	14	18	6	18	1	31	18	6	1	57
ES	14	5	11	21	24	26	13	18	15	3	75
EW	0	3	5	12	15	5	12	11	7	0	35
Total	15 (5.5%)	33 (12.1%)	60 (22%)	61 (22.3%)	104 (38.1%)	41 (15%)	101 (37%)	91 (33.3%)	35 (12.8%)	5 (1.8%)	273

NI; no inhibition, 1+; weak, 2+; moderate, 3+; good inhibition, 4+; strong inhibition.

### Plant growth promoting study and bacterial inhibition

For the PGP study *in vitro*, 154 isolates were chosen because they showed good or strong activity against at least one tested fungus. They were also studied antibacterial activity against *R. solanacearum* TISTR 2069, which causes eucalyptus leaves to wilt. There were only two isolates (1.3%) from the EW sample showing good inhibition against this bacterium (**Supplementary Figure S8**), and they belonged to the genus *Streptomyces* (**Table 7**). It was reported that leaf wilt disease occurrence got 40% of the eucalyptus field, and destroyed plants exhibited reddening and wilting of the foliage, leaf drop, and branch dieback with wilting symptoms (Santiago et al., 2014). It was found that strains UFV-56 (*Bacillus thuringiensis*) and UFV-62 (*Bacillus cereus*), along with a commercial mixture of several rhizobacteria called Rizolyptus^®^, inhibit the bacterial wilt in eucalyptus trees (Santiago et al., 2015). *Ralstonia* wilt of chili pepper caused by *R. solanacearum* was inhibited by *Streptomyces philanthi* RL-1-178 (Boukaew et al., 2011).

**Table 7:** Numbers of endophytic actinobacteria inhibited a bacterial pathogen and presented plant growth promoting properties.

Isolates	Numbers of isolates inhibit *R. solanacearum* TISTR 2069				Number of isolates				Range of IAA production (ug/ml) and number#	Total
	NI	1+	2+	3+	N fixation	ACC deaminase	Hydrolyze CMC	Solubilize phosphate		
EB	22	0	1	0	0	0	17	6	6.9-33.2 (23)	23
EC	26	0	0	0	7	12	26	0	10.7-32.7 (26)	26
EK	16	6	2	0	0	4	18	8	13.1-31.6 (24)	24
ES	35	6	3	0	0	1	16	0	11.2 -48.2 (44)	44
EW	17	11	7	2	0	1	16	0	8.8 -38.6 (37)	37
Total	116 (75.3%)	23 (14.9%)	13 (8.4%)	2 (1.3%)	7 (4.5%)	18 (11.7%)	93 (60.4%)	14 (9.1%)	154 (100%)	154

NI, no inhibition; 1+, weak; 2+, moderate; 3+, good inhibition; N, nitrogen; ACC, 1-aminocyclopropane-1-carboxylic acid; CMC, carboxymethyl cellulose; IAA, indole-3-acetic acid.#, Number of positive result are in bracket.

**Figure 6:**
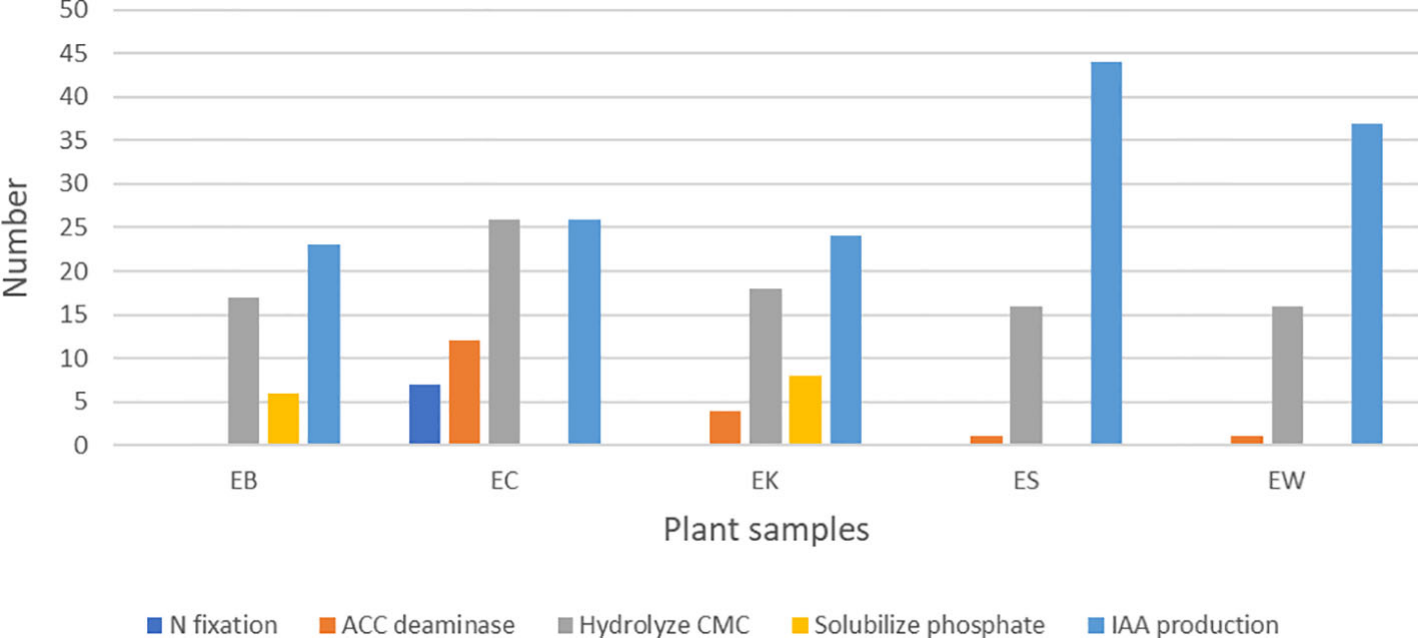
Numbers of endophytic actinobacteria present plant growth promoting traits *in vitro* from each plant sample. EB, EC, EK, ES, and EW indicate *Eucalyptus camaldulensis* plant samples located in different locations. N, nitrogen; ACC, 1-aminocyclopropane-1-carboxylic acid; CMC, carboxymethyl cellulose; IAA, indole-3-acetic acid.

The result showed that all these endophytic actinobacteria produced IAA which ranged from 6.9 to 48.2 ug/ml. ES isolate produced the highest IAA at 48.2 ug/ml. There were 93 strains (60.4%) that could hydrolyze CMC, and all isolates from EC samples could hydrolyze CMC. There were 18 isolates that could produce ACC deaminase and 14 isolates that could dissolve phosphate (**Table 7**; **Figure 6**). Most isolates from the EC sample produced ACC deaminase (66.7%). Only isolates from EK and EB samples (8 and 6 isolates) could solubilize phosphate. Only isolates from the EC sample could fix nitrogen (n=7). The mechanisms of actinobacteria to support plant growth in salinity stress include phytohormone production, especially auxin. Production of 1-aminocyclopropane-1-carboxylate (ACC) deaminase reduce ethylene gas and accumulation of osmoprotectant compounds such as proline, glycine-betaine, and polyamine. Some strains accumulate exopolysaccharides (EPS) and production of anti-oxidative enzymes to reduce oxidation stress (Atouei et al., 2019; Feikema and Baker, 2011). Thus, selected strains of endophytic actinobacteria producing IAA and ACC deaminase may have the ability to promote plant growth in salinity conditions *in planta*. Himaman et al. (2016) studied endophytic actinobacteria from eucalyptus tissues and rhizosphere soil for siderophores and IAA production and phosphate solubilization. The result showed that the *Streptomyces* sp. strain EUSKR2S82 could strongly inhibit all tested fungi and displayed plant growth-promoting traits *in vitro* and *in planta*.

### Salt tolerance study

Based on the result of plant-growth-promoting properties, thirty-five potential strains of endophytic actinobacteria were selected to test on different sodium chloride concentrations up to 11% (w/v). Most isolates could grow well at 5% NaCl (w/v), while 6, 4, and 1 isolate could grow well at 7%, 9%, and 11%, respectively (**Supplementary Table S2**). Then, based on the PGP traits and ability of salt tolerance, the promising strains will be selected to test for PGP in planta in the further study.

### Seed germination test

**Figure 7:**
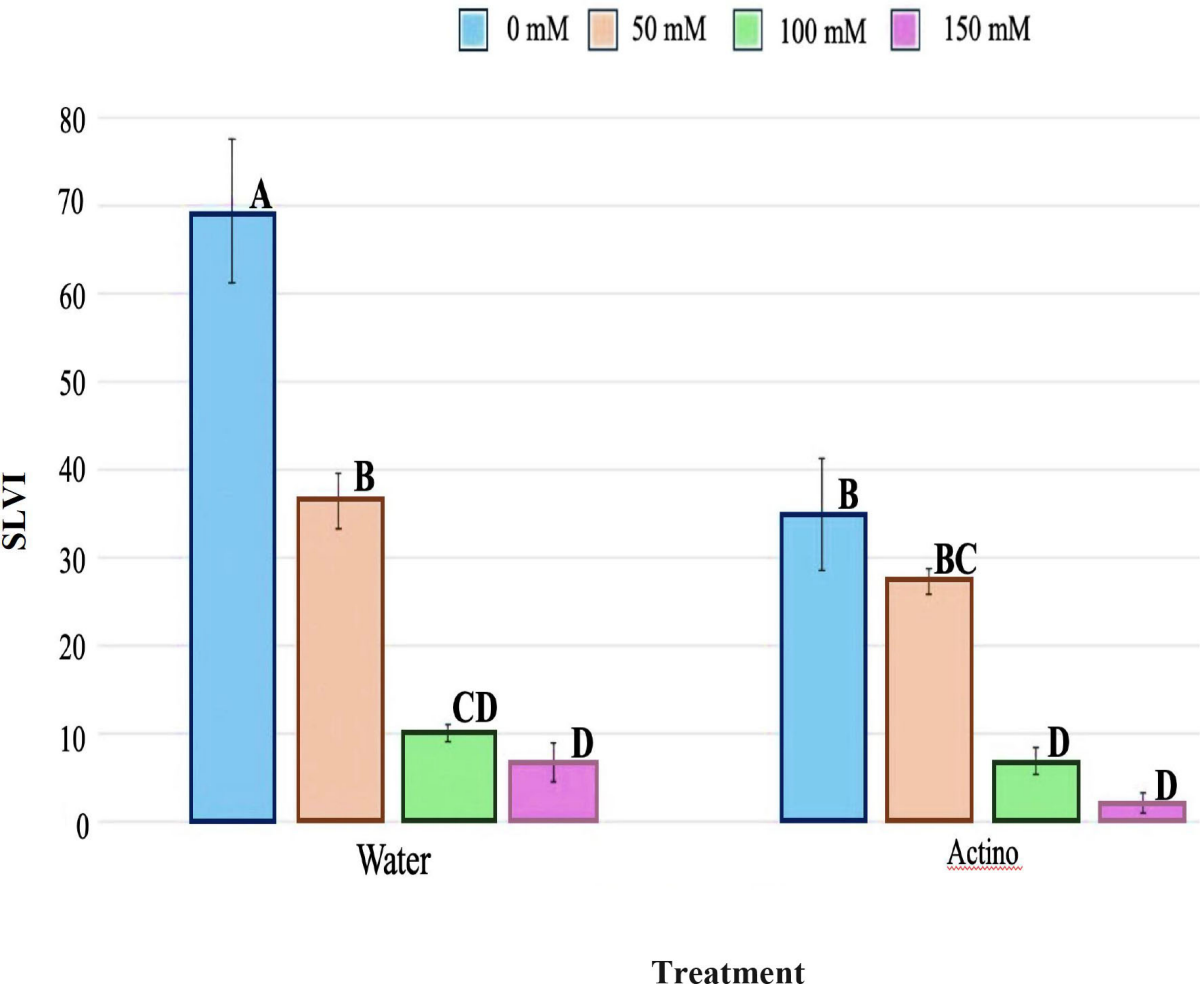
Effect of inoculation on seedling length vigor index (SLVI) on eucalyptus seedlings treated with strain EWL5.16 (Actino) or without strain EWL5.16 (Water) at 0, 50, 100, and 150 mM NaCl. Values represent the mean of five biological replicates per treatment, and bars indicate the standard error (SE). Means followed by the different letters for treatments are significantly different according to the Tukey Honestly Significant Difference (HSD) test (*p* < 0.05).

The test for germination potential (GP), germination rate (GR), and seedling length (SL) showed that there was no interaction between actinobacteria and salt (**Supplementary Figure S9**). The only parameter that had an interaction was the seedling length vigor index (SLVI) (**Table 8**). The treatment of *Streptomyces* strain EWL5.16 suppressed seed germination and seedling growth, as GR and SL were significantly lower than the control (water) (*P<*0.05), but it was not significant for GP. The concentration of salt between 100 mM and 150 mM did not significantly affect (*P*<0.05) GP, GR, and SLVI, while SL was significantly reduced (*P*<0.05) by these salt concentrations. However, treatment with strain EWL5.16 supported the SLVI by reducing the effect of salt at 50 mM compared with water (**Figure 7**). The SLVI of the treatment with strain EWL5.16 at 0 mM and 50 mM NaCl was not significantly different (*P*<0.05), while the SLVI of the control treatment at 50 mM was significantly lower than the SLVI at 0 mM NaCl (*P*<0.05).

**Table 8:** Effect of inoculation on seed germination test treated with *Streptomyces* sp.

Treatment	GP	GR	SL (cm)	SLVI
PGPB				
Water	19 ± 4.08	45.67 ± 5.03^a^	0.55 ± 0.069^a^	30.38 ± 6.14^a^
EWL5.16	11 ± 3.18	34.33 ± 5.13^b^	0.42 ± 0.052^b^	17.80 ± 3.49^b^
Sodium chloride				
0 mM	33.33 ± 5.62^a^	62 ± 5.26^a^	0.82 ± 0.073^a^	52.04 ± 7.48^a^
50 mM	20.67 ± 3.65^a^	54 ± 3.778^a^	0.59 ± 0.025^b^	31.74 ± 2.22^b^
100 mM	4 ± 1.09^b^	26.67 ± 4.22^b^	0.34 ± 0.035^c^	8.33 ± 0.98^c^
150 mM	2 ± 1.02^b^	17.34 ± 4.47^b^	0.19 ± 0.034^d^	4.26 ± 1.30^d^
Significance				
PGPB	ns	*	*	*
Salt	*	*	*	*
IA	NA	NA	NA	IA

PGPB, EWL5.16 or water in salt stress condition.

GP, germination potential; GR, germination rate; SL, seedling length; SLVI, Seedling length vigor index. * significant at *p*<0.05, ns; not significant, IA; interaction, NA; no interaction. Values represent the mean of 5 biological replicates per treatment, and ± indicate the standard error (SE). The means, followed by the different letters in the same column, are significantly different (*p* < 0.05). The General Linear Model (GLM) was employed to examine a statistically significant interaction effect, while Tukey’s Honestly Significant Difference (HSD) test served as a *post-hoc* analysis to assess significant differences among treatments.

### Plant growth promoting in planta

**Figure 8:**
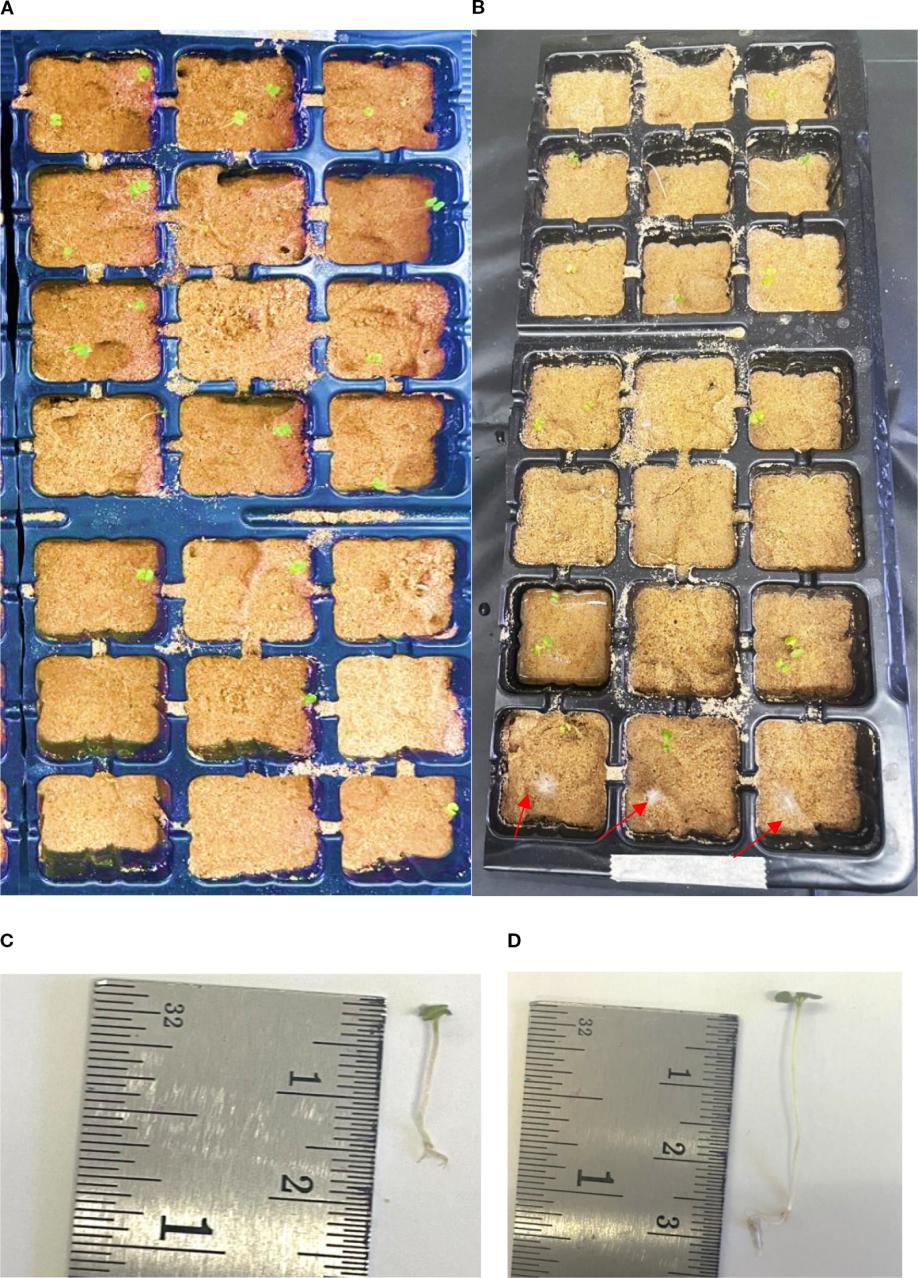
Plant growth-promoting test of endophytic actinobacteria on eucalyptus seedlings grown on sterilized sand for 14 days. **(A)** Plant growing tray with seedlings treated with strain EWL5.16. **(B)** Plant growing tray treated with water (red arrows: seed-borne fungi destroyed seedlings). **(C)** Eucalyptus seedling treated with water. **(D)** Eucalyptus seedling treated with strain EWL5.16.

The PGP study in planta showed similar results with the seed germination test. Seedlings of the *Streptomyces* EWL5.16 treatment had lower shoot length, root length, and seedling length than the control, but these growth parameters were not significantly different from the control (**Table 9**). These findings suggested that while the *Streptomyces* EWL5.16 treatment may not adversely affect seedling growth in a statistically significant manner, it could still influence the overall development patterns of the plants. Further investigation is needed to explore the underlying mechanisms and potential long-term effects of this treatment on plant growth. However, the fresh weight of seedlings in strain EWL5.16 treatment was significantly higher than the control (*P*<0.05) (**Table 9**). Moreover, seed-borne fungi, *Fusarium* sp., severely infected seedlings, and most plants died at day 7 after planting (**Figure 8**). The survival rate of seedlings in strain EWL5.16 treatment (36.1%) was higher than in the control treatment (27.4%). In this study, the *Streptomyces* strain EWL5.16 suppressed seed germination and seedling growth, which might involve bioherbicide or cellulose production to inhibit root cell walls. Moreover, IAA production of strain EWL5.16 may negatively impact plant growth. The further study by varying the lower spore inoculum for coating seeds might reveal if this strain can be a potential inoculum to promote eucalyptus seedlings in the salinity stress. According to Bo et al. (2019), the *Streptomyces* strain 329 showed the potential bioherbicidal efficacy by testing on grass and broadleaf weeds for phytotoxic activity. At pre-emergence application, the phytotoxic efficacy of strain 329 to *Digitaria sanguinalis* and *Sorghum bicolor* on seed germination was 90.4% and 81.3%, respectively. *Streptomyces* sp. KRA16–334 was grown on M3 medium, and its ability to kill 10 weeds was tested using the culture filtrate. The result showed that the 2-fold dilution applied to leaves resulted in complete control of nine weed species (Kim et al., 2024).

**Table 9:** Effect of inoculation on plant growth parameters and survival rate (%) of eucalyptus seedlings treated with water or strain EWL5.16.

Treatment	Growth				
	Shoot length (cm)	Root length (cm)	Seedling length (cm)	Fresh weight (mg)	Survival rate (%)
Water	1.716 ± 0.076	0.409 ± 0.03	2.125 ± 0.097	3.98 ± 0.2^a^	27.4
EWL5.16	1.552 ± 0.074	0.387 ± 0.031	1.939 ± 0.09	5.2 ± 0.3^b^	36.1

Values represent the mean of six biological replicates per treatment, and ± indicate the standard error (SE). Means followed by the different letters in the same column are significantly different (*p* < 0.05). The Mann-Whitney U test was used for statistical analysis.

### Genome comparison study

The genomes of *Streptomyces* strains EKR5.2 and ESS7.8 were compared with their closest type strains. Strain EKR5.2 had the highest dDDH, ANIb, and ANIm values at 96.2%, 99.3%, and 99.6% with *Streptomyces lannensis* JCM16578^T^. Strain ESS7.8 shared the highest dDDH, ANIb, and ANIm values at 94.2%, 99.0%, and 99.3% with *Streptomyces ardesiacus* NBRC 15402^T^. *Streptomyces* strains ECR2.10 and EWL5.1 were very close and had a 99.9% similarity in their 16S rRNA gene. The dDDH, ANIb, and ANIm values for these two strains were 99.9%, 99.9%, and 99.99%, respectively. The closest type strain, which shared the highest 16S rRNA gene similarity at 99.0% with these two strains, was *Streptomyces roietensis* WES2^T^. However, this type strain has not been sequenced in the genome. Therefore, there is no data on the genome comparison study between this type strain and strains ECR2.10 and EWL5.1.

*Micrococcus* strain EWR3.9.1 shared the highest dDDH, ANIb, and ANIm values with the type strain *Micrococcus luteus* ATCC 4698^T^ at 76.4%, 96.7%, and 97.4%. The species-level definition should have dDDH and ANI values lower than the threshold of 70% (Meier-Kolthoff et al., 2013) and 95-96% (Richter and Rosselló-Móra, 2009), respectively. Therefore, *Streptomyces* strains EKR5.2 and ESS7.8 and *Micrococcus* strain EWR3.9.1 belonged to the known species. *Streptomyces* strains ECR2.10 and EWL5.1 were the same species. However, to confirm their novelty, a genome comparison with the closest species, *S. roietensis* WES2^T^, was necessary.

**Figure 9:**
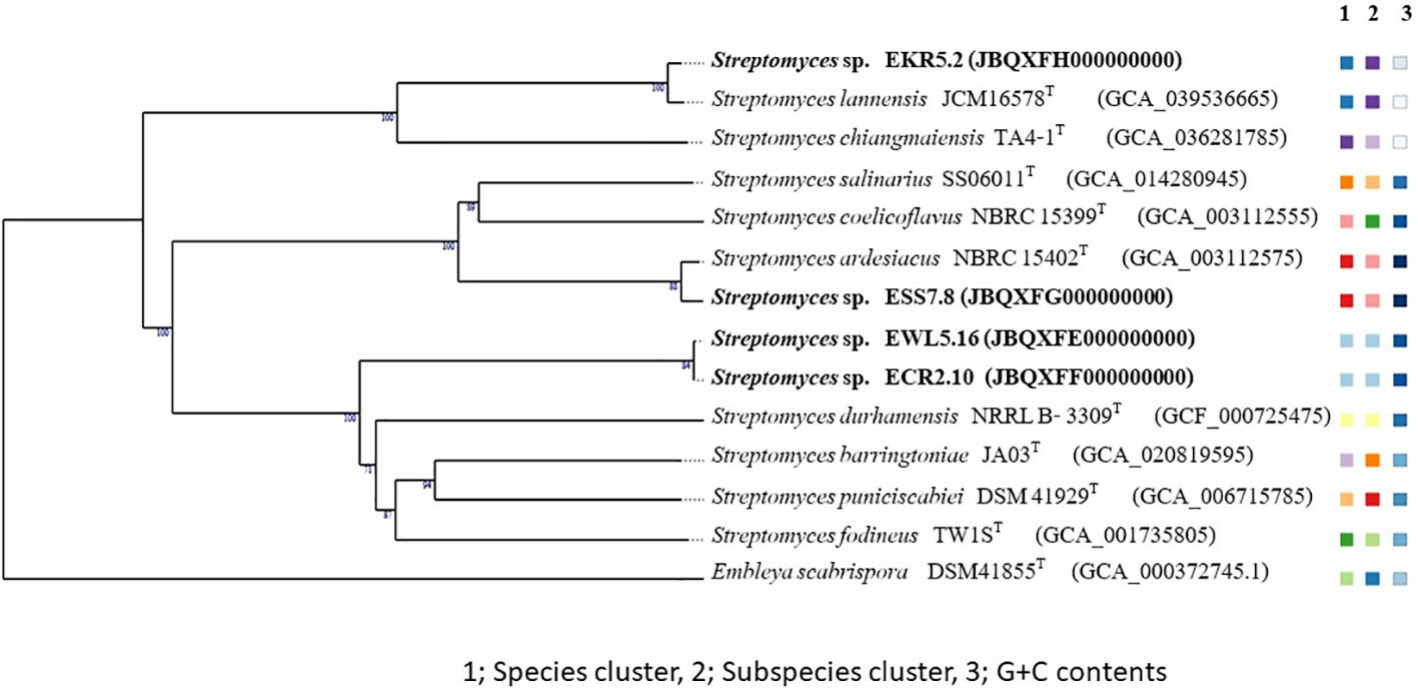
A phylogenomic tree based on the TYGS result shows the relationship between *Streptomyces* strains EKR5.2, ESS7.8, ECR2.10, and EWL5.16, as well as their closely related type strains. The tree was inferred with FastME 2.1.6.1 (Lefort et al., 2015) from GBDP distances calculated from genome sequences. The numbers above branches are GBDP pseudo-bootstrap support values > 60% from 100 replications, with an average branch support of 92.1%. The tree was rooted at the midpoint (Farris, 1972).

**Figure 10:**
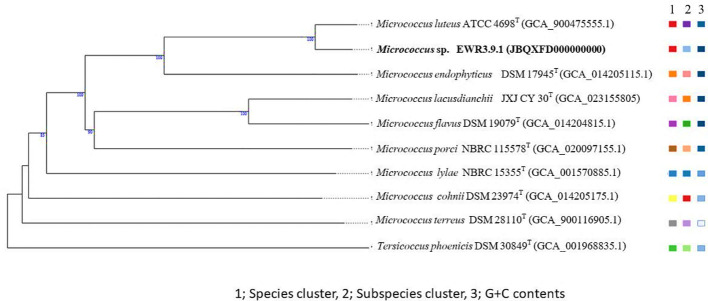
A phylogenomic tree based on the TYGS result shows the relationship between *Micrococcus* EWR3.9.1 and their closely related type strains. The tree was inferred with FastME 2.1.6.1 (Lefort et al., 2015) from GBDP distances calculated from genome sequences. The numbers above branches are GBDP pseudo-bootstrap support values > 60% from 100 replications, with an average branch support of 89.4%. The tree was rooted at the midpoint (Farris, 1972).

The TYGS phylogenomic tree of *Streptomyces* strains EKR5.2, ESS7.8, ECR2.10, and EWL5.1 also supports the dDDH, ANIb, and ANIm values between these strains and their closest type strains. Strains EKR5.2 and ESS7.8 were in the same clade with their closest type strains with high bootstrap support. Also, strains ECR2.10 and EWL5.1 were in the same clade and in the separated cluster with other type strains (**Figure 9**). The TYGS phylogenomic tree of *Micrococcus* strain EWR3.9.1 showed that it formed the same clade as the closest type strain, *M. luteus* ATCC 4698^T^, with the high bootstrap number (**Figure 10**).

The GenBank accession numbers for the genomes of *Streptomyces* strains EKR5.2, ESS7.8, ECR2.10, and EWL5.1, as well as *Micrococcus* strain EWR3.9.1, are JBQXFH000000000, JBQXFG000000000, JBQXFF000000000, JBQXFE000000000, and JBQXFD000000000, respectively.

### Biosynthetic Gene Clusters and gene prediction

All *Streptomyces* strains, EKR5.2, ESS7.8, ECR2.10, and EWL5.1, contain BGCs of hopene (53, 100, 92, 92%) and geosmin (100% for all), which are common compounds found in many strains of *Streptomyces* (**Supplementary Tables S3**–**S5**). Strains EKR5.2 and ESS7.8 comprise BGCs of spore pigment (66, 66%), while only strains EKR5.2 and ECR2.10 have BGCs of melanin compound (42, 71%). Strains EKR5.2, ESS7.8, ECR2.10, and EWL5.1 contain BGCs of informipeptin (42, 42, 100, 100%) and albaflavenone (100% for all), which is a tricyclic sesquiterpene antibiotic with antibacterial activity produced by *Streptomyces* (Moody et al., 2012). All four strains have BGCs of germicidin (100% for all), which is an auto-regulative suppressor of spore germination in the genus *Streptomyces* (Petersen et al., 1993). The genome of strain EKR5.2 comprises BGCs of the invaluable compound ϵ-Poly-L-lysine (100%), which is a microbial peptide that is used as an antimicrobial compound to preserve packaged food. It was reported that ϵ-PL is competitive to use widely worldwide because it shows broad antimicrobial activity against Gram-negative and Gram-positive bacteria, yeasts, and molds. However, the production of this compound is limited because the yield of the commercial strain was low (Ye et al., 2013). Therefore, strain EKR5.2 will be a beneficial strain to develop as a commercial strain in the future.

The genome of strain ESS7.8 comprises higher numbers of BGCs than the genomes of the other three strains. Of these BGCs, BGCs of thiazostatin, watasemycins A and B (100%), new antibiotics produced by *Streptomyces* sp. TP-A059, which showed antibacterial activity against Gram-staining positive and negative bacteria, and yeast are detected in the genome of strain ESS7.8 (Sasaki et al., 2002). The genome of strain ESS7.8 comprises BGCs of coelibactin (100%), a bacterial toxin that is produced by some strains of bacteria living in the human gut and induces prophage to be in the lytic development stage (Silpe et al., 2022). The genome of strain ESS7.8 contains BGCs of butyrolactol A (86%), which showed activity against fungal human pathogens, such as *Candida albicans* and *Trichophyton mentagrophytes* (Harunari et al., 2017). Moreover, the genome of strain ESS7.8 comprises a BGC of enterocin (95%). It was reported that the genome of *Streptomyces qinglanensis* 172205, isolated from mangroves, comprises BGCs of enterocin, a bacteriocin that can inhibit activity against β-amyloid protein (Aβ_1-42_) fibrillation and moderate cytotoxicity against HeLa and HepG2 (Xu et al., 2015). In addition, the genomes of strains ESS7.8 and ECR2.10 contain BGCs of alkylresorcinols (Ars) (100, 100%), which are polyphenolic compounds with the potential to be used as the regulation of host metabolism. The study showed that the intestinal microbiota could produce several Ars related to olivetol supplementation and microbiota metabolic activity. Moreover, Ars are potential quorum-sensing molecules, which support gut microbiota composition and host metabolism (Zabolotneva et al., 2023). Genomes of strains ESS7.8, ECR2.10, and EWL5.1 contain BGC of 1,3,6,8-tetrahydroxynaphthalene (THN) (100% for all), which is a precursor of melanin production. It was reported that THN derivative IBR33 produced by *Nocardia* sp. CS682 showed promising UV protection effects (Mishra et al., 2019).

*Streptomyces* strains ECR2.10 and EWL5.1 were close to each other (99.9% dDDH value). The BGCs of strains ECR2.10 and EWL5.1 were mostly the same, but the BGCs of melanin, methylated alkyl-resorcinol, and aurantimycin A were only found in strain ECR2.10. Many strains of *Streptomyces* produce Pentalenolactone, a sesquiterpenoid antibiotic that is detected in the genomes of strains ECR2.10 and EWL5.1 (58, 58%). Pentalenolactone inhibited both Gram-positive and Gram-negative bacteria, as well as pathogenic and saprophytic fungi (Takamatsu et al., 2011). The genome of strain ECR2.10 comprises the BGC of Aurantimycin (ATM), produced by *Streptomyces aurantiacus* JA 4570. ATM showed strong activity against some strains of Gram-positive bacteria and possessed cytotoxic activity against L-929 mouse fibroblast cells. The tandem overexpression of genes *artB* and *art*X increases the production of ATM about 2.5-fold in *S. aurantiacus* JA 4570 (Zhao et al., 2016). Genomes of strains ECR2.10 and EWL5.1 contain interesting BGCs of Cystargolides A and B (90, 90%), which are rarely reported. Cystargolides A and B were initially isolated from *Kitasatospora cystarginea* NRRL B16505 and showed activity to inhibit the human proteasome and the caseinolytic protease ClpP in the micromolar range (Beller et al., 2024). These findings highlight the diverse biosynthetic capabilities of the analyzed *Streptomyces* strains, suggesting their potential for producing a variety of bioactive compounds. Further investigation into the specific roles of these BGCs could lead to new discoveries in antibiotic development and natural product chemistry.

These *Streptomyces* strains; EKR5.2, ESS7.8, ECR2.10, and EWL5.1, all have BGCs that code for compounds that are related to PGP traits, like ectoine (100% for all). The strains ESS7.8, ECR2.10, and EWL5.1 all have BGCs of the siderophore desferrioxamin B and E (100% for all), while the strain EKR5.2 has BGCs of the NI-siderophore FW0622 (62%), which is a new siderophore from the marine species *Verrucosispora* sp. FIM060022 (Zhao et al., 2019). Interestingly, strain ESS7.8 comprises the BGC of 6-methylsalicyclic acid (6-MeSA). Researchers reported that salicylates and related compounds could induce disease resistance in plants through the Induced Systematic Resistant pathway. The treatment of tobacco leaves with 6-MeSA increased the accumulation of the pathogenesis-related (PR) proteins PR1, β-1,3-glucanase, and chitinase and supported the plant’s resistance to the tobacco mosaic virus (Yalpani et al., 2001).

The genome of *Micrococcus* strain EWR3.9.1 comprises only one BGC of a terpene known as carotenoid (66%). The study of Kandasamy and Kathirvel (2024) showed that crude yellow pigment from endophytic *Micrococcus luteus* associated with *Avicennia marina* showed significant dose-dependent antioxidant and anticancer activity. Strain EWR3.9.1 may produce carotenoid which may comprise the antioxidant properties of host plant. Further research could elucidate the mechanisms behind this relationship and explore the potential applications of these findings in agricultural practices.

### Genome data mining

**Supplementary Table S6** displays the COG functional category classification of *Streptomyces* strains EKR5.2, ESS7.8, ECR2.10, and EWL5.1. These four strains comprise the highest number of genes relating to category K: transcription, with the following categories: E: amino acid metabolism and transport, and G: carbohydrate metabolism and transport. Strains ECR2.10 and EWL5.1 comprise more sequences at 402 and 329, respectively, belonging to category Q: secondary structure than strains EKR5.2 and ESS7.8, which contain only 219 and 229 sequences, respectively. The COG functional category classification of *Micrococcus* strain EWR3.9.1 showed different results when compared to these strains of *Streptomyces*. The most genes were found in category L, which is for replication and repair. The next most genes were found in categories E, which is for amino acid metabolism and transport, and the last few were found in category J, which is for translation. Additionally, the ratio of genes associated with category P, which pertains to inorganic ion transport and metabolism, is higher than that of the other four *Streptomyces* strains.

Functional annotation showed that four *Streptomyces* strains and one strain of *Micrococcus* contain genes relating to plant growth promotion to reduce stress on plants under drought and saline conditions (**Supplementary Tables S7**–**S11**). In the genomes of these five strains, genes that encode the osmo-protectant glycine betaine and proline have been found to help plants under stress. However, there were more genes encoding these proteins detected in *Streptomyces* strains than in *Micrococcus*. Genes encoding heat and osmotic pressure proteins were also detected in five strains, including chaperone proteins. However, only four strains of *Streptomyces* comprised more genes encoding ectoine production, of which only one gene was detected in the genome of *Micrococcus*. Also, only three strains of *Streptomyces*; EKR5.2, ECR2.10, and EWL5.1, have an *acd*s gene that encodes 1-aminocyclopropane-1-carboxylate deaminase. This correlated with the phenotypic data of these strains that produce ACC deaminase *in vitro.* These five strains also contain genes that encode biodegradation enzymes like amylase, cellulase, chitinase, and xylose isomerase, which find application in various industrial sectors.

In all strains of *Streptomyces* and *Micrococcus* EWR3.9.1, there are genes that encode antioxidants such as ferredoxin, flavodoxin, glutaredoxin, and thioredoxin. Only *Micrococcus* strain EWR3.9.1 contains redoxin. Thioredoxin and peroxiredoxins are non-enzymatic antioxidants that can detoxify any excess reactive oxidative scavenger (ROS) (Hopkins and Neumann, 2019). Glutaredoxins (Grxs) are small disulfide reductase enzymes that use glutathione and NADPH as cofactors. A few strains of bacteria produce Grxs as antioxidants to defend against oxidative stress and reveal the physiological roles of GrxD in oxidative stress protection (Saninjuk et al., 2023).

*Streptomyces* strain EKR5.2, which was isolated from high-salinity soil, had a gene that encodes peroxiredoxin. Peroxiredoxins (Prxs) or thioredoxin peroxidases (TPXs) are a group of thiol-specific antioxidant enzymes that help protect cells from oxidative damage (Kim et al., 2013).

*Streptomyces* strain ESS7.8 had genes that encoded cupredoxin and rubredoxin. Rubredoxin plays an important role in the reduction of superoxide and protect bacterial cell from oxidative (Coulter and Kurtz, 2001). These unique rubredoxins likely may play a crucial role in the survival and metabolic flexibility of *Streptomyces* strain ESS7.8, enabling them to thrive in environments where oxygen levels fluctuate.

Three strains of *Streptomyces*, EKR5.2, ECR2.10, and EWL5.1, including *Micrococcus* strain EWR3.9.1, comprise genes encoding IAA production. *Streptomyces* strain ESS7.8 and *Micrococcus* strain EWR3.9.1 contain genes encoding siderophore production. Only *Streptomyces* strains ESS7.8, ECR2.10, and EWL5.1 comprise genes encoding phenazine production, which is an antimicrobial compound. Moreover, the genomes of *Streptomyces* strain ESS7.8 and *Micrococcus* strain EWR3.9.1 contain genes encoding *L*-asparaginase II, an enzyme that can treat cancer (Darvishi et al., 2022). The genomes of these two strains comprise genes encoding lycopene production. The genomes of strains EKR5.2, ECR2.10 and EWL5.1 have genes encoding exopolysaccharide production. It was reported that exopolysaccharide can help plants to tolerate drought and salinity conditions (Bhagat et al., 2021). Seedling length vigor index (SLVI) of seedlings treated with *Streptomyces* strain EWL5.1 showed that this strain helped seedlings grow even when they were under 50 mM of salt stress. It may produce exopolysaccharide to help plants in salt stress conditions. This suggests that strain EWL5.1 may have positive effects due to both its production of IAA and ACC deaminase. Moreover, this strain had ability to produce exopolysaccharides, which may help the plant more resilient in harsh environments. We need to conduct further studies to clarify the mechanisms underlying this interaction and investigate the potential uses of these strains in agricultural practices. *Micrococcus* strain EWR3.9.1 contained genes related to metal resistance, copper resistance, and cadmium resistance transporters. This strain comprises the Nramp gene, which encodes a natural resistance-associated macrophage protein. In prokaryotes, Nramp plays a complex role in both nutrition and defense mechanisms. It was reported that the Nramps of *Salmonella typhimurium* and *Escherichia coli* displayed transport activity for Mn and are significantly stimulated after macrophage invasion (Kehres et al., 2000).

## Conclusion

Eucalyptus grown in different soil salinity levels yielded different numbers and biodiversity of both *Streptomyces* and non-*Streptomyces* strains. The endophytic actinobacteria isolated from plants grown on high salinity soil contained a substantial biodiversity of *Streptomyces* species groups and non-*Streptomyces*, including non-actinobacteria genera. Endophytic actinobacteria from this study were a good source of antifungal activity to inhibit severe leaf spot fungi in *Eucalyptus*. *In vitro* studies indicated that selected strains had PGP traits to promote plant growth in salinity conditions, such as ACC deaminase and IAA production. All isolates could produce IAA, which was a phytohormone to help the plant tolerate stressful conditions. One selected *Streptomyces* strain could support the seedling length vigor index (SLVI) of eucalyptus seedlings in salinity conditions and significantly increase the fresh weight of eucalyptus seedlings *in planta*. The genome analysis of four representative *Streptomyces* strains revealed that these strains contain various biosynthetic gene clusters (BGCs) involved in antibiotic production. The result of genome data mining of four *Streptomyces* strains and a strain of *Micrococcus* also reveals that these strains contain genes encoding PGP properties such as glycine-betaine, proline, IAA, and ACC deaminase production including antioxidants. In conclusion, endophytic actinobacteria found in *Eucalyptus* trees could inhibit fungal pathogens and exhibit PGP traits that could be used in the future as plant inoculum in saline soil. This suggests a promising avenue for agricultural practices, particularly in regions facing salinity challenges. By harnessing the beneficial properties of these endophytic actinobacteria, farmers may enhance crop resilience and productivity in adverse environmental conditions.

## Data Availability

The datasets presented in this study can be found in online repositories. The names of the repository/repositories and accession number(s) can be found in the article/**Supplementary Material**.
