# Supplementary material for: Phylogenetic affiliation of endophytic actinobacteria associated with red gum tree grown in salinity area and their plant growth promoting properties and suppression of phytopathogens, and genome data mining of selected strains
Source: Front Plant Sci. 2025 Nov 26;16:1610327. doi: 10.3389/fpls.2025.1610327 (PMC12689991; doi:10.3389/fpls.2025.1610327)
Supplement: Supplementary file 1 [file DataSheet1.pdf]

Supplementary Table

Phylogenetic affiliation of endophytic actinobacteria associated with red gum tree grown in salinity area and their plant growth promoting properties and suppression of phytopathogens, and genome data mining of selected strains

Onuma Kaewkla[1,2*], Kawintip Kiakhunthod[1,2], Sumalee Chookhampaeng[1], Busayarat Klinjantasorn[1,2], Piriya Klankeo[3], Winya Dungkaew[2]

Table S1. The three closest matches of non-actinobacteria isolated from surface-sterilized tissues based on 16S rRNA gene similarity. #The GenBank accession numbers are presented in Table S12.

**Isolate/ genus#**	**The 1st closest match**	**%**	**The 2nd closest match**	**%**	**The 3rd closest match**	**%**
	**Genus *Aureimonas***					
EWS3.8A	*Aureimonas phyllosphaerae* L9-753^T	97.8	*Aureimonas ureilytica* NBRC 106430^T	97.3	*Aureimonas flava* M2BS4Y-1^T	97.3
	**Genus *Bacillus***					
EKR6.16	*Bacillus tequilensis* KCTC 13622^T	99.6	*Bacillus cabrialesii* TE3^T	99.6	*Bacillus inaquosorum* KCTC 13429^T	99.6
EWS8.13	*Bacillus tequilensis* KCTC 13622^T	99.9	*Bacillus cabrialesii* TE3^T	99.9	*Bacillus inaquosorum* KCTC 13429^T	99.9
ESR3.34	*Bacillus siamensis* KCTC 13613^T	99.8	*Bacillus velezensis* CR-502^T	99.7	*Bacillus subtilis* NCIB 3610^T	99.5
	**Genus *Chryseobacterium***					
EBS5.19	*Chryseobacterium gleum* ATCC 35910^T	99.5	*Chryseobacterium arthrosphaerae* CC-VM-7^T	98.8	*Chryseobacterium indologenes* NBRC 14944^T	98.6
EBR3.16	*Chryseobacterium gleum* ATCC 35910^T	99.2	*Chryseobacterium arthrosphaerae* CC-VM-7^T	98.4	*Chryseobacterium indologenes* NBRC 14944^T	98.2
EKL5.7	*Chryseobacterium gleum* ATCC 35910^T	99.4	*Chryseobacterium arthrosphaerae* CC-VM-7^T	98.6	*Chryseobacterium indologenes* NBRC 14944^T	98.4
	**Genus *Deinococcus***					
EKL3.2.1	*Deinococcus daejeonensis* MJ27^T	99.0	*Deinococcus grandis* ATCC 43672^T	98.6	*Deinococcus xianganensis* Y35^T	98.4
EWSD8.20	*Deinococcus wulumuqiensis* R12^T	98.1	*Deinococcus radiodurans* DSM 20539^T	95.5	*Deinococcus indicus* Wt/1a^T	92.5
	**Genus *Massilia***					
EWR1.21	*Massilia haematophila* CCUG 38318^T	98.7	*Massilia terrae* J11^T	97.8	*Massilia rhizosphaerae* NEAU-GH312^T	97.6
EWR3.23	*Massilia tieshanensis* TS3^T	97.6	*Massilia niastensis* 5516S-1^T	97.1	*Massilia phyllostachyos* G4R7^T	97.1
ESR4.26	*Massilia oculi* CCUG 43427A^T	98.3	*Massilia timonae* CCUG 45783^T	97.8	*Massilia arenae* GEM5^T	97.0
	**Genus *Methylobacterium***					
EKL4.21	*Methylobacterium aerolatum* 5413S-11^T	99.6	*Methylobacterium persicinum* 002-165^T	98.1	*Methylobacterium radiotolerans* JCM 2831^T	96.9
ESS3.12	*Methylobacterium tardum* RB677^T	99.6	*Methylobacterium radiotolerans* JCM 2831^T	99.3	*Methylobacterium oryzae* CBMB20^T	98.9

EWS3.8	Methylobacterium dankookense SW08-7^T	99.7	*Methylobacterium symbioticum* SB0023/3^T	98.4	*Methylobacterium trifolii* TA73^T	97.1
	**Genus *Pseudomonas***					
EBR8.6	*Pseudomonas aeruginosa* JCM 5962^T	99.9	*Pseudomonas paraeruginosa* PA7^T	99.9	*Pseudomonas otitidis* MCC10330^T	98.2
EBR8.5	*Pseudomonas oryzihabitans* NBRC 102199^T	99.8	*Pseudomonas psychrotolerans* DSM 15758^T	99.8	*Pseudomonas rhizoryzae* RY24^T	99.1
EKR5.15	*Pseudomonas oryzihabitans* NBRC 102199^T	99.6	*Pseudomonas psychrotolerans* DSM 15758^T	99.6	*Pseudomonas rhizoryzae* RY24^T	98.9
EWR2.3	*Pseudomonas oryzihabitans* NBRC 102199^T	100	*Pseudomonas psychrotolerans* DSM 15758^T	100	*Pseudomonas rhizoryzae* RY24^T	99.2
EWR2.6	*Pseudomonas lactis* DSM 29167^T	99.3	*Pseudomonas pergaminensis* 1008^T	99.3	*Pseudomonas salmasensis* SWRI126^T	99.3
EWR8.11	*Pseudomonas paracarnis* V5/DAB/2/5^T	99.0	*Pseudomonas pergaminensis* 1008^T	99.0	*Pseudomonas lactis* DSM 29167^T	98.9
ESS2.5	*Pseudomonas pisciculturae* P115^T	98.7	*Pseudomonas pergaminensis* 1008^T	98.4	*Pseudomonas paracarnis* V5/DAB/2/5^T	98.4
	Genus Serratia					
EBR3.8S	*Serratia marcescens* ATCC 13880^T	99.5	*Serratia nematodiphila* DSM 21420^T	99.3	*Serratia surfactantfaciens* YD25^T	99.3
EKL4.22	*Serratia marcescens* ATCC 13880^T	99.9	*Serratia nematodiphila* DSM 21420^T	99.7	*Serratia surfactantfaciens* YD25^T	99.5
EKR6.9	*Serratia marcescens* ATCC 13880^T	99.6	*Serratia nematodiphila* DSM 21420^T	99.4	*Serratia surfactantfaciens* YD25^T	99.4
EKS4.13	*Serratia marcescens* ATCC 13880^T	99.4	*Serratia nematodiphila* DSM 21420^T	99.2	*Serratia surfactantfaciens* YD25^T	99.2
	Staphylococcus					
EKL4.16	*Staphylococcus ureilyticus* ATCC 49330^T	99.3	*Staphylococcus cohnii* subsp. *Cohnii* ATCC 29974^T	99.2	*Staphylococcus cohnii* subsp. *barensis* SC5^T	99.2
EWR6.6	*Staphylococcus hominis* subsp. *novobiosepticus* GTC 1228^T	99.5	*Staphylococcus hominis* subsp. *hominis* DSM 20328^T	99.2	*Staphylococcus caledonicus* H8/1^T	98.7
	**Genus *Stenotrophomonas***					
EKL7.20	*Stenotrophomonas maltophilia* MTCC 434^T	98.6	*Stenotrophomonas pavanii* DSM 25135^T	98.1	*Stenotrophomonas sepilia* SM-16975^T	98.1

Table S2. The salt tolerance and plant growth promoting properties of thirty-five isolates of endophytic actinobacteria. W, weak; 1, poor growth; 2, moderate growth; 3, good growth; N, negative result; 0, no inhibition; 1+, weak inhibition; 2+, moderate inhibition; 3+, good inhibition; 4+, strong inhibition. ACC, 1-aminocyclopropane-1-carboxylic acid; CMC, carboxymethyl cellulose; IAA, indole-3-acetic; LS6, *Pseudoplagiostroma eucalypti*; LB1, *Cladosporium* sp.

Isolates	Growth at different NaCl concentration (w/v)						Inhibition of *R. solanacearum* TISTR 2069 (%)	IAA (µg/ml)	Nitrogen fixation	ACC deaminase	Hydrolyze CMC (mm)	Inhibition of LS6	Inhibition of LB1	Phosphate solubilization
	1%	3%	5%	7%	9%	11%								
EWR 8.25	3	2	2	2	1	W	35.6	30.10	N	N	3.75	3+	2+	N
EWS 1.1	3	3	3	2	1	W	47.9	15.30	N	N	0	4+	3+	N
EWR 1.9	3	3	3	1	1	W	41.4	30.10	N	N	4.50	4+	2+	N
EWL 5.23	3	3	2	1	W	-	5.6	30.00	N	N	15.00	4-	2+	N
EWS 3.1	3	3	2	1	-	-	0	15.80	N	N	10.00	4+	2+	N
EWS 8.19	3	3	3	2	1	W	12.4	10.80	N	+	0	3+	2+	N
EWS 1.5	3	2	W	-	-	-	41.5	25.40	N	N	13.00	4+	2+	N
ECS 4.6	3	3	3	2	1	-	0	29.30	+	+	3.50	4+	1+	N
ECL 5.5	3	3	3	2	2	1	9.4	25.30	N	+	31.00	4+	2+	N
ECR 3.8	3	3	3	2	1	W	0	32.60	N	+	1.50	3+	1+	N
ECR 3.37	3	3	2	1	-	-	0	30.00	+	N	38.50	3+	1+	N
EBS 5.3	3	3	3	3	1	w	0	16.20	N	N	41.5	3+	2+	N
EBR 8.2	3	3	3	2	1	1	24.4	24.90	N	N	4.5	4+	2+	+
EBR 6.14	3	3	3	2	1	1	0	23.90	N	N	0.75	4+	3+	+
EBL 4.3	3	3	3	2	1	1	14.7	26.30	N	N	2.5	4+	3+	N

EKL 6.12	2	2	1	1	1	w	0	26.90	N	+	34	4+	3+	+
EKR 7.5	2	2	1	1	1	w	0	25.30	N	+	43.8	3+	1+	N
EKR 6.15	3	2	2	1	1	1	0	27.30	N	N	0	4+	2+	+
EKS 3.2	3	3	3	3	3	2	9.1	25.00	N	N	4	4+	2+	+
EKR 2.14	3	3	3	2	2	1	18.8	25.00	N	N	27.5	3+	3+	+
EKL 5.16	3	3	3	2	2	1	0	20.00	N	+	2.5	4+	3+	+
ESS 7.15	3	2	W	W	W	W	0	24.31	+	N	24.3	4+	4+	N
ESR 3.26	3	3	3	2	W	W	0	36.67	+	N	N	3+	3+	N
ESS 7.19	3	3	3	3	3	3	29.1	34.13	N	N	N	3+	3+	N
ESS 7.24	3	3	3	3	3	2	0	27.59	N	N	12.3	4+	3+	N
ESR 1.13	3	3	2	2	1	1	0	31.59	N	N	13	4+	3+	N
ECL 2.1	3	3	3	3	2	1	7.9	32.74	N	N	2.5	3+	3+	+
ECR 6.5	3	3	3	2	W	W	0	42.21	N	+	0	1+	0	N
ECL 7.27	3	3	3	3	3	2	0	39.86	N	N	19	3+	2+	N
ECR 5.32	3	3	3	2	2	2	0	12.36	N	+	6	4+	2+	+
ECR 6.25	3	3	2	1	W	W	0	18.93	+	+	24	4+	1+	N
ESR 5.20	3	3	3	2	2	2	7.5	34.13	N	N	0	4+	1+	N
ESR 8.21	3	3	2	2	2	1	3.8	28.57	N	N	0	4+	1+	N
ESL2.3	3	3	3	2	2	1	0	12.53	N	N	0	4+	1+	N
ESL8.5	3	3	3	2	2	2	0	31.65	N	N	0	4+	1+	N

Table S3. The distribution of BGCs of *Streptomyces* sp. EKR5.2 and the closely related type strain, *Streptomyces lannensis* JCM16578^T, based on "antiSMASH" prediction.

Type	Product	Span (nt)	Similarity (%)	Product	Span (nt)	Similarity (%)
	Streptomyces sp. EKR5.2			*Streptomyces lannensis* JCM16578^T		
RiPP-like	Informatipeptin	25,995 - 36,240	42	Informatipep-tin	252,174-262,389	42
NAPAA	ε-Poly-L-lysine	10,538 - 31,259	100	ε-Poly-L-lysine	429,972-450,819	100
Terpene	Hopene	1 - 8,862	53	Hopene	48,264-66,931	61
Terpene	Geosmin	1 - 3,841	100	Geosmin	495,542-517,740	100
Terpene	Albaflavenone	1 - 19,149	100	Albaflavenone	1-19,037	100
T2PKS, oligosac-charide	**Not detected**			Galtamycin C/ Galtamycin D	1-57,166	78
T2PKS	Spore pigment	1 - 21,556	66	Spore pigment	46,821-119,330	83
T3PKS	Flaviolin/1,3,6,8-tetrahydroxynaphthalene	1 - 19,702	100	**Not detected**		
T3PKS	**Not detect**			Alkylresorci-nol	1-39,409	100
NI-siderophore	NI-siderophore FW0622	1 - 15,998	62	FW0622	248,606-278,378	62
Other	Melanin	11,586 - 21,987	42	Melanin	333,884-344,291	42
Other	Ectoine	1 - 8,646	100	Ectoine	86,543-96,941	100

Table S4. The distribution of BGCs of *Streptomyces* sp. ESS7.8 and its closest type strain, *Streptomyces ardesiacus* NBRC 15402^T, based on "antiSMASH" prediction.

Type	Product	Span (nt)	Similarity (%)	Product	Span (nt)	Similarity (%)
***Streptomyces* sp. strain ESS7.8**				***Streptomyces ardesiacus* NBRC 15402^T**		
NRPS:, nucleoside	**Not detected**			Detoxin P1/detoxin P2/detoxin P3	49,270-102,495	75
NRPS	Thiazostatin, watasemycin A, watasemycin B, 2-hydroxyphenyl thiazoline enantiopyochelin, isopyochelin	173,179 - 268,488	100	Thiazostatin, watasemycin A, watasemycin B, 2-hydroxyphenylthiazoline enantiopyochelin, isopyochelin	224,502-319,890	100
NRP-metallo-phore	Coelichelin	112,538-170,908	100	Coelichelin	1,245,924-1,304,294	100
NRP-metallo-phore	Coelibactin	56,265-119,857	100	Coelibactin	1,428,017-1,491,517	100
Terpene	β-carotein	371,136-395,231	62	**Not detected**		
	Albaflavenone	202,835-223,848	100	Albaflavenone	778,230-799,243	100
	Geosmin	109,281-131,482	100	Geosmin	190,986-213,187	100
	Hopene	81,533 - 108,262	100	Hopene	560,033-586,762	100
	**Not detected**			Isorenieratene	668,873-692,929	62
RiPP-like	Informatipeptin	3,420 - 13,635	42	Informatipeptin	1,033,543-1,043,758	100
lanthipeptide-class-iii	SapB	181,235 - 204,024	100	SapB	464,248-487,034	100
T1PKS	Amphotericin B	209,397 - 269,933	60	**Not detected**		
	Butyrolactol A	52,391 - 93,244	86	Butyrolactol A	1-43,843	86
	6-methylsalicyclic acid	1 - 33,484	40	**Not detected**		
	**Not detected**			Macrotermycins	1-61,228	65

Type	Product	Span (nt)	Similarity (%)	Product	Span (nt)	Similarity (%)
T2PKS	Spore pigment	74,534 - 147,070	66	Spore pigment	855,015-927,551	66
	Enterocin	38,334 - 114,231	95	**Not detected**		
T3PKS	Alkylresorcinol	110,824 - 151,996	100	Alkylresorcinol	895,153-936,325	100
	Flaviolin, 1,3,6,8-tetrahydroxynaphthalene	323,318 - 364,442	100	Flaviolin, 1,3,6,8-tetrahydroxynaphthalene	128,513-169,637	100
	Germicidin	1,926 - 43,110	100	Germicidin	495,439-536,623	100
NI-siderophore	Desferrioxamin B, Desferrioxamine E	507,564 - 537,333	100	Desferrioxamin B, Desferrioxamine E	552,212-581,981	100
Ectoine	Ectoine	42,694 - 53,092	100	Ectoine	68,465-78,863	100
Indole	5-dimethylallyl indole-3-acetonitrile	305,352 - 326,479	100	5-dimethylallyl indole-3-acetonitrile	737,576-758,703	100

Table S5. The distribution of BGCs of *Streptomyces* sp. strains ECR2.10 and EWL5.1 based on "antiSMASH" prediction.

**Type**	**Product**	**Span (nt)**	Similarity (%)	Product	Span (nt)	Similarity (%)
***Streptomyces* sp. strain ECR2.10**				***Streptomyces* sp. strain EWL5.1**		
Terpene	Melanin	518,715 - 540,296	71	**Not detected**		
Terpene	Geosmin	288,409 - 310,571	100	Geosmin	6666219-6668399	100%
Terpene	Hopene	323,793 - 350,488	92	Hopene	7516017-7529773	92%
Terpene	Albaflavenone	126,825 - 147,910	100	Albaflavenone	5681016-5683483	100%
Terpene	Pentalenolactone	8,034 - 29,047	58	pentalenolactone	1-21694	58%
T1PKS	Methylated alkyl-resorcinol/methylated acyl-phloroglucinol	1 -4,869	50	**Not detected**		
T3PKS	Flaviolin/1,3,6,8-tetrahydroxynaphthalene	151,534 - 192,595	100	Flaviolin/1,3,6,8-tetrahydroxynaphthalene	1277478-1280491	100%
T3PKS	Germicidin	121,537 - 162,730	100	Germicidin	1-1185	100%
NRPS	Aurantimycin A	1 -50,626	100	**Not detected**		
Lanthipeptide-class-iii,RiPP-like	Informatipeptin	19,947 - 47,380	100	Informatipeptin	8170944-8185829	100%
NI-siderophore	Desferrioxamin B/ desferrioxamine E	61,749 - 91,518	100	Desferrioxamin B/ desferrioxamine E	3033355-3040682	100%
Betalactone	Cystargolide A/cystargolide B	96,950 - 124,569	90	Cystargolide A/cystargolide B	1-13771	90%
CDPS	Prunipeptin	5,055 - 25,765	100	Prunipeptin	170670-171864	100%
Other	Ectoine	85,171 - 95,575	100	Ectoine	1-3366	100%

Table S6. Number of sequences of each functional category COGs categories.

Functional category	Number of sequences				
	EKR5.2	ESS7.8	ECR2.10	EWL 5.1	EWR3.9.1
A: RNA processing and modification	4	3	3	3	0
B: Chromatin Structure and dynamics	1	2	1	1	0
C: Energy production and conversion	497	326	415	389	112
D: Cell cycle control and mitosis	83	72	66	66	25
E: Amino Acid metabolis and transport	525	439	535	527	171
F: Nucleotide metabolism and transport	132	124	130	130	66
G: Carbohydrate metabolism and transport	504	441	509	491	75
H: Coenzyme metabolis	168	157	174	166	73
I: Lipid metabolism	273	234	243	243	89
J: Tranlsation	245	235	233	233	161
K: Transcription	1034	723	843	837	128
L: Replication and repair	388	249	271	268	177
M: Cell wall/membrane/envelop biogenesis	313	272	309	309	78
N: Cell motility	4	5	6	6	0
O: Post-translational modification, protein turnover, chaperone functions	236	173	190	187	74
P: Inorganic ion transport and metabolism	267	221	212	211	115
Q: Secondary Structure	219	229	402	329	32
T: Signal Transduction	393	337	333	334	45
U: Intracellular trafficing and secretion	45	45	37	37	21
V: Defense mechanisms	165	155	148	148	36
S: Function Unknown	1369	1035	1208	1202	422

Table S7. Gene prediction for gene annotation of the genome of *Streptomyces* strain EKR5.2 relating to stress reduction and plant growth promoting proteins, biodegradation enzymes, and other compounds. PFAM: Protein Families Database; Ko: KEGG Orthology; KEGG: Kyoto Encyclopedia of Genes and Genomes.

Description	PFAM	KEGG_Ko
**Stress reduction: Glycine-betaine, proline**		
Glycine oxidase	DAO	ko:K03153
Glycine betaine	OpuAC	ko:K05845
Glycine betaine	ABC_tran,CBS	ko:K02000
Involved in the biosynthesis of the osmoprotectant glycine betaine. Catalyzes the oxidation of choline to betaine aldehyde and betaine aldehyde to glycine betaine at the same rate	GMC_oxred_C,GMC_oxred_N	ko:K00108
ABC-type proline glycine betaine transport systems, permease component	BPD_transp_1,OpuAC	ko:K05845, ko:K05846, ko:K02001, ko:K02002
Catalyzes the reversible interconversion of serine and glycine with tetrahydrofolate (THF) serving as the one-carbon carrier. This reaction serves as the major source of one-carbon groups required for the biosynthesis of purines, thymidylate, methionine, and other important biomolecules. Also exhibits THF- independent aldolase activity toward beta-hydroxyamino acids, producing glycine and aldehydes, via a retro-aldol mechanism	SHMT	ko:K00600
The glycine cleavage system catalyzes the degradation of glycine. The H protein shuttles the methylamine group of glycine from the P protein to the T protein	GCV_T,GCV_T_C	ko:K02437, ko:K00605
The glycine cleavage system catalyzes the degradation of glycine. The P protein binds the alpha-amino group of glycine through its pyridoxal phosphate cofactor	GDC-P	ko:K00281, ko:K00283
Glycine betaine transport	OpuAC	ko:K02002
Catalyzes the cleavage of 2-amino-3-ketobutyrate to glycine and acetyl-CoA	Aminotran_1_2	ko:K00639 ko:K00652, ko:K01906
ABC-type proline glycine betaine transport system permease component	BPD_transp_1	ko:K02001
Glycine D-amino acid oxidases (deaminating)	DAO	ko:K00273
PPIases accelerate the folding of proteins. It catalyzes the cis-trans isomerization of proline imidic peptide bonds in oligopeptides	Pro_isomerase	ko:K03767, ko:K03768
Catalyzes the attachment of proline to tRNA(Pro) in a two-step reaction proline is first activated by ATP to form Pro- AMP and then transferred to the acceptor end of tRNA(Pro). As ProRS can inadvertently accommodate and process non-cognate amino acids such as alanine and cysteine, to avoid such errors it has two additional distinct editing activities against alanine. One activity is designated as 'pretransfer' editing and involves the tRNA(Pro)-independent hydrolysis of activated Ala-AMP. The other activity is designated 'posttransfer' editing and involves deacylation of mischarged Ala-tRNA(Pro). The misacylated Cys- tRNA(Pro) is not edited by ProRS.	HGTP_anticodon,ProRS-C_1,tRNA-synt_2b	ko:K01881

Catalyzes the reduction of 1-pyrroline-5-carboxylate (PCA) to L-proline	F420_oxidored,P5CR_dimer	ko:K00286
PPIases accelerate the folding of proteins. It catalyzes the cis-trans isomerization of proline imidic peptide bonds in oligopeptides	Pro_isomerase	ko:K03768
Proline dehydrogenase	Pro_dh	ko:K00318
Belongs to the proline racemase family	Pro_racemase	ko:K01777
**Stress reduction: heat and osmotic pressure**		
Participates actively in the response to hyperosmotic and heat shock by preventing the aggregation of stress-denatured proteins, in association with DnaK and GrpE. It is the nucleotide exchange factor for DnaK and may function as a thermosensor. Unfolded proteins bind initially to DnaJ	GrpE	ko:K03687
Channel that opens in response to stretch forces in the membrane lipid bilayer. May participate in the regulation of osmotic pressure changes within the cell	MscL	ko:K03282
Part of the MsrPQ system that repairs oxidized periplasmic proteins containing methionine sulfoxide residues (Met-O), using respiratory chain electrons. Thus protects these proteins from oxidative-stress damage caused by reactive species of oxygen and chlorine generated by the host defense mechanisms. MsrPQ is essential for the maintenance of envelope integrity under bleach stress, rescuing a wide series of structurally unrelated periplasmic proteins from methionine oxidation. The catalytic subunit MsrP is non-stereospecific, being able to reduce both (R-) and (S-) diastereoisomers of methionine sulfoxide	Oxidored_molyb	
Prevents misfolding and promotes the refolding and proper assembly of unfolded polypeptides generated under stress conditions	Cpn60_TCP1	ko:K04077
Stress protein	TerD	ko:K05795
Putative stress-induced transcription regulator	ABATE,zf-CGNR	
Belongs to the universal stress protein A family	Usp	
Stress protein	TerD	ko:K05791 ko:K05791, ko:K05795
Universal stress protein	Usp	ko:K03695, ko:K03696
Part of a stress-induced multi-chaperone system, it is involved in the recovery of the cell from heat-induced damage, in cooperation with DnaK, DnaJ and GrpE	AAA,AAA_2,ClpB_D2-small,Clp_N, UVR	ko:K03696, ko:K03979
An essential GTPase which binds GTP, GDP and possibly (p)ppGpp with moderate affinity, with high nucleotide exchange rates and a fairly low GTP hydrolysis rate. Plays a role in control of the cell cycle, stress response, ribosome biogenesis and in those bacteria that undergo differentiation, in morphogenesis control	DUF1967,GTP1_OBG,MMR_HSR1	ko:K05792
ATP-dependent serine protease that mediates the selective degradation of mutant and abnormal proteins as well as certain short-lived regulatory proteins. Required for cellular homeostasis and for survival from DNA damage and developmental changes induced by stress. Degrades polypeptides processively to yield small peptide fragments that are 5 to 10 amino acids long. Binds to DNA in a double-stranded, site-specific manner	AAA,LON_substr_bdg,Lon_C	ko:K01338
Stress responsive A/B Barrel Domain	Dabb	
Heat shock 70 kDa protein	HSP70	ko:K04043

Ribosome-associated heat shock protein implicated in the recycling of the 50S subunit S4 paralog	S4	ko:K04762
Belongs to the small heat shock protein (HSP20) family	HSP20	ko:K13993
Negative regulator of class I heat shock genes (grpE- dnaK-dnaJ and groELS operons). Prevents heat-shock induction of these operons	HTH_DeoR,HrcA	ko:K03705
Heat shock protein binding	DUF3099	
HEAT repeats	HEAT_2	
Heat shock 70 kDa protein		ko:K18481
Catalyzes the circularization of gamma-N-acetyl- alpha,gamma-diaminobutyric acid (ADABA) to ectoine (1,4,5,6- tetrahydro-2-methyl-4-pyrimidine carboxylic acid), which is an excellent osmoprotectant	Ectoine_synth	ko:K06720
Ectoine hydroxylase	PhyH	ko:K10674
Ectoine hydroxyectoine ABC transporter solute-binding protein	SBP_bac_3	ko:K02030
**Stress reduction: Sodium tolerant**		
Pfam Sodium hydrogen exchanger	Na_H_Exchanger	ko:K03455
Belongs to the sodium solute symporter (SSF) (TC 2.A.21) family	SSF	ko:K03307
Belongs to the sodium solute symporter (SSF) (TC 2.A.21) family	SSF	ko:K14393
Na( ) H( ) antiporter that extrudes sodium in exchange for external protons	Na_H_antiport_1	ko:K03313
F(1)F(0) ATP synthase produces ATP from ADP in the presence of a proton or sodium gradient. F-type ATPases consist of two structural domains, F(1) containing the extramembraneous catalytic core and F(0) containing the membrane proton channel, linked together by a central stalk and a peripheral stalk. During catalysis, ATP synthesis in the catalytic domain of F(1) is coupled via a rotary mechanism of the central stalk subunits to proton translocation	ATP-synt_B,OSCP	ko:K02109, ko:K02113, ko:K02110
Belongs to the sodium solute symporter (SSF) (TC 2.A.21) family	SSF	ko:K03307, ko:K14393
Na( ) H( ) antiporter that extrudes sodium in exchange for external protons	Na_H_antiport_1, Thioredoxin_4	
**Stress reduction: Antioxidant**		
Bifunctional enzyme with both catalase and broad- spectrum peroxidase activity	peroxidase	ko:K03782
Peroxidase	Dyp_perox	ko:K07223, ko:K16301
Antioxidant protein with alkyl hydroperoxidase activity. Required for the reduction of the AhpC active site cysteine residues and for the regeneration of the AhpC enzyme activity	CMD	ko:K04756
Belongs to the glutathione peroxidase family	GSHPx	ko:K00432
Ferredoxin oxidoreductase	korB	ko:K00175
Ferredoxin oxidoreductase	korA	ko:K00174
Ferredoxin	-	ko:K04755
Ferredoxin	fdxA	-
4Fe-4S single cluster domain of Ferredoxin I	Fer4_13,Fer4_15	-
Ferredoxin	fdxG	ko:K05337
PFAM 4Fe-4S ferredoxin, iron-sulfur binding domain protein	hybA	ko:K00124

Multimeric flavodoxin	WrbA	-
Pyruvate ferredoxin/flavodoxin oxidoreductase	POR	-
Flavodoxin-like fold	FMN red	-
Belongs to the thioredoxin family	trxA	ko:K03671
Thioredoxin-like	Thioredoxin_7	-
Thioredoxin	-	ko:K03672
Thioredoxin	thiX	-
Belongs to the thioredoxin family	trxA	ko:K00384, ko:K03671
Highly conserved protein containing a thioredoxin domain	-	ko:K06888
Thioredoxin	ybbN	ko:K05838
Glutaredoxin	-	ko:K18917
Glutaredoxin	-	ko:K18917
PFAM Glutaredoxin 2	-	-
Peroxiredoxin activity	CMD	ko:K01607
Peroxiredoxin activity	yphJ	ko:K01607
**Biodegradation enzyme: Amylase**		
Alpha amylase, catalytic	Alpha-amylase,DUF3459	ko:K01187
Putative glucoamylase	DUF3131,Glycoamylase	
**Biodegradation enzyme: Cellulase**		
Belongs to the glycosyl hydrolase 5 (cellulase A) family	Cellulase	
Cellulase activity		
**Biodegradation enzyme:** chitinase		
Chitinase	CBM_5_12,Glyco_ hydro_19, Ricin_B_lectin	ko:K01183, ko:K03791
**Biodegradation enzyme:** Xylose isomerase		
Xylose isomerase	AP_endonuc_2	ko:K09136
Xylose isomerase domain protein TIM barrel	AP_endonuc_2,AP_endo nuc_2_N	
Belongs to the xylose isomerase family	AP_endonuc_2	ko:K01805
PFAM Xylose isomerase domain protein TIM barrel	AP_endonuc_2	
Acetyl xylan esterase (AXE1)	AXE1,DLH,Hydrolase_4, Peptidase_S15	ko:K06889, ko:K01060
**Biodegradation enzyme: Pactate lyase**		
Pectate lyase superfamily protein	Pectate_lyase_3	
**Biodegradation enzyme: Lipase**		
GDSL-like Lipase/Acylhydrolase family	Lipase_GDSL_2	
GDSL-like Lipase/Acylhydrolase	Lipase_GDSL,Lipase_G DSL_2	
GDSL-like Lipase/Acylhydrolase family	Lipase_GDSL_3	
**PGPB traits: Phosphate solubilization**		
Alkaline phosphatase	PhoD	
Alkaline phosphatase	PhoD,PhoD_N	ko:K01077,

		ko:K01113
Belongs to the alkaline phosphatase family	Alk_phosphatase	ko:K01077
Alkaline phosphatase	PhoD,PhoD_N	ko:K01113
Phosphatase	SpoIIE, PAS_3, DUF839	ko:K07093
Protein phosphatase 2C domain protein	GAF,GAF_2,HATPase_c_2,PAS,PAS_4,PAS_9,SpoIIE,sCache_3_2	
Serine phosphatase RsbU, regulator of sigma subunit	SpoIIE	
Protein phosphatase 2C domain protein	GAF,GAF_2,HATPase_c_2,PAS_4,SpoIIE,sCache_3_2	
Protein phosphatase 2C domain protein	GAF,HATPase_c_2,PAS,PAS_4,SpoIIE	
SMART protein phosphatase 2C domain protein	GAF,GAF_2,GAF_3,HATPase_c_2,PAS,PAS_4,SpoIIE	
Phosphatase	PP2C,PP2C_2	ko:K20074
Inorganic pyrophosphatase	Pyrophosphatase	
Sigma factor PP2C-like phosphatases	GAF_2,PAS_3,PAS_4,SpoIIE	
Endonuclease/Exonuclease/phosphatase family	Exo_endo_phos	
Ppx GppA phosphatase	Ppx-GppA	ko:K01524
Phosphatase	Y_phosphatase	
Dual specificity phosphatase, catalytic domain	DSPc	
Acid phosphatase	Acid_phosphat_B	
Acid phosphatase homologues	PAP2	ko:K19302
SMART protein phosphatase 2C domain protein		
Protein phosphatase 2C	PP2C_2	
Low molecular weight phosphatase family	LMWPc	
**PGPB traits: Siderophore production**		
Siderophore-interacting protein	FAD_binding_9,SIP	
Siderophore-interacting protein	FAD_binding_9,SIP	ko:K14698
Siderophore biosynthesis protein domain	Acetyltransf_8	
**PGPB traits: IAA production**		
Indole-3-glycerol phosphate synthase	IGPS	ko:K01609
The beta subunit is responsible for the synthesis of *L*- tryptophan from indole and *L*-serine	PALP	ko:K01696
The alpha subunit is responsible for the aldol cleavage of indole glycerol phosphate to indole and glyceraldehyde 3- phosphate	Trp_syntA	ko:K01695
Heme-dependent dioxygenase that catalyzes the oxidative cleavage of the L-tryptophan (L-Trp) pyrrole ring and converts L- tryptophan to N-formyl-L-kynurenine. Catalyzes the oxidative cleavage of the indole moiety	Trp_dioxygenase	ko:K00453
**PGPB traits: ACC deaminase production**		
1-aminocyclopropane-1-carboxylate deaminase (acds)	PALP	ko:K01505
1-aminocyclopropane-1-carboxylate deaminase (acds)	PALP	ko:K05396

**Others**		
Phytoene	Amino_oxidase	ko:K01854
Phytoene synthase	SQS_PSY	ko:K02291
Squalene phytoene synthase	SQS_PSY	
Pyrimidine reductase, riboflavin biosynthesis	RibD_C	ko:K00082
Riboflavin synthase, alpha	Lum_binding	ko:K00793
Exopolysaccharide biosynthesis protein related to N-acetylglucosamine-1-phosphodiester alpha-N-acetylglucosaminidase	Metallophos,NAGPA	-
Exopolysaccharide biosynthesis protein related to N-acetylglucosamine-1-phosphodiester alpha-N-acetylglucosaminidase	NAGPA, SPOR	

Table S8. Gene prediction for gene annotation of the genome of *Streptomyces* strain ESS7.8 relating to stress reduction and plant growth promoting proteins, biodegradation enzymes, and other compounds. PFAM: Protein Families Database; Ko: KEGG Orthology; KEGG: Kyoto Encyclopedia of Genes and Genomes.

Description	PFAM	KEGG_Ko
**Stress reduction: Glycine-betaine, proline**		
ABC-type proline glycine betaine transport systems, permease component	BPD_transp_1,OpuAC	ko:K05845, ko:K05846
Glycine betaine	OpuAC	ko:K05845
ABC-type proline glycine betaine transport system permease component	BPD_transp_1,OpuAC	ko:K02001, ko:K02002
Involved in the biosynthesis of the osmoprotectant glycine betaine. Catalyzes the oxidation of choline to betaine aldehyde and betaine aldehyde to glycine betaine at the same rate	GMC_oxred_C,GMC_oxred_N	ko:K00108
glycine betaine transport	OpuAC	ko:K02002
Catalyzes the reduction of 1-pyrroline-5-carboxylate (PCA) to L-proline	F420_oxidored,P5CR_dimer	ko:K00286
**Stress reduction: heat and osmotic pressure**		
Participates actively in the response to hyperosmotic and heat shock by preventing the aggregation of stress-denatured proteins, in association with DnaK and GrpE. It is the nucleotide exchange factor for DnaK and may function as a thermosensor. Unfolded proteins bind initially to DnaJ	GrpE	ko:K03687
Part of a stress-induced multi-chaperone system, it is involved in the recovery of the cell from heat-induced damage, in cooperation with DnaK, DnaJ and GrpE	AAA,AAA_2,ClpB_D2-small,Clp_N	ko:K03695, ko:K03696
Heat shock 70 kDa protein	HSP70	ko:K04043
Channel that opens in response to stretch forces in the membrane lipid bilayer. May participate in the regulation of osmotic pressure changes within the cell	MscL	ko:K03282
Catalyzes the circularization of gamma-N-acetyl-alpha,gamma-diaminobutyric acid (ADABA) to ectoine (1,4,5,6- tetrahydro-2-methyl-4-pyrimidine carboxylic acid), which is an excellent osmoprotectant	Ectoine_synth	ko:K06720
ectoine hydroxylase	PhyH	ko:K10674
Ectoine hydroxyectoine ABC transporter solute-binding protein	SBP_bac_3	ko:K02030
Belongs to the universal stress protein A family	Usp	
Stress protein	TerD	ko:K05791, ko:K05795
**Stress reduction: Antioxidant**		
Serves to protect cells from the toxic effects of hydrogen peroxide	Catalase, Catalase-rel	ko:K03781
Bifunctional enzyme with both catalase and broad- spectrum peroxidase activity	peroxidase	ko:K03782

Antioxidant protein with alkyl hydroperoxidase activity. Required for the reduction of the AhpC active site cysteine residues and for the regeneration of the AhpC enzyme activity	CMD	
COG0189 Glutathione synthase Ribosomal protein S6 modification enzyme (glutaminyl transferase)		
Glutathione S-transferase	GST_C_2,GST_N_2	ko:K07393
Belongs to the glutathione peroxidase family	GSHPx	ko:K00432
alkyl hydroperoxide reductase Thiol specific antioxidant Mal allergen	AhpC-TSA	ko:K03386
peroxidase	Dyp_perox	ko:K15733
Ferredoxin	fdxG	ko:K05337
Ferredoxin	fdxA	-
Ferredoxin	DUF326	-
Ferredoxin	Fer4_13,Fer4_15,Fer4_19	ko:K05337
Flavodoxin	FMN_red	-
Flavodoxin	FMN_red	-
Ferredoxin oxidoreductase	korB	ko:K00175
Ferredoxin oxidoreductase	korA	ko:K00174
Multimeric flavodoxin WrbA	FMN_red	-
Glutaredoxin	-	ko:K18917
Glutaredoxin	-	ko:K18917
PFAM Glutaredoxin 2	DUF836	-
Thioredoxin	thiX	-
Protein conserved in bacteria containing thioredoxin-like domain	Suc_Fer-like	-
Belongs to the thioredoxin family	SEC-C	-
Belongs to the thioredoxin family	trxA	ko:K03671
Highly conserved protein containing a thioredoxin domain	GlcNAc_2-epim,Thioredox_DsbH	ko:K06888
Thioredoxin	Thioredoxin_5	-
Belongs to the thioredoxin family	trxA	ko:K00384, ko:K03671
Thioredoxin	Thioredoxin_4	-
Belongs to the thioredoxin family	trxA2	ko:K03671
Thioredoxin	ybbN	ko:K05838
Protein conserved in bacteria containing thioredoxin-like domain	Suc_Fer-like	-
DSBA-like thioredoxin domain	Thioredoxin_4	-
Protein conserved in bacteria containing thioredoxin-like domain	Suc_Fer-like	-
Cupredoxin-like domain	Cupredoxin_1	-
Rubredoxin-like zinc ribbon domain (DUF35_N)	DUF35_N	ko:K07068
**Stress reduction: Others**		
Arsenical-resistance protein	arsB	ko:K03325, ko:K03741

Trehalose synthase	Alpha-amylase,Malt_amylase C	ko:K05343
**Biodegradation enzyme: Lipase**	Lipase_GDSL_2	-
GDSL-like Lipase/Acylhydrolase family	LIP	-
Secretory lipase	COesterase	ko:K03929
Belongs to the type-B carboxylesterase lipase family	Lipase_GDSL,Lipase_GDSL_2	-
**Plant growth promoting; phosphatase**		
Phosphatase	SpoIIE	-
Phosphoesterase, PA-phosphatase related	PAP2	ko:K19302
Alkaline phosphatase	PhoD,PhoD_N	ko:K01113
**Biodegradation enzyme: Chitinase**		
Chitinase	CBM_5_12,Glyco_hydro_19,Ricin_B_lectin	ko:K01183,ko:K03791
Chitinase C	CBM_5_12,ChiC,Glyco_hydro_18	ko:K01183
**Biodegradation enzyme: xylose isomerase**		
Xylose isomerase-like TIM barrel	AP_endonuc_2	-
Xylose isomerase	AP_endonuc_2	ko:K09136
Belongs to the xylose isomerase family	AP_endonuc_2	ko:K01805
**Plant growth promoting: siderophore production**		
Siderophore-interacting protein	FAD_binding_9,SIP	ko:K14698
Siderophore biosynthesis protein domain	Acetyltransf_8	
**Biodegradation enzyme: Pectate lyase**		
Pectate lyase	Pec_lyase_C	ko:K01728
Pectin esterase	Pectinesterase	ko:K01051
**Other compounds**		
Lycopene cyclase	Amino_oxidase	ko:K09879
Phytoene	Amino_oxidase	ko:K10027
Phytoene synthase	SQS_PSY	ko:K02291
Riboflavin synthase, alpha	Lum_binding	ko:K00793
Cobalamin (vitamin B12) biosynthesis CbiX protein		
Polysaccharide deacetylase	Polysacc_deac_1	-
Phenazine biosynthesis protein PhzF	PhzC-PhzF	-
Polyketide cyclase / dehydrase and lipid transport	Polyketide_cyc2	-
L-asparaginase II	Asparaginase_II	-
Protein involved in exopolysaccharide biosynthesis	Wzz	-

Table S9. Gene prediction for gene annotation of the genome of *Streptomyces* strain ECR2.10 relating to stress reduction and plant growth promoting proteins, biodegradation enzymes, and other compounds. PFAM: Protein Families Database; Ko: KEGG Orthology; KEGG: Kyoto Encyclopedia of Genes and Genomes.

Description	PFAM	KEGG_KO
**Stress reduction: Glycine-betaine, proline**		
The glycine cleavage system catalyzes the degradation of glycine. The P protein binds the alpha-amino group of glycine through its pyridoxal phosphate cofactor (gcvP)	GDC-P	ko:K00281,ko:K00283
Catalyzes the cleavage of 2-amino-3-ketobutyrate to glycine and acetyl-CoA (bioF)	Aminotran_1_2	ko:K00652,ko:K01906
Catalyzes the cleavage of 2-amino-3-ketobutyrate to glycine and acetyl-CoA (kbl)	Aminotran_1_2	ko:K00639
Involved in the biosynthesis of the osmoprotectant glycine betaine. Catalyzes the oxidation of choline to betaine aldehyde and betaine aldehyde to glycine betaine at the same rate	GMC_oxred_C,GMC_oxred_N	ko:K00108
ABC-type proline glycine betaine transport system permease component (proW)	BPD_transp_1	ko:K02001
Catalyzes the reversible interconversion of serine and glycine with tetrahydrofolate (THF) serving as the one-carbon carrier. This reaction serves as the major source of one-carbon groups required for the biosynthesis of purines, thymidylate, methionine, and other important biomolecules. Also exhibits THF- independent aldolase activity toward beta-hydroxyamino acids, producing glycine and aldehydes, via a retro-aldol mechanism (glyA)	SHMT	ko:K00600
ABC-type proline glycine betaine transport system permease component (opuAB)	BPD_transp_1,OpuAC	ko:K02001,ko:K02002
Involved in the biosynthesis of the osmoprotectant glycine betaine. Catalyzes the oxidation of choline to betaine aldehyde and betaine aldehyde to glycine betaine at the same rate (betA)	GMC_oxred_C,GMC_oxred_N	ko:K00108
Catalyzes the attachment of glycine to tRNA(Gly) (glyQS)	HGTP_anticodon,tRNA-synt_2b	ko:K01880
The glycine cleavage system catalyzes the degradation of glycine. The H protein shuttles the methylamine group of glycine from the P protein to the T protein (gcvH)	GCV_H	ko:K02437
The glycine cleavage system catalyzes the degradation of glycine (gcvT)	GCV_T,GCV_T_C	ko:K00605
Glycine betaine	OpuAC	ko:K05845
Glycine betaine transport (proX)	OpuAC	ko:K02002
Glycine betaine (proV)	OpuAC	ko:K02000
Belongs to the proline racemase family	Pro_racemase	ko:K01777
Catalyzes the reduction of 1-pyrroline-5-carboxylate (PCA) to L-proline (proC)	F420_oxidored,P5CR_dimer	ko:K00286
(proS) Catalyzes the attachment of proline to tRNA(Pro) in a two-step reaction proline is first activated by ATP to form Pro-AMP and then transferred to the acceptor end of tRNA(Pro). As ProRS can inadvertently accommodate and process non-cognate	HGTP_anticodon,tRNA-synt_2b,tRNA_edit	ko:K01881

amino acids such as alanine and cysteine, to avoid such errors it has two additional distinct editing activities against alanine. One activity is designated as 'pretransfer' editing and involves the tRNA(Pro)-independent hydrolysis of activated Ala-AMP. The other activity is designated 'posttransfer' editing and involves deacylation of mischarged Ala-tRNA(Pro). The misacylated Cys- tRNA(Pro) is not edited by ProRS		
Removes 5-oxoproline from various penultimate amino acid residues except L-proline (pcp)	Peptidase_C15	ko:K01304
**Stress reduction: heat and osmotic pressure**		
Participates actively in the response to hyperosmotic and heat shock by preventing the aggregation of stress-denatured proteins, in association with DnaK and GrpE. It is the nucleotide exchange factor for DnaK and may function as a thermosensor. Unfolded proteins bind initially to DnaJ	GrpE	ko:K03687
Channel that opens in response to stretch forces in the membrane lipid bilayer. May participate in the regulation of osmotic pressure changes within the cell	MscL	ko:K03282
Part of the MsrPQ system that repairs oxidized periplasmic proteins containing methionine sulfoxide residues (Met-O), using respiratory chain electrons. Thus protects these proteins from oxidative-stress damage caused by reactive species of oxygen and chlorine generated by the host defense mechanisms. MsrPQ is essential for the maintenance of envelope integrity under bleach stress, rescuing a wide series of structurally unrelated periplasmic proteins from methionine oxidation. The catalytic subunit MsrP is non-stereospecific, being able to reduce both (R-) and (S-) diastereoisomers of methionine sulfoxide	-	-
Stress protein	-	ko:K05792
Prevents misfolding and promotes the refolding and proper assembly of unfolded polypeptides generated under stress conditions (groL2)	-	ko:K04077
Part of a stress-induced multi-chaperone system, it is involved in the recovery of the cell from heat-induced damage, in cooperation with DnaK, DnaJ and GrpE	AAA,AAA_2,ClpB_D2-small,Clp_N,UVR	ko:K03696
A protein kinase that phosphorylates Ser and Thr residues. Probably acts to suppress the effects of stress linked to accumulation of reactive oxygen species. Probably involved in the extracytoplasmic stress response	-	-
Part of a stress-induced multi-chaperone system, it is involved in the recovery of the cell from heat-induced damage, in cooperation with DnaK, DnaJ and GrpE (clpB)	AAA,AAA_2,ClpB_D2-small,Clp_N	ko:K03695,ko:K03696
May be involved in the stabilization of the cell envelope or may interact with the sensor protein CseC to modulate its activity, in response to cell envelope stress (cseA)	-	-
Prevents misfolding and promotes the refolding and proper assembly of unfolded polypeptides generated under stress conditions (gro2)	Cpn60_TCP1	ko:K04077
Stress protein (terE)	TerD	ko:K05795
(lepA) Required for accurate and efficient protein synthesis under certain stress conditions. May act as a fidelity factor of the translation reaction, by catalyzing a one-codon backward translocation of tRNAs on improperly translocated ribosomes.	EFG_C,GTP_EFTU,GTP_EFTU_D2,LepA_C	ko:K03596

Back- translocation proceeds from a post-translocation (POST) complex to a pre-translocation (PRE) complex, thus giving elongation factor G a second chance to translocate the tRNAs correctly. Binds to ribosomes in a GTP-dependent manner		
An essential GTPase which binds GTP, GDP and possibly (p)ppGpp with moderate affinity, with high nucleotide exchange rates and a fairly low GTP hydrolysis rate. Plays a role in control of the cell cycle, stress response, ribosome biogenesis and in those bacteria that undergo differentiation, in morphogenesis control (obg)	-	ko:K03979
Stress protein	-	ko:K05791
ATP-dependent serine protease that mediates the selective degradation of mutant and abnormal proteins as well as certain short-lived regulatory proteins. Required for cellular homeostasis and for survival from DNA damage and developmental changes induced (lon) by stress. Degrades polypeptides processively to yield small peptide fragments that are 5 to 10 amino acids long. Binds to DNA in a double-stranded, site-specific manner	-	ko:K01338
Stress protein	TerD	ko:K05795
Stress protein	TerD	ko:K05791, ko:K05795
Stress protein	-	ko:K05791
Part of a stress-induced multi-chaperone system, it is involved in the recovery of the cell from heat-induced damage, in cooperation with DnaK, DnaJ and GrpE	-	ko:K03696
**Ectoine**		
Catalyzes the circularization of gamma-N-acetyl- alpha,gamma-diaminobutyric acid (ADABA) to ectoine (1,4,5,6- tetrahydro-2-methyl-4-pyrimidine carboxylic acid), which is an excellent osmoprotectant (ectC)	Ectoine_synth	ko:K06720
ectoine hydroxylase (ectD)	Ectoine_synth	ko:K10674
Belongs to the class-III pyridoxal-phosphate-dependent aminotransferase family (ectB)	Aminotran_3	ko:K00836
L-2,4-diaminobutyric acid acetyltransferase (ectA)	Acetyltransf_1	ko:K06718
**Stress reduction: Proline**		
Belongs to the proline racemase family	Pro_racemase	ko:K01777
Catalyzes the reduction of 1-pyrroline-5-carboxylate (PCA) to L-proline (proC)	F420_oxidored,P5CR_dimer	ko:K00286
(proS) Catalyzes the attachment of proline to tRNA(Pro) in a two-step reaction proline is first activated by ATP to form Pro-AMP and then transferred to the acceptor end of tRNA(Pro). As ProRS can inadvertently accommodate and process non-cognate amino acids such as alanine and cysteine, to avoid such errors it has two additional distinct editing activities against alanine. One activity is designated as 'pretransfer' editing and involves the tRNA(Pro)-independent hydrolysis of activated Ala-AMP. The other activity is designated 'posttransfer' editing and involves deacylation of mischarged Ala-tRNA(Pro). The misacylated Cys- tRNA(Pro) is not edited by ProRS	HGTP_anticodon,tRNA-synt_2b,tRNA_edit	ko:K01881
Removes 5-oxoproline from various penultimate amino acid residues except L-proline (pcp)	Peptidase_C15	ko:K01304

**Sodium**		
Belongs to the sodium solute symporter (SSF) (TC 2.A.21) family	SSF	ko:K14393
Na( ) H( ) antiporter that extrudes sodium in exchange for external protons (nhaA)	Na_H_antiport_1	ko:K03313
F(1)F(0) ATP synthase produces ATP from ADP in the presence of a proton or sodium gradient. F-type ATPases consist of two structural domains, F(1) containing the extramembraneous catalytic core and F(0) containing the membrane proton channel, linked together by a central stalk and a peripheral stalk. During catalysis, ATP synthesis in the catalytic domain of F(1) is coupled via a rotary mechanism of the central stalk subunits to proton translocation (atpE)	ATP-synt_C	ko:K02110
PFAM sodium calcium exchanger	Na_Ca_ex	ko:K07301
Sodium/hydrogen exchanger family	-	-
**Stress reduction: ACC deaminase**		
1-aminocyclopropane-1-carboxylate deaminase (acds)	PALP	ko:K01505
**Stress reduction: catalase, peroxidase**		
Bifunctional enzyme with both catalase and broad- spectrum peroxidase activity (katG)	peroxidase	ko:K03782
catalase activity		-
Antioxidant protein with alkyl hydroperoxidase activity. Required for the reduction of the AhpC active site cysteine residues and for the regeneration of the AhpC enzyme activity (ahpD)	CMD	ko:K04756
Antioxidant protein with alkyl hydroperoxidase activity. Required for the reduction of the AhpC active site cysteine residues and for the regeneration of the AhpC enzyme activity (pcaC_2)	-	-
peroxidase	Dyp_perox	ko:K15733
peroxidase (efeN)	Dyp_perox	ko:K16301
Ferredoxin	Fer4_13,Fer4_15	-
ferredoxin	fdxG	ko:K05337
Ferredoxin	Fer4_13,Fer4_15	-
Ferredoxin	fdxA	-
Ferredoxin	Fer4_13,Fer4_15	-
Ferredoxin oxidoreductase	korA	ko:K00174
Ferredoxin oxidoreductase	korB	ko:K00175
4Fe-4S single cluster domain of Ferredoxin I	Fer4_13,Lactamase_B	-
PFAM 4Fe-4S ferredoxin, iron-sulfur binding domain protein	Fer4_11,Fer4_3,Fer4_4 ,Fer4_7	ko:K00124
Flavodoxin reductases ferredoxin-NADPH reductases family 1	FAD_binding_6,Fer2, NAD_binding_1	-
Flavodoxin reductases ferredoxin-NADPH reductases family 1	FAD_binding_6,Fer2, Metal_hydrol,NAD_binding_1	-
Flavodoxin-like fold	FMN_red,Flavodoxin_2	-
Multimeric flavodoxin WrbA	FMN_red	-

Thioredoxin	Thioredoxin_4	-
Thioredoxin	thiX	-
protein conserved in bacteria containing thioredoxin-like domain	Suc_Fer-like	-
DSBA-like thioredoxin domain	DSBA	-
Belongs to the thioredoxin family	trxA	ko:K00384, ko:K03671
Highly conserved protein containing a thioredoxin domain	GlcNAc_2-epim,Thioredox_DsbH	ko:K06888
belongs to the thioredoxin family	trxA	ko:K03671
Thioredoxin	Thioredoxin_4	-
Thioredoxin	ybbN	ko:K05838
belongs to the thioredoxin family	trxA2	ko:K03671
belongs to the thioredoxin family	SEC-C	-
Thioredoxin	Thioredoxin_4	-
Glutaredoxin	-	ko:K18917
Glutaredoxin	Glutaredoxin	ko:K18917
PFAM Glutaredoxin 2	DUF836	-
**Stress reduction: Glutathione**		
Glutathione S-transferase (ygiG)	GST_C_2,GST_N_2	ko:K07393
COG0189 Glutathione synthase Ribosomal protein S6 modification enzyme (glutaminyl transferase)	-	-
Thiolesterase that catalyzes the hydrolysis of S-D- lactoyl-glutathione to form glutathione and D-lactic acid	-	-
Belongs to the glutathione peroxidase family (btuE)	GSHPx	ko:K00432
**Biodegradation enzyme: Amylase**		
Maltogenic Amylase, C-terminal domain	-	-
alpha amylase, catalytic	Alpha-amylase,DUF3459	ko:K01187
**Biodegradation enzyme: cellulase**		
Belongs to the glycosyl hydrolase 5 (cellulase A) family		-
Belongs to the glycosyl hydrolase 11 (cellulase G) family (xlnB)	CBM_2,Glyco_hydro_10,Ricin_B_lectin	ko:K01181
Belongs to the glycosyl hydrolase 12 (cellulase H) family		-
Belongs to the glycosyl hydrolase 12 (cellulase H) family (celA1)	ko:K01179	ko:K01179
Biodegradation enzyme:Xylose isomerase		
Belongs to the xylose isomerase family (xylA)	AP_endonuc_2	ko:K01805
PFAM Xylose isomerase domain protein TIM barrel		-
Xylose isomerase-like TIM barrel		-
Acetyl xylan esterase	AXE1	ko:K01060
Biodegradation enzyme: pectin hydrolysis		
Pectate lyase		-
Pectin esterase	Pectin esterase	ko:K01051
**Biodegradation enzyme: lipase**		

Lipase (class 3)	Lipase_3	ko:K01046
**PGPB: Phosphatase**		
Alkaline phosphatase (phoD)	PhoD,PhoD_N	ko:K01113
SMART protein phosphatase 2C domain protein (prpM4)	-	-
**PGPB: IAA production**		
Siderophore biosynthesis protein domain	-	-
Siderophore-interacting protein (viuB)	-	-
Siderophore-interacting protein (sidE)	-	-
**PGPB: IAA production**		
The alpha subunit is responsible for the aldol cleavage of indoleglycerol phosphate to indole and glyceraldehyde 3-phosphate (trpA)	Trp_syntA	ko:K01695
The beta subunit is responsible for the synthesis of L-tryptophan from indole and L-serine (trpB)	PALP	ko:K01696
**Phenazine production**		
Phenazine biosynthesis protein PhzF (phZC)	-	-
Catalyzes the Claisen rearrangement of chorismate to prephenate	CM_2	ko:K04093
Phenazine biosynthesis PhzC PhzF protein (phzF)	-	-
Phenazine biosynthesis-like protein (phzF)	-	-
**Others: phytoene**		
phytoene synthase (crtB)	SQS_PSY	ko:K02291
Squalene phytoene synthase (hopE)	-	-
phytoene (crtl2)	Amino_oxidase	ko:K01854
**Others: riboflavin**		
Pyrimidine reductase, riboflavin biosynthesis	RibD_C	ko:K00082
Catalyzes the formation of 6,7-dimethyl-8- ribityllumazine by condensation of 5-amino-6-(D- ribitylamino)uracil with 3,4-dihydroxy-2-butanone 4-phosphate. This is the penultimate step in the biosynthesis of riboflavin (ribH)	DMRL_synthase	ko:K00794
Catalyzes the conversion of D-ribulose 5-phosphate to formate and 3,4-dihydroxy-2-butanone 4-phosphate (ribBA)	DHBP_synthase,GTP_cyclohydro2	ko:K14652
riboflavin synthase, alpha (ribE)	Lum_binding	ko:K00793
Exopolysaccharide biosynthesis protein related to N-acetylglucosamine-1-phosphodiester alpha-N-acetylglucosaminidase (NAGPA)	-	-

Table S10. Gene prediction for gene annotation of the genome of *Streptomyces* strain EWL5.1 relating to stress reduction and plant growth promoting proteins, biodegradation enzymes, and other compounds. PFAM: Protein Families Database; Ko: KEGG Orthology; KEGG: Kyoto Encyclopedia of Genes and Genomes.

**Description**	**PFAM**	**KEGG_KO**
**Stress reduction: Glycine-betaine, proline**		
The glycine cleavage system catalyzes the degradation of glycine. The P protein binds the alpha-amino group of glycine through its pyridoxal phosphate cofactor (gcvP)	GDC-P	ko:K00281,ko:K00283
Catalyzes the cleavage of 2-amino-3-ketobutyrate to glycine and acetyl-CoA (bioF)	Aminotran_1_2	ko:K00652,ko:K01906
Catalyzes the cleavage of 2-amino-3-ketobutyrate to glycine and acetyl-CoA (kbl)	Aminotran_1_2	ko:K00639
Involved in the biosynthesis of the osmoprotectant glycine betaine. Catalyzes the oxidation of choline to betaine aldehyde and betaine aldehyde to glycine betaine at the same rate	GMC_oxred_C,GMC_oxred_N	ko:K00108
ABC-type proline glycine betaine transport system permease component (proW)	BPD_transp_1	ko:K02001
Catalyzes the reversible interconversion of serine and glycine with tetrahydrofolate (THF) serving as the one-carbon carrier. This reaction serves as the major source of one-carbon groups required for the biosynthesis of purines, thymidylate, methionine, and other important biomolecules. Also exhibits THF- independent aldolase activity toward beta-hydroxyamino acids, producing glycine and aldehydes, via a retro-aldol mechanism (glyA)	SHMT	ko:K00600
ABC-type proline glycine betaine transport system permease component (opuAB)	BPD_transp_1,OpuAC	ko:K02001,ko:K02002
Involved in the biosynthesis of the osmoprotectant glycine betaine. Catalyzes the oxidation of choline to betaine aldehyde and betaine aldehyde to glycine betaine at the same rate (betA)	GMC_oxred_C,GMC_oxred_N	ko:K00108
Catalyzes the attachment of glycine to tRNA(Gly) (glyQS)	HGTP_anticodon,tRNA-synt_2b	ko:K01880
The glycine cleavage system catalyzes the degradation of glycine. The H protein shuttles the methylamine group of glycine from the P protein to the T protein (gcvH)	GCV_H	ko:K02437
The glycine cleavage system catalyzes the degradation of glycine (gcvT)	GCV_T,GCV_T_C	ko:K00605
Glycine betaine	OpuAC	ko:K05845
Glycine betaine transport (proX)	OpuAC	ko:K02002
Glycine betaine (proV)	OpuAC	ko:K02000
**Stress reduction: heat and osmotic pressure**		
Participates actively in the response to hyperosmotic and heat shock by preventing the aggregation of stress-denatured proteins, in association with DnaK and GrpE. It is the nucleotide exchange factor for DnaK and may function as a thermosensor. Unfolded proteins bind initially to DnaJ	GrpE	ko:K03687

Channel that opens in response to stretch forces in the membrane lipid bilayer. May participate in the regulation of osmotic pressure changes within the cell	MscL	ko:K03282
Part of the MsrPQ system that repairs oxidized periplasmic proteins containing methionine sulfoxide residues (Met-O), using respiratory chain electrons. Thus protects these proteins from oxidative-stress damage caused by reactive species of oxygen and chlorine generated by the host defense mechanisms. MsrPQ is essential for the maintenance of envelope integrity under bleach stress, rescuing a wide series of structurally unrelated periplasmic proteins from methionine oxidation. The catalytic subunit MsrP is non-stereospecific, being able to reduce both (R-) and (S-) diastereoisomers of methionine sulfoxide	-	-
Stress protein	-	ko:K05792
Prevents misfolding and promotes the refolding and proper assembly of unfolded polypeptides generated under stress conditions (groL2)	-	ko:K04077
Part of a stress-induced multi-chaperone system, it is involved in the recovery of the cell from heat-induced damage, in cooperation with DnaK, DnaJ and GrpE	AAA,AAA_2,ClpB_D2-small,Clp_N,UVR	ko:K03696
A protein kinase that phosphorylates Ser and Thr residues. Probably acts to suppress the effects of stress linked to accumulation of reactive oxygen species. Probably involved in the extracytoplasmic stress response		-
Part of a stress-induced multi-chaperone system, it is involved in the recovery of the cell from heat-induced damage, in cooperation with DnaK, DnaJ and GrpE (clpB)	AAA,AAA_2,ClpB_D2-small,Clp_N	ko:K03695,ko:K03696
May be involved in the stabilization of the cell envelope or may interact with the sensor protein CseC to modulate its activity, in response to cell envelope stress (cseA)		-
Prevents misfolding and promotes the refolding and proper assembly of unfolded polypeptides generated under stress conditions (gro2)	Cpn60_TCP1	ko:K04077
Stress protein (terE)	TerD	ko:K05795
(lepA) Required for accurate and efficient protein synthesis under certain stress conditions. May act as a fidelity factor of the translation reaction, by catalyzing a one-codon backward translocation of tRNAs on improperly translocated ribosomes. Back- translocation proceeds from a post-translocation (POST) complex to a pre-translocation (PRE) complex, thus giving elongation factor G a second chance to translocate the tRNAs correctly. Binds to ribosomes in a GTP-dependent manner	EFG_C,GTP_EFTU,GTP_EFTU_D2,LepA_C	ko:K03596
An essential GTPase which binds GTP, GDP and possibly (p)ppGpp with moderate affinity, with high nucleotide exchange rates and a fairly low GTP hydrolysis rate. Plays a role in control of the cell cycle, stress response, ribosome biogenesis and in those bacteria that undergo differentiation, in morphogenesis control (obg)	-	ko:K03979
Stress protein	-	ko:K05791
ATP-dependent serine protease that mediates the selective degradation of mutant and abnormal proteins as well as certain short-lived regulatory proteins. Required for cellular homeostasis and for survival from DNA damage and	-	ko:K01338

developmental changes induced (lon) by stress. Degrades polypeptides processively to yield small peptide fragments that are 5 to 10 amino acids long. Binds to DNA in a double-stranded, site-specific manner		
Stress protein	TerD	ko:K05795
Stress protein	TerD	ko:K05791,ko:K05795
Stress protein	-	ko:K05791
Part of a stress-induced multi-chaperone system, it is involved in the recovery of the cell from heat-induced damage, in cooperation with DnaK, DnaJ and GrpE	-	ko:K03696
**Ectoine**		
Catalyzes the circularization of gamma-N-acetyl- alpha,gamma-diaminobutyric acid (ADABA) to ectoine (1,4,5,6- tetrahydro-2-methyl-4-pyrimidine carboxylic acid), which is an excellent osmoprotectant (ectC)	Ectoine_synth	ko:K06720
ectoine hydroxylase (ectD)	Ectoine_synth	ko:K10674
Belongs to the class-III pyridoxal-phosphate-dependent aminotransferase family (ectB)	Aminotran_3	ko:K00836
L-2,4-diaminobutyric acid acetyltransferase (ectA)	Acetyltransf_1	ko:K06718
**Stress reduction: Proline**		
Belongs to the proline racemase family	Pro_racemase	ko:K01777
Catalyzes the reduction of 1-pyrroline-5-carboxylate (PCA) to L-proline (proC)	F420_oxidored,P5CR_dimer	ko:K00286
(proS) Catalyzes the attachment of proline to tRNA(Pro) in a two-step reaction proline is first activated by ATP to form Pro-AMP and then transferred to the acceptor end of tRNA(Pro). As ProRS can inadvertently accommodate and process non-cognate amino acids such as alanine and cysteine, to avoid such errors it has two additional distinct editing activities against alanine. One activity is designated as 'pretransfer' editing and involves the tRNA(Pro)-independent hydrolysis of activated Ala-AMP. The other activity is designated 'posttransfer' editing and involves deacylation of mischarged Ala-tRNA(Pro). The misacylated Cys- tRNA(Pro) is not edited by ProRS	HGTP_anticodon,tRNA-synt_2b,tRNA_edit	ko:K01881
Removes 5-oxoproline from various penultimate amino acid residues except L-proline (pcp)	Peptidase_C15	ko:K01304
**Sodium tolerance**		
Belongs to the sodium solute symporter (SSF) (TC 2.A.21) family	SSF	ko:K14393
Na( ) H( ) antiporter that extrudes sodium in exchange for external protons (nhaA)	Na_H_antiport_1	ko:K03313
F(1)F(0) ATP synthase produces ATP from ADP in the presence of a proton or sodium gradient. F-type ATPases consist of two structural domains, F(1) containing the extramembraneous catalytic core and F(0) containing the membrane proton channel, linked together by a central stalk and a peripheral stalk. During catalysis, ATP synthesis in the catalytic domain of F(1) is coupled via a rotary mechanism of the central stalk subunits to proton translocation (atpE)	ATP-synt_C	ko:K02110
PFAM sodium calcium exchanger	Na_Ca_ex	ko:K07301
Sodium/hydrogen exchanger family	-	-

**Stress reduction: ACC deaminase**		
1-aminocyclopropane-1-carboxylate deaminase	PALP(acds)	ko:K01505
**Stress reduction: catalase, peroxidase**		
Bifunctional enzyme with both catalase and broad- spectrum peroxidase activity (katG)	peroxidase	ko:K03782
Catalase activity	-	-
Antioxidant protein with alkyl hydroperoxidase activity. Required for the reduction of the AhpC active site cysteine residues and for the regeneration of the AhpC enzyme activity (ahpD)	CMD	ko:K04756
Peroxidase	Dyp_perox	ko:K15733
Peroxidase (efeN)	Dyp_perox	ko:K16301
Ferredoxin oxidoreductase	korA	ko:K00174
Ferredoxin oxidoreductase	korB	ko:K00175
Ferredoxin oxidoreductase	korA	ko:K00174
Ferredoxin	Fer4_13, Fer4_15	-
Ferredoxin	Fer4_13, Fer4_15	-
Ferredoxin	fdxA	-
ferredoxin	fdxG	ko:K05337
Ferredoxin	Fer4_13, Fer4_15	-
Pyruvate ferredoxin/flavodoxin oxidoreductase	POR	-
4Fe-4S single cluster domain of Ferredoxin I	Fer4_13	-
PFAM 4Fe-4S ferredoxin, iron-sulfur binding domain protein	Fer4_11,Fer4_3,Fer4_4,Fer4_7	ko:K00124
Flavodoxin reductases ferredoxin-NADPH reductases family 1	FAD_binding_6,Fer2, NAD_binding_1	-
Multimeric flavodoxin WrbA	FMN_red	-
Flavodoxin-like fold	FMN_red,Flavodoxin_2	-
Flavodoxin reductases ferredoxin-NADPH reductases family 1	FAD_binding_6,Fer2, Metal_hydrol,NAD_binding_1	
Thioredoxin	thiX	-
Thioredoxin	ybbN	ko:K05838
Thioredoxin	Thioredoxin_4	-
Belongs to the thioredoxin family	trxA	ko:K00384, ko:K03671
Belongs to the thioredoxin family	trxA	ko:K03671
Highly conserved protein containing a thioredoxin domain	GlcNAc_2-epim,Thioredox_DsbH	ko:K06888
Belongs to the thioredoxin family	SEC-C	-
DSBA-like thioredoxin domain	DSBA	-
Protein conserved in bacteria containing thioredoxin-like domain	Suc_Fer-like	-
Belongs to the thioredoxin family	trxA2	ko:K03671
Glutaredoxin	-	ko:K18917

Glutaredoxin	-	ko:K18917
PFAM Glutaredoxin 2	DUF836	-
**Stress reduction: Glutathione**		
Glutathione S-transferase (ygiG)	GST_C_2,GST_N_2	ko:K07393
Belongs to the glutathione peroxidase family (btuE)	GSHPx	ko:K00432
**Biodegradation enzyme: Amylase**		
Alpha amylase, catalytic	Alpha-amylase,DUF3459	ko:K01187
**Biodegradation enzyme: cellulase**		
Belongs to the glycosyl hydrolase 5 (cellulase A) family	-	-
Belongs to the glycosyl hydrolase 11 (cellulase G) family (xlnB)	CBM_2,Glyco_hydro_10,Ricin_B_lectin	ko:K01181
Belongs to the glycosyl hydrolase 12 (cellulase H) family (celA1)	ko:K01179	ko:K01179
**Biodegradation enzyme:Xylose isomerase**		
Belongs to the xylose isomerase family (xylA)	AP_endonuc_2	ko:K01805
PFAM Xylose isomerase domain protein TIM barrel	-	-
Xylose isomerase-like TIM barrel	-	-
Acetyl xylan esterase	AXE1	ko:K01060
**Biodegradation enzyme: pectin hydrolysis**	-	-
Pectin esterase	Pectin esterase	ko:K01051
**Biodegradation enzyme: lipase**		
Lipase (class 3)	Lipase_3	ko:K01046
**PGPB: Phosphatase**		
Alkaline phosphatase (phoD)	PhoD,PhoD_N	ko:K01113
SMART protein phosphatase 2C domain protein (prpM4)	-	-
**PGPB: Siderophore production**		
Siderophore biosynthesis protein domain	-	-
siderophore-interacting protein (viuB)	-	-
siderophore-interacting protein (sidE)	-	-
**PGPB: IAA production**		
The alpha subunit is responsible for the aldol cleavage of indoleglycerol phosphate to indole and glyceraldehyde 3-phosphate (trpA)	Trp_syntA	ko:K01695
The beta subunit is responsible for the synthesis of L-tryptophan from indole and L-serine (trpB)	PALP	ko:K01696
**Phenazine production**		
Phenazine biosynthesis protein PhzF (phZC)	-	-
Catalyzes the Claisen rearrangement of chorismate to prephenate	CM_2	ko:K04093
Phenazine biosynthesis PhzC PhzF protein (phzF)	-	-
Phenazine biosynthesis-like protein (phzF)	-	-
**Other: phytoene**		
Phytoene synthase (crtB)	SQS_PSY	ko:K02291

Squalene phytoene synthase (hopE)	-	-
Phytoene (crtl2)	Amino_oxidase	ko:K01854
**Other: riboflavin**		
Pyrimidine reductase, riboflavin biosynthesis	RibD_C	ko:K00082
Catalyzes the formation of 6,7-dimethyl-8- ribityllumazine by condensation of 5-amino-6-(D- ribitylamino)uracil with 3,4-dihydroxy-2-butanone 4-phosphate. This is the penultimate step in the biosynthesis of riboflavin (ribH)	DMRL_synthase	ko:K00794
Catalyzes the conversion of D-ribulose 5-phosphate to formate and 3,4-dihydroxy-2-butanone 4-phosphate (ribBA)	DHBP_synthase,GTP_cyclohydro2	ko:K14652
riboflavin synthase, alpha (ribE)	Lum_binding	ko:K00793
**Exopolysaccharide production**		
Exopolysaccharide biosynthesis protein related to N-acetylglucosamine-1-phosphodiester alpha-N-acetylglucosaminidase	NAGPA	-

Table S11. Gene prediction for gene annotation of the genome of *Micrococcus* strain EWR3.9.1 relating to stress reduction and plant growth promoting proteins, biodegradation enzymes, and other compounds. PFAM: Protein Families Database; Ko: KEGG Orthology; KEGG: Kyoto Encyclopedia of Genes and Genomes.

Description	PFAM	KEGG_Ko
**Stress reduction: Glycine-betaine, proline**		
Involved in the biosynthesis of the osmoprotectant glycine betaine. Catalyzes the oxidation of choline to betaine aldehyde and betaine aldehyde to glycine betaine at the same rate.	GMC_oxred_C,GMC_oxred_N	ko:K00108,ko:K17755
Substrate binding domain of ABC-type glycine betaine transport system	OpuAC	ko:K05845
Involved in the biosynthesis of the osmoprotectant glycine betaine. Catalyzes the oxidation of choline to betaine aldehyde and betaine aldehyde to glycine betaine at the same rate	betA	ko:K00108,ko:K17755
Catalyzes the reduction of 1-pyrroline-5-carboxylate (PCA) to L-proline	F420_oxidored,P5CR_dimer	ko:K00286
**Stress reduction: heat and osmotic pressure**		
Channel that opens in response to stretch forces in the membrane lipid bilayer. May participate in the regulation of osmotic pressure changes within the cell	MscL	ko:K03282
Participates actively in the response to hyperosmotic and heat shock by preventing the aggregation of stress-denatured proteins, in association with DnaK and GrpE. It is the nucleotide exchange factor for DnaK and may function as a thermosensor. Unfolded proteins bind initially to DnaJ	GrpE	ko:K03687
Heat shock 70 kDa protein	HSP70	ko:K04043
Channel that opens in response to stretch forces in the membrane lipid bilayer. May participate in the regulation of osmotic pressure changes within the cell	MscL	ko:K03282
Universal stress protein family	Usp	-
Response regulators are key elements in two-component signal transduction systems, which enable bacteria to sense, respond, and adapt to a wide range of environments, stressors, and growth conditions	tcsR2	-
Prevents misfolding and promotes the refolding and proper assembly of unfolded polypeptides generated under stress conditions	groL	ko:K04077
Prevents misfolding and promotes the refolding and proper assembly of unfolded polypeptides generated under stress conditions	groL2	ko:K04077
Part of a stress-induced multi-chaperone system, it is involved in the recovery of the cell from heat-induced damage, in cooperation with DnaK, DnaJ and GrpE	clpB	ko:K03695,ko:K03696
Heat shock 70 kDa protein	dnaK	ko:K04043
Ectoine synthase	ectC	ko:K06720
**Stress: catalase, peroxidase**		
Serves to protect cells from the toxic effects of hydrogen peroxide	Catalase, Catalase-rel	ko:K03781

Dyp-type peroxidase family	-	ko:K15733
PFAM alkyl hydroperoxide reductase Thiol specific antioxidant Mal allergen	bcp	ko:K03564
Manganese containing catalase	-	ko:K07217
Belongs to the catalase family	katA	ko:K03781
**Stress reduction: Antioxidant**		
Ferredoxin	-	ko:K00230
Flavodoxin domain	pknE	-
Thioredoxin	-	ko:K03672
Thioredoxin	AhpC-TSA,NHL,Thioredoxin_8	-
Thioredoxin-like	trxA	ko:K03671
Belongs to the thioredoxin family	trxA	ko:K03671
Belongs to the thioredoxin family	trxA	ko:K00384,ko:K03671
Belongs to the thioredoxin family	Thioredoxin_4	-
DSBA-like thioredoxin domain	nrdH	ko:K06191
Glutaredoxin	-	ko:K18917
Glutaredoxin	DUF836	-
Glutaredoxin-like domain (DUF836)	resA	-
Redoxin	ahpE	ko:K03386
Redoxin	-	ko:K00230
**Defense mechanism: antitoxin**		
Natural resistance-associated macrophage protein	Nramp	-
Antitoxin component of a toxin-antitoxin (TA) module	relJ	ko:K19159
PemK-like, MazF-like toxin of type II toxin-antitoxin system	-	-
YoeB-like toxin of bacterial type II toxin-antitoxin system	yoeB	ko:K19158
**Other stress tolerance**		
Copper resistance	-	ko:K02351,ko:K07245
Cadmium resistance transporter	cad	-
Subunit A of antiporter complex involved in resistance to high concentrations of Na , K , Li and or alkali	mrpA/mrpB	ko:K05565,ko:K14086
**Biodegradation enzyme: Amylase**		
PFAM alpha amylase, catalytic	Alpha-amylase,DUF3459	ko:K01187
**Biodegradation enzyme: xylose isomerase**		
Xylose isomerase-like TIM barrel	AP_endonuc_2	
**Biodegradation enzyme: Lipase**		
GDSL-like Lipase/Acylhydrolase	Lipase_GDSL_2	
GDSL-like Lipase/Acylhydrolase family	Lipase_GDSL_2	
**Plant growth promoting; phosphatase**		
Low molecular weight phosphatase family	HTH_5,LMWPc	ko:K03892
Ppx/GppA phosphatase family	Ppx-GppA	ko:K01524
PhoD-like phosphatase	PhoD,PhoD_N	ko:K01113

**Plant growth promoting: siderophore production**		
Siderophore-interacting protein	FAD_binding_9,SIP	
Siderophore-interacting protein	FAD_binding_9,SIP	
**Plant growth promoting: Indole 3 acetic acid production**		
The alpha subunit is responsible for the aldol cleavage of indoleglycerol phosphate to indole and glyceraldehyde 3-phosphate	Trp_syntA	ko:K01695
**Other compounds**		
L-asparaginase II	Asparaginase_II	
Riboflavin synthase, alpha subunit	Lum_binding	ko:K00793
Phytoene synthase	SQS_PSY	ko:K02291
Lycopene cyclase domain		
Lycopene cyclase		
Siroheme synthase	NAD_binding_7,TP_methylase	ko:K02302,ko:K02303
Spermidine synthase	speE	ko:K00797
Cellulose biosynthesis protein BcsQ	soj	ko:K03496
Colicin V production protein	cvpA	-
Cobalamin-independent synthase, Catalytic domain	metE	ko:K00549,ko:K22363

Table S12. GenBank accession numbers of all *Streptomyces* and non-*Streptomyces* strains, and non-actinobacteria strains sequenced in the study.

Genus	strain	GenBank accession number	Genus	strain	GenBank accession number
Streptomyces sp.	EBS5.2	PX398345	**Non-*Streptomyces* genera**		
Streptomyces sp.	EB1.5	PX398346	*Peterkaempfera*	ESS3.1	PX399520
Streptomyces sp.	EBL7.9	PX398347	*Peterkaempfera*	ESS2.4	PX399521
Streptomyces sp.	EBR3.2	PX398348	*Brachybacterium*	ESR6.10	PX399522
Streptomyces sp.	EBR3.8	PX398349	*Brevibacterium*	ESR5.3	PX399523
Streptomyces sp.	EBR4.16	PX398350	*Cellulosimicrobium*	EWS3.12	PX399524
Streptomyces sp.	EBR7.15	PX398351	*Curtobacterium*	EWL6.1	PX399525
Streptomyces sp.	EBS5.1	PX398352	*Gordonia*	EKL3.2	PX399526
Streptomyces sp.	EBS5.3	PX398353	*Kocuria*	EBL3.4	PX399527
Streptomyces sp.	EBS7.9	PX398354	*Kocuria*	EBAL27	PX399528
Streptomyces sp.	ECL5.16	PX398355	*Kocuria*	EWS3.8.2	PX399529
Streptomyces sp.	ECL5.20	PX398356	*Microbacterium*	EKS6.13	PX399530
Streptomyces sp.	ECL6.4	PX398357	*Microbacterium*	EKS8.26	PX399531
Streptomyces sp.	ECL7.10	PX398358	*Micrococcus*	EWR3.9.1	PX399532
Streptomyces sp.	ECL7.29	PX398359	*Tsukamurella*	EKL2.3	PX399533
Streptomyces sp.	ECL8.1	PX398360	*Tsukamurella*	EWR2.8	PX399534
Streptomyces sp.	ECL8.19	PX398361	**Non-Actinobacteria**		
Streptomyces sp.	ECN6	PX398362	*Aureimonas*	EWS3.8A	PX399489
Streptomyces sp.	ECR2.10	PX398363	*Bacillus*	EKR6.16	PX399490
Streptomyces sp.	ECR3.25	PX398364	*Bacillus*	EWS8.13	PX399491
Streptomyces sp.	ECR3.3	PX398365	*Bacillus*	ESR3.34	PX399492
Streptomyces sp.	ECR3.35	PX398366	*Chryseobacterium*	EBS5.19	PX399493
Streptomyces sp.	ECR3.81	PX398367	*Chryseobacterium*	EBR3.16	PX399494
Streptomyces sp.	ECR5.32	PX398368	*Chryseobacterium*	EKL5.7	PX399495
Streptomyces sp.	ECR5.7	PX398369	*Deinococcus*	EKL3.2.1	PX399496
Streptomyces sp.	ECR7.1	PX398370	*Deinococcus*	EWSD8.20	PX399497
Streptomyces sp.	ECS5.14	PX398371	*Massilia*	EWR1.21	PX399498
Streptomyces sp.	ECS6.12	PX398372	*Massilia*	EWR3.23	PX399499
Streptomyces sp.	ECS7.17	PX398373	*Massilia*	ESR4.26	PX399500
Streptomyces sp.	ECS7.22	PX398374	*Methylobacterium*	EKL4.21	PX399501
Streptomyces sp.	EK7.15	PX398375	*Methylobacterium*	ESS3.12	PX399502
Streptomyces sp.	EKL1.1	PX398376	*Methylobacterium*	EWS3.8	PX399503
Streptomyces sp.	EKL5.11	PX398377	*Pseudomonas*	EBR8.6	PX399504
Streptomyces sp.	EKL6.13	PX398378	*Pseudomonas*	EBR8.5	PX399505

Genus	strain	GenBank accession number	Genus	strain	GenBank accession number
Streptomyces sp.	EKL7.21	PX398379	*Pseudomonas*	EKR5.15	PX399506
Streptomyces sp.	EKR1.2	PX398380	*Pseudomonas*	EWR2.3	PX399507
Streptomyces sp.	EKR5.2	PX398381	*Pseudomonas*	EWR2.6	PX399508
Streptomyces sp.	EKR6.13	PX398382	*Pseudomonas*	EWR8.11	PX399509
Streptomyces sp.	EKR6.15	PX398383	*Pseudomonas*	ESS2.5	PX399510
Streptomyces sp.	EKR7.5	PX398384	*Serratia*	EBR3.8S	PX399511
Streptomyces sp.	EKS3.12	PX398385	*Serratia*	EKL4.22	PX399512
Streptomyces sp.	EKS3.5	PX398386	*Serratia*	EKR6.9	PX399513
Streptomyces sp.	EKS4.1	PX398387	*Serratia*	EKS4.13	PX399514
Streptomyces sp.	EKS5.11	PX398388	*Staphylococcus*	EKL4.16	PX399515
Streptomyces sp.	EKS8.28	PX398389	*Staphylococcus*	EWR6.6	PX399516
Streptomyces sp.	ESL1.1	PX398390	*Stenotrophomonas*	EKL7.20	PX399517
Streptomyces sp.	ESL2.7	PX398391			
Streptomyces sp.	ESL3.1	PX398392			
Streptomyces sp.	ESL3.11	PX398393			
Streptomyces sp.	ESL4.13	PX398394			
Streptomyces sp.	ESL4.15	PX398395			
Streptomyces sp.	ESL5.5	PX398396			
Streptomyces sp.	ESL6.5	PX398397			
Streptomyces sp.	ESL7.2	PX398398			
Streptomyces sp.	ESL8.11	PX398399			
Streptomyces sp.	ESR1.3	PX398400			
Streptomyces sp.	ESR1.8	PX398401			
Streptomyces sp.	ESR2.15	PX398402			
Streptomyces sp.	ESR3.25	PX398403			
Streptomyces sp.	ESR3.26	PX398404			
Streptomyces sp.	ESR3.38	PX398405			
Streptomyces sp.	ESR3.4	PX398406			
Streptomyces sp.	ESR4.19	PX398407			
Streptomyces sp.	ESR4.20	PX398408			
Streptomyces sp.	ESR5.15	PX398409			
Streptomyces sp.	ESR5.16	PX398410			
Streptomyces sp.	ESR5.20	PX398411			
Streptomyces sp.	ESR5.40	PX398412			
Streptomyces sp.	ESR6.16	PX398413			
Streptomyces sp.	ESR7.3	PX398414			
Streptomyces sp.	ESS2.7	PX398415			
Streptomyces sp.	ESS3.11	PX398416			

**Genus**	**strain**	**GenBank accession number**	**Genus**	**strain**	**GenBank accession number**
Streptomyces sp.	ESS3.9	PX398417			
Streptomyces sp.	ESS5.2	PX398418			
Streptomyces sp.	ESS5.21	PX398419			
Streptomyces sp.	ESS7.22	PX398420			
Streptomyces sp.	ESS7.7	PX398421			
Streptomyces sp.	ESS7.8	PX398422			
Streptomyces sp.	ESS8.20	PX398423			
Streptomyces sp.	EWL3.20	PX398424			
Streptomyces sp.	EWL3.9	PX398425			
Streptomyces sp.	EWL5.16	PX398426			
Streptomyces sp.	EWL6.4	PX398427			
Streptomyces sp.	EWL8.17	PX398428			
Streptomyces sp.	EWR1.13	PX398429			
Streptomyces sp.	EWR1.23	PX398430			
Streptomyces sp.	EWR1.4	PX398431			
Streptomyces sp.	EWR6.5	PX398432			
Streptomyces sp.	EWR6.5.1	PX398433			
Streptomyces sp.	EWR7.15	PX398434			
Streptomyces sp.	EWR7.9	PX398435			
Streptomyces sp.	EWR8.17	PX398436			
Streptomyces sp.	EWRD8.25	PX398437			
Streptomyces sp.	EWS1.12	PX398438			
Streptomyces sp.	EWS3.1	PX398439			
Streptomyces sp.	EWS3.17.1	PX398440			
Streptomyces sp.	EWS5.2	PX398441			
Streptomyces sp.	EWS5.6	PX398442			
Streptomyces sp.	EWS6.1	PX398443			
Streptomyces sp.	EWS6.11	PX398444			
Streptomyces sp.	EWS8.8	PX398445			
