# Supplementary material for: Phylogenetic affiliation of endophytic actinobacteria associated with red gum tree grown in salinity area and their plant growth promoting properties and suppression of phytopathogens, and genome data mining of selected strains
Source: Front Plant Sci. 2025 Nov 26;16:1610327. doi: 10.3389/fpls.2025.1610327 (PMC12689991; doi:10.3389/fpls.2025.1610327)
Supplement: Supplementary file 2 [file DataSheet2.pdf]

Supplementary Figures

Phylogenetic affiliation of endophytic actinobacteria associated with red gum tree grown in salinity area and their plant growth promoting properties and suppression of phytopathogens, and genome data mining of selected strains

Onuma Kaewkla[1,2*], Kawintip Kiakhunthod[1,2], Sumalee Chookhampaeng[1], Busayarat Klinjantasorn[1,2], Piriya Klankeo[3], Winya Dungkaew[2]

Sample EK; GPS; 16.39910º N, 103.27090º E; Plant height 170*3 centimeters; Plant diameter 7 inch

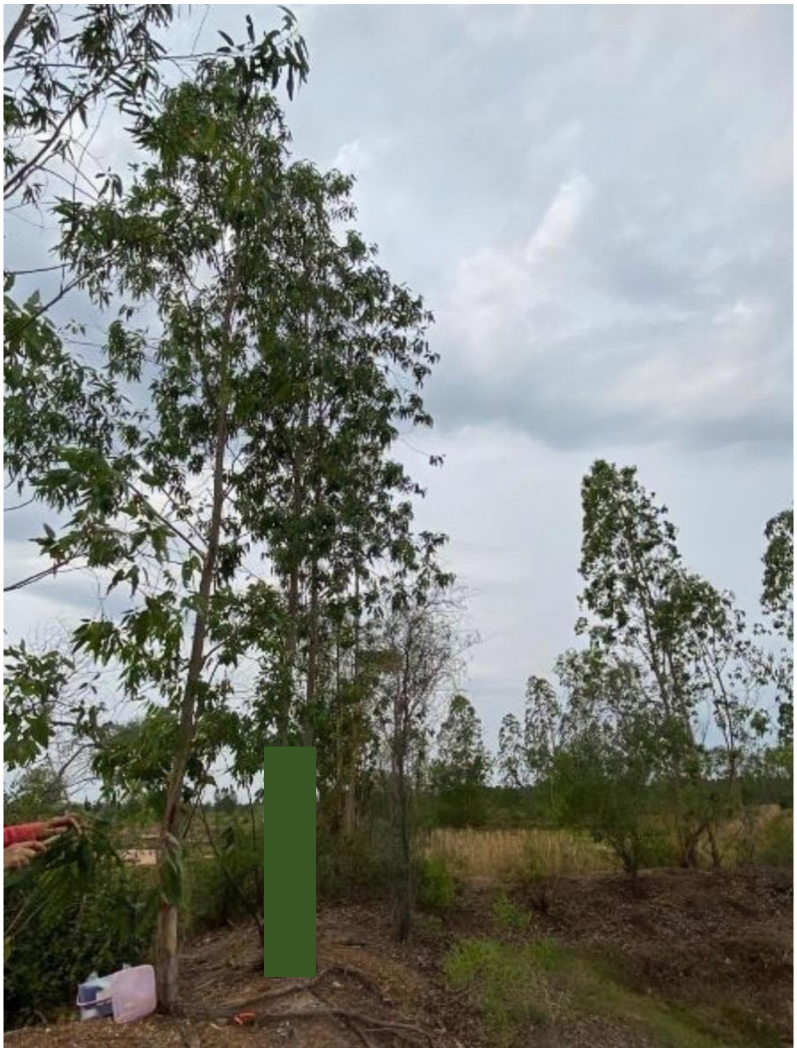

A)

Sample EC; GPS; 16.40384º N, 103.26985º E; Plant height 200 centimeters; Plant diameter 2 inch

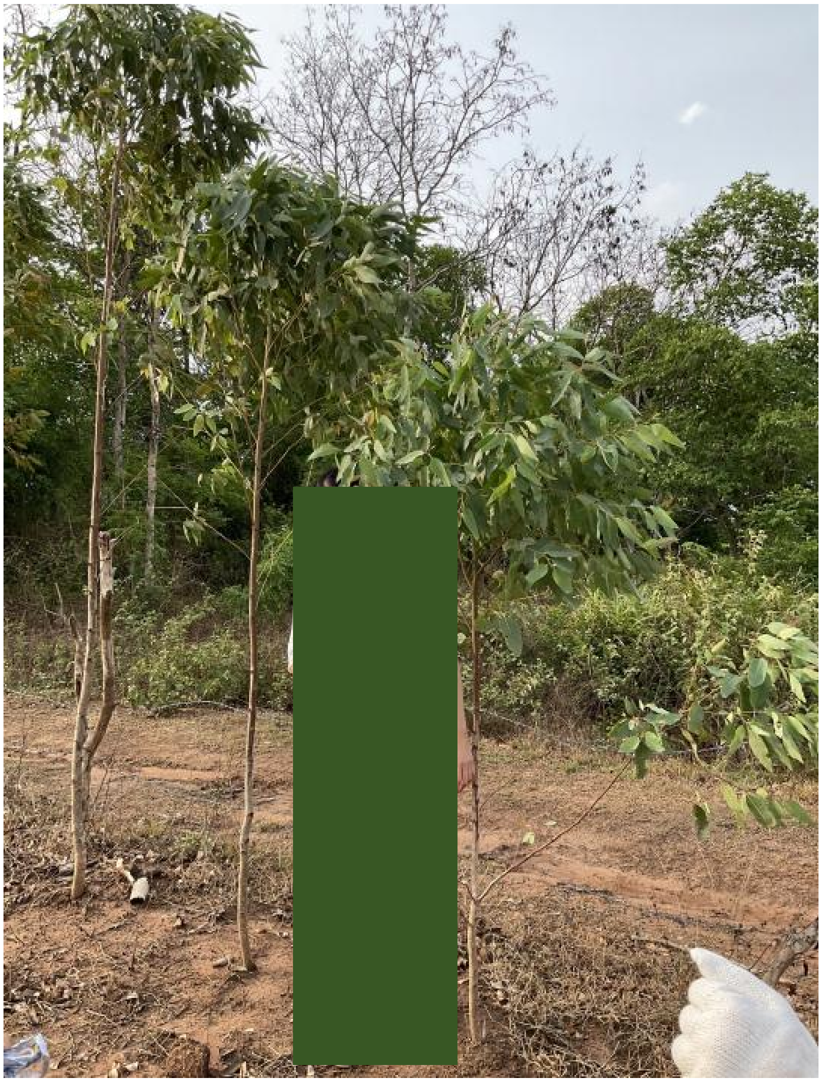

B)

Sample EB; GPS; 16.40703º N, 103.27121º E; Plant height 170*4 centimeters
Plant diameter 14.5 inch

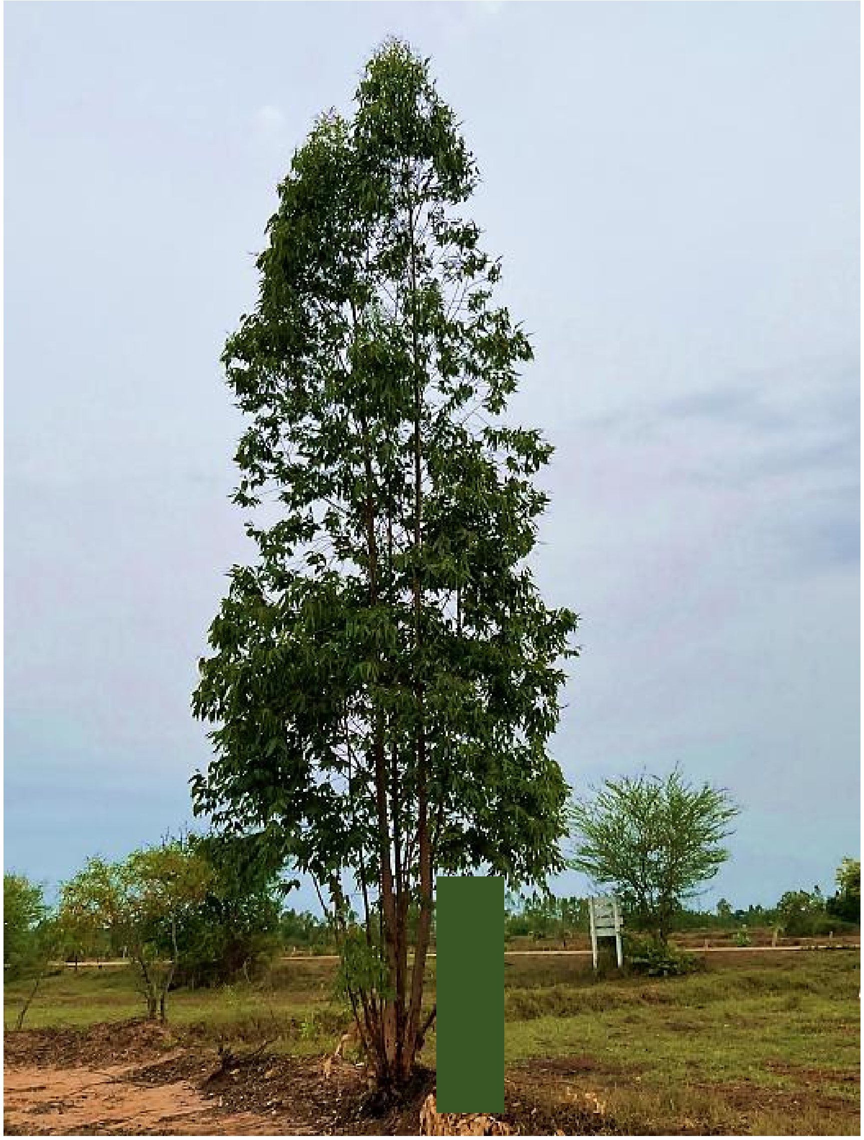

C)

Sample EW; GPS; 16.40480º N, 103.28081º E; Plant height 170*4 centimeters; Plant diameter 16 inch

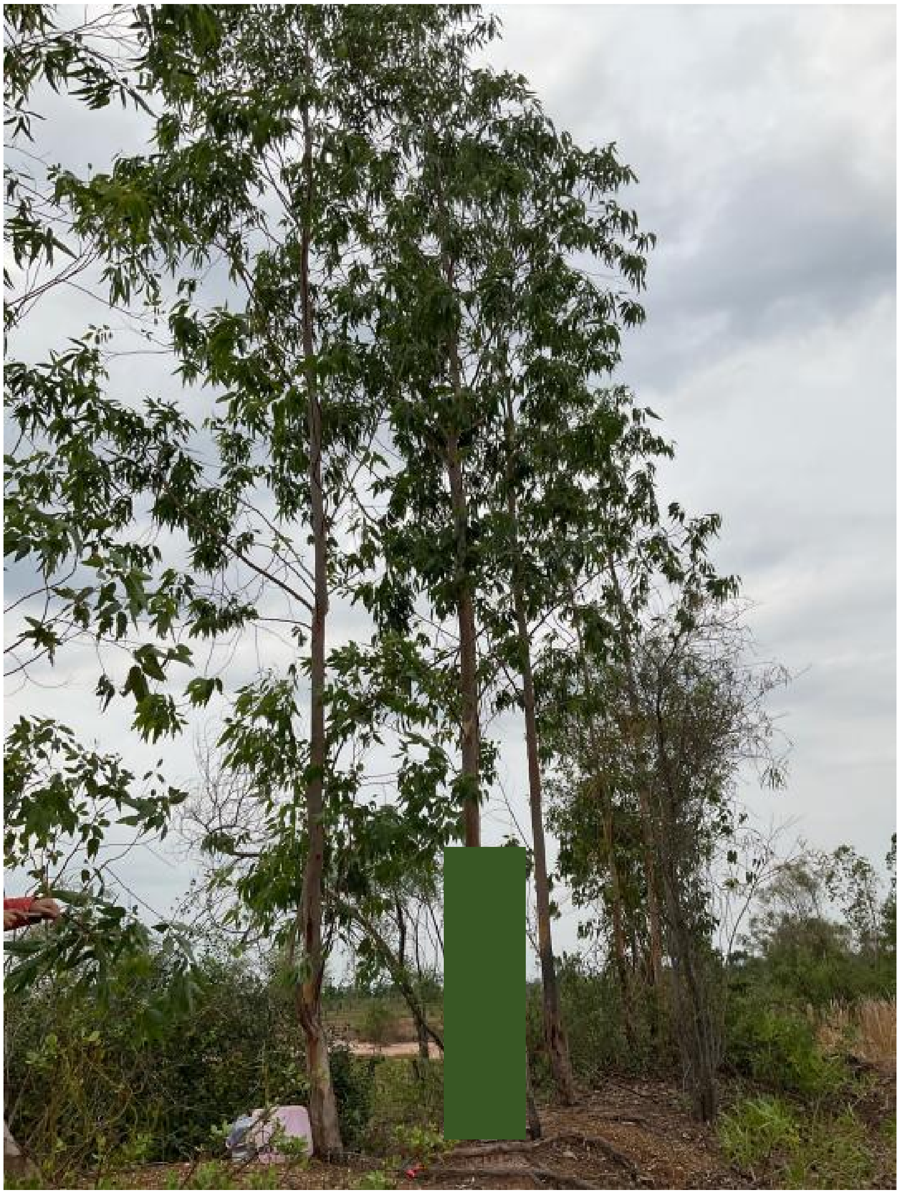

D)

Sample ES; GPS; 16.40720º N, 103.27136º E; Plant height 170*4 centimeters;
Plant diameter 19 inch

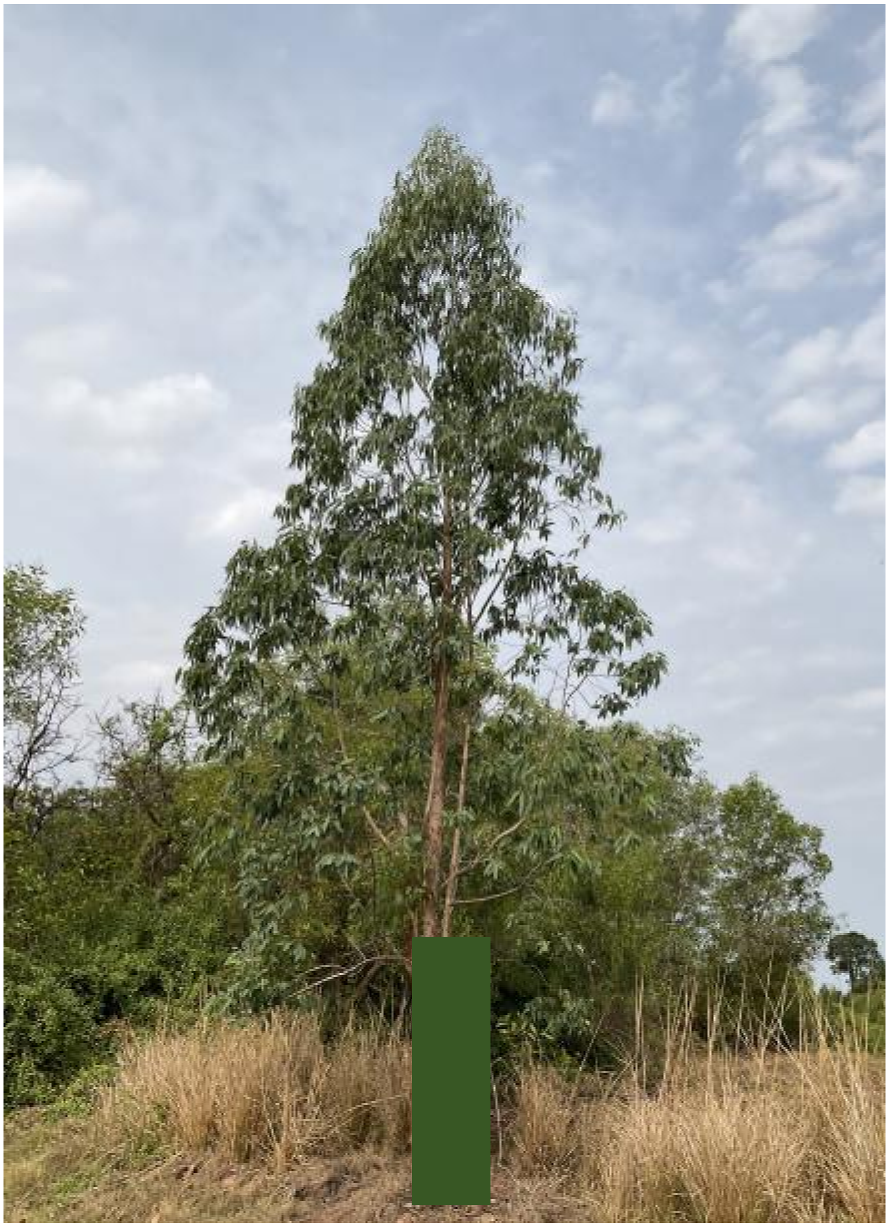

E)

Figure S1. Five *Eucalyptus camaldulensis* plant samples collected in the different locations in Ponsim Village, Tumbol Hua Nakham, Yang Ta-lat District, Kalasin Province, Thailand.

A) EK sample, B) EC sample, C) EB sample, D) EW sample, and E) ES sample. The green-colored rectangular block shows the scale of a person who is 170 cm tall.

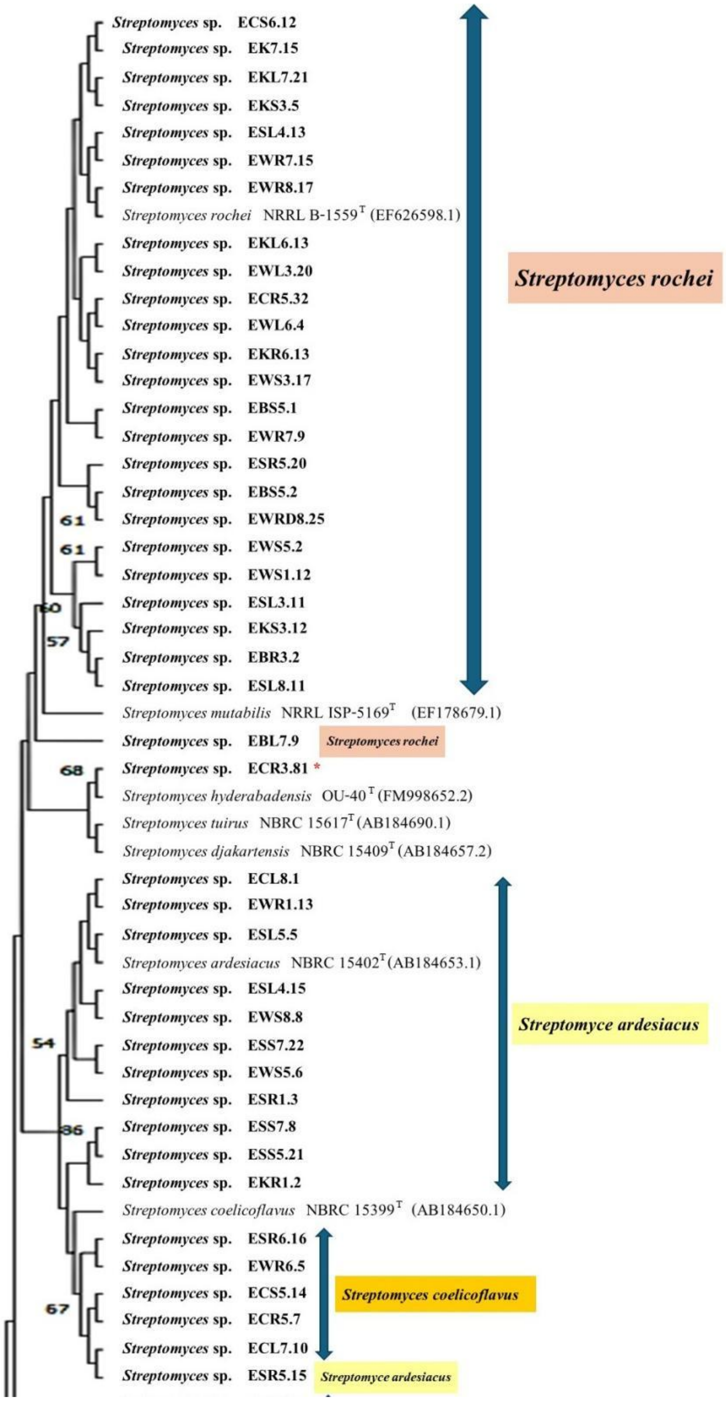

Figure S2. The maximum likelihood phylogenetic tree is based on 16S rRNA gene sequences of 101 strains of *Streptomyces* and 2 strains of the genus *Peterkaempfera* and their closely related members in the genera *Streptomyces* and *Peterkaempfera* and *Embleya scabrisporus* DSM 41855^T as the out-group. Bootstrap values based on 1000 replicates are shown at the branch nodes. *; indicate a single strain of a unique species of *Streptomyces.*

Figure S2 continued

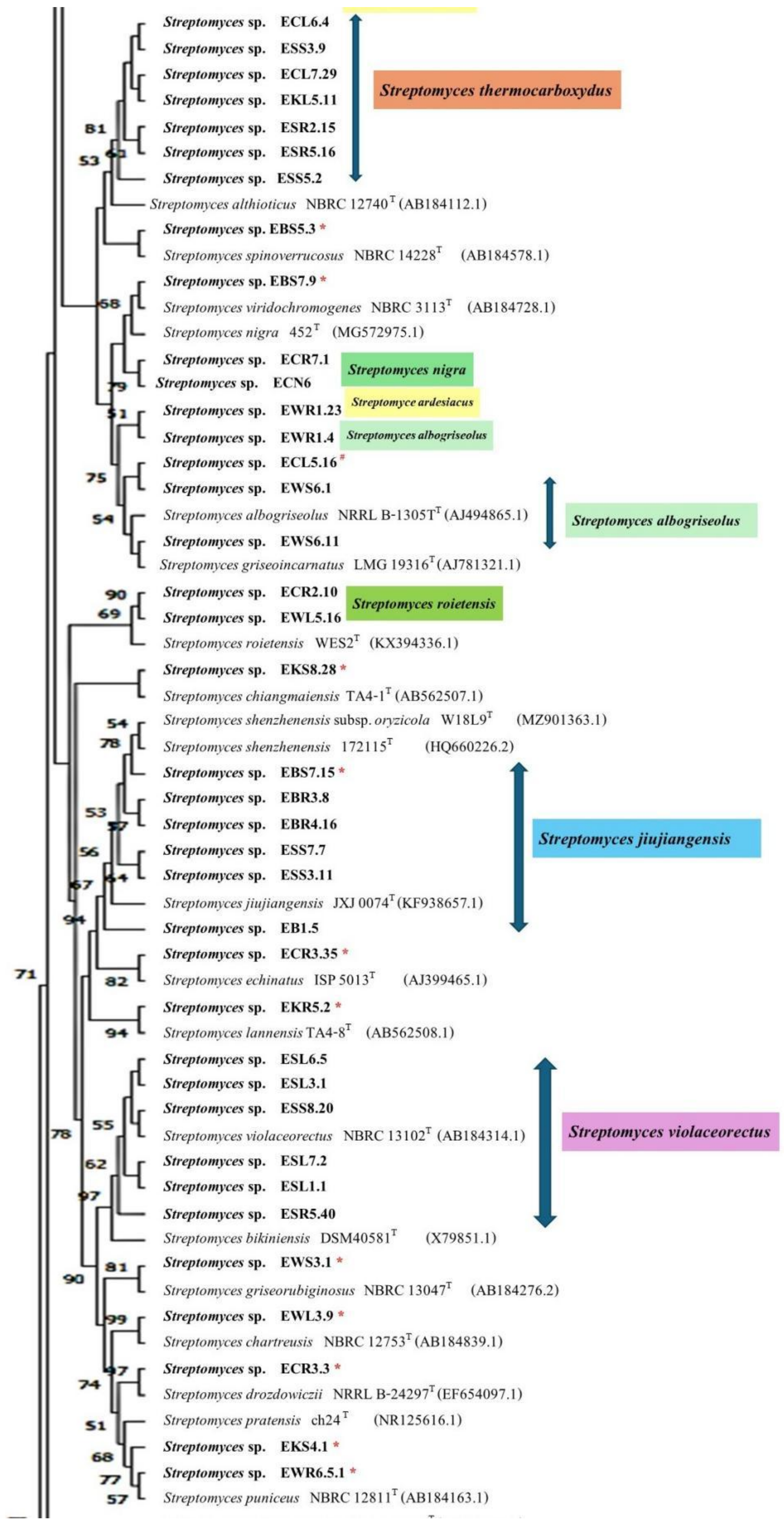

Figure S2 Continued

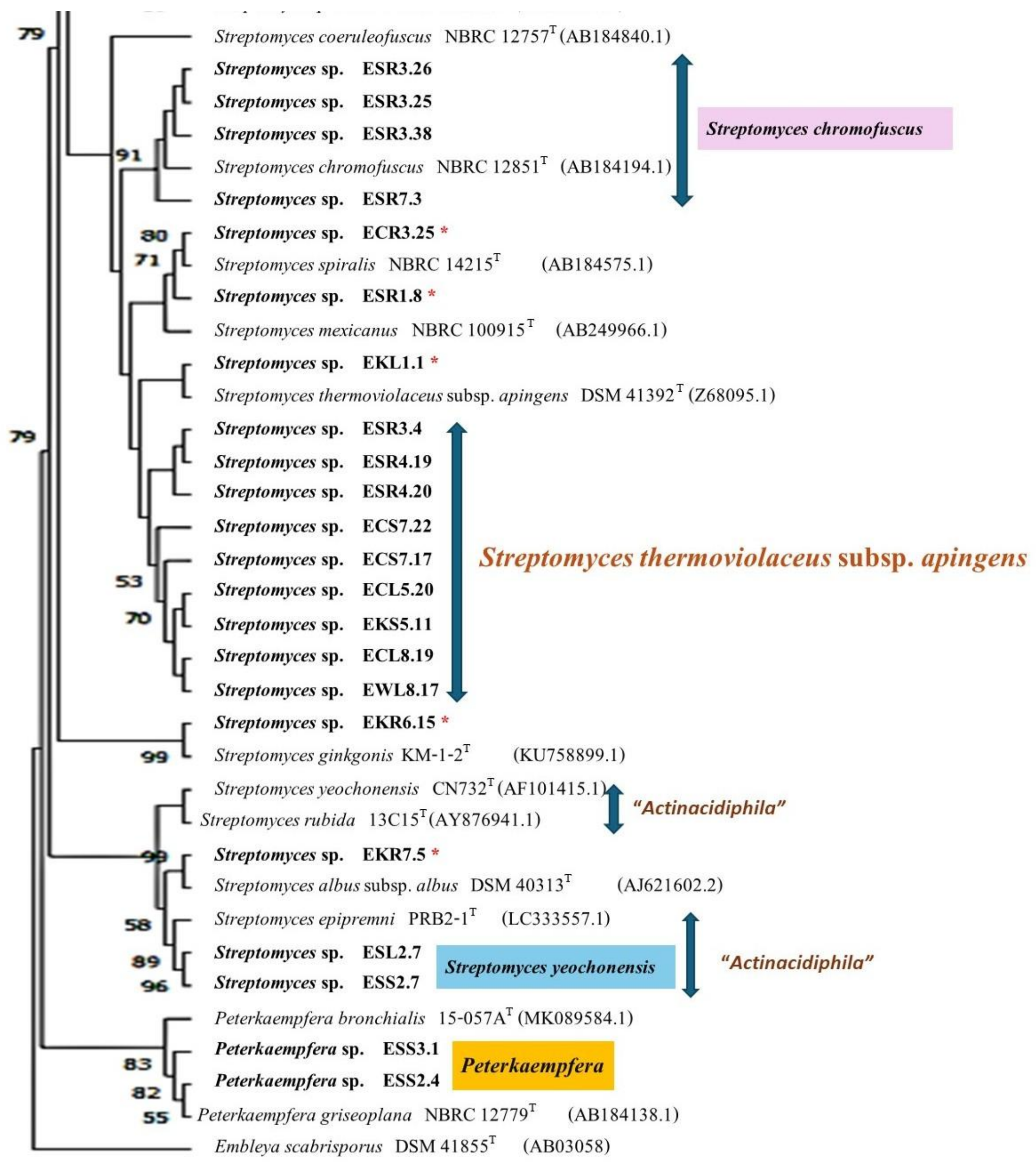

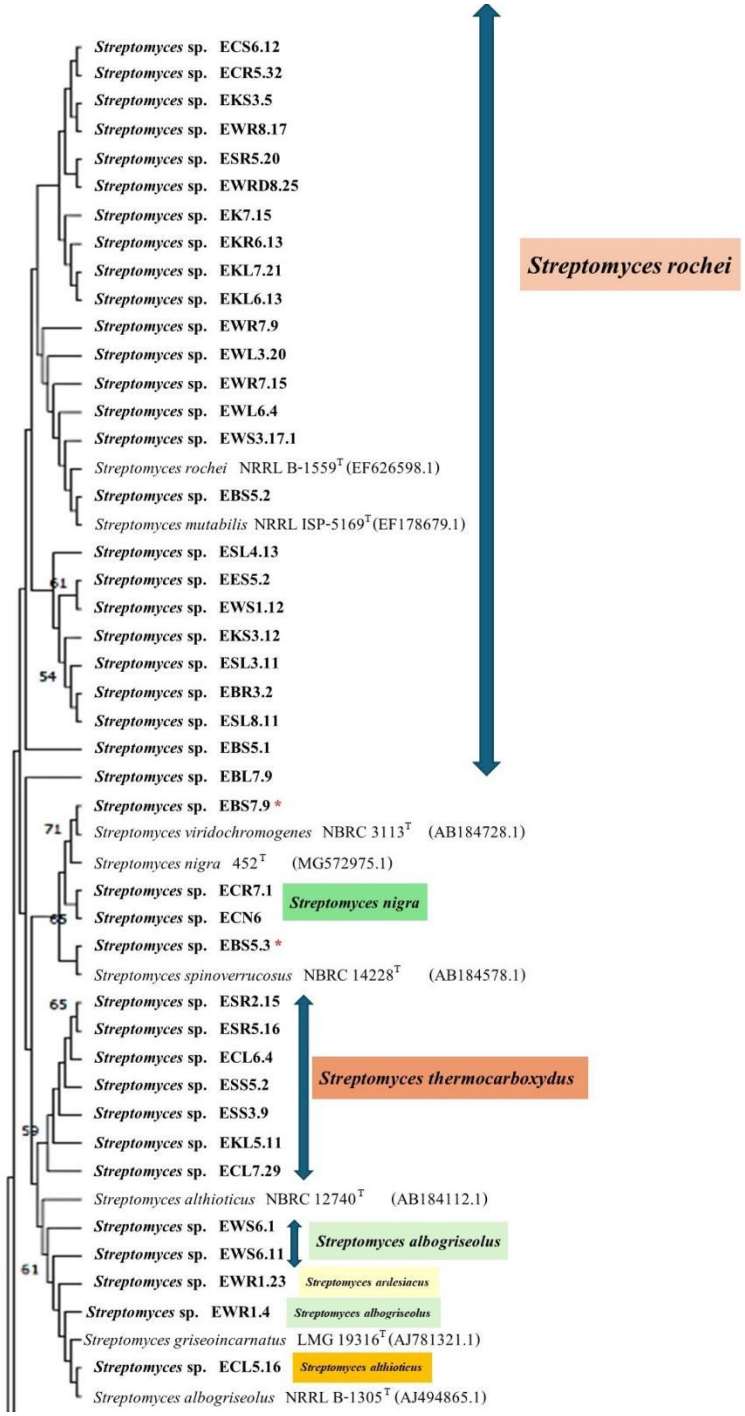

Figure S3. The maximum parsimony phylogenetic tree is based on 16S rRNA gene sequences of 101 strains of *Streptomyces* and 2 strains of the genus *Peterkaempfera* and their closely related members in the genera *Streptomyces* and *Peterkaempfera* and *Embleya scabrisporus* DSM 41855^{T} as the out-group. Bootstrap values based on 1000 replicates are shown at the branch nodes. *; indicate a single strain of a unique species of *Streptomyces.*

Figure S3 Continued

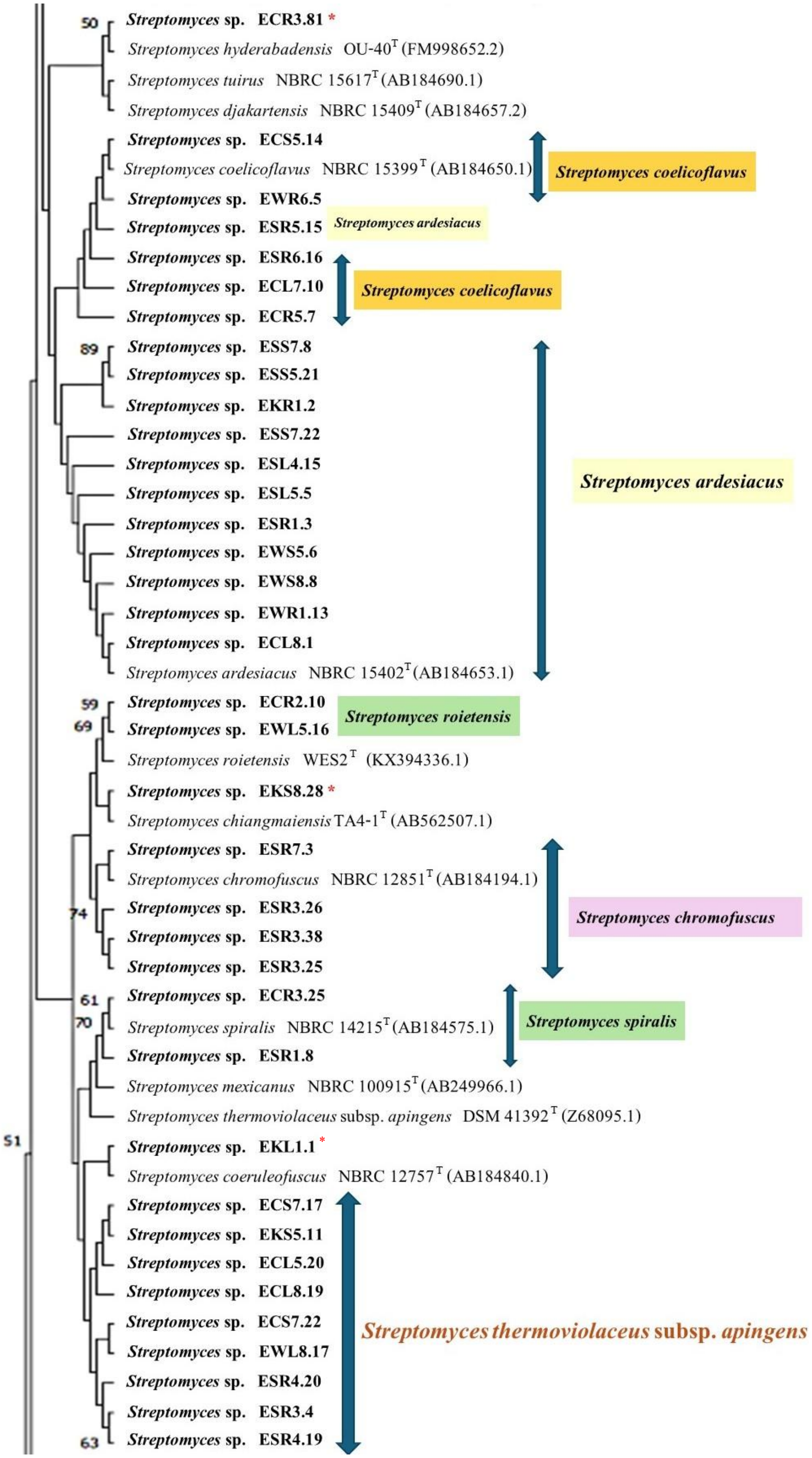

Figure S3 Continued

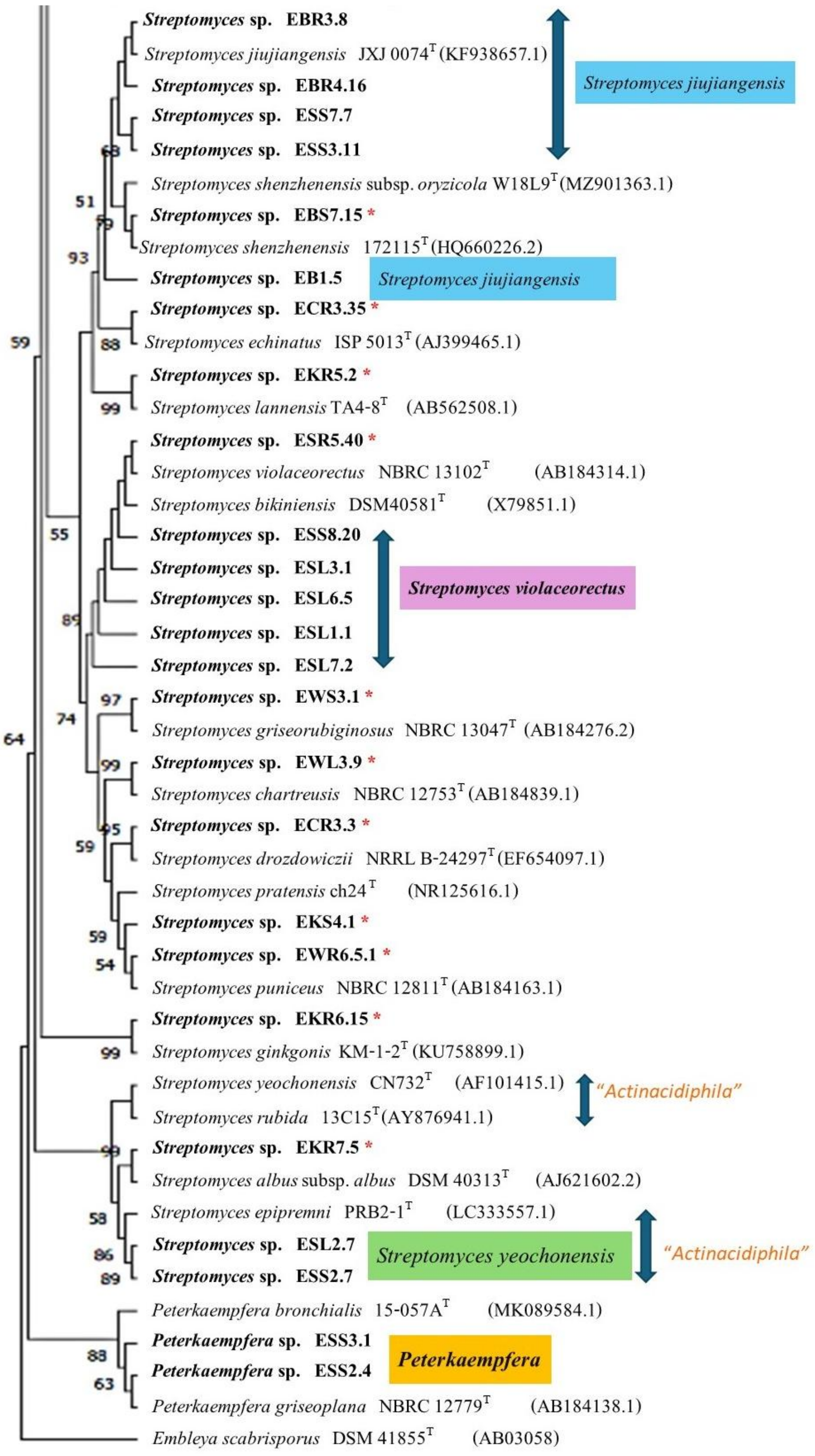

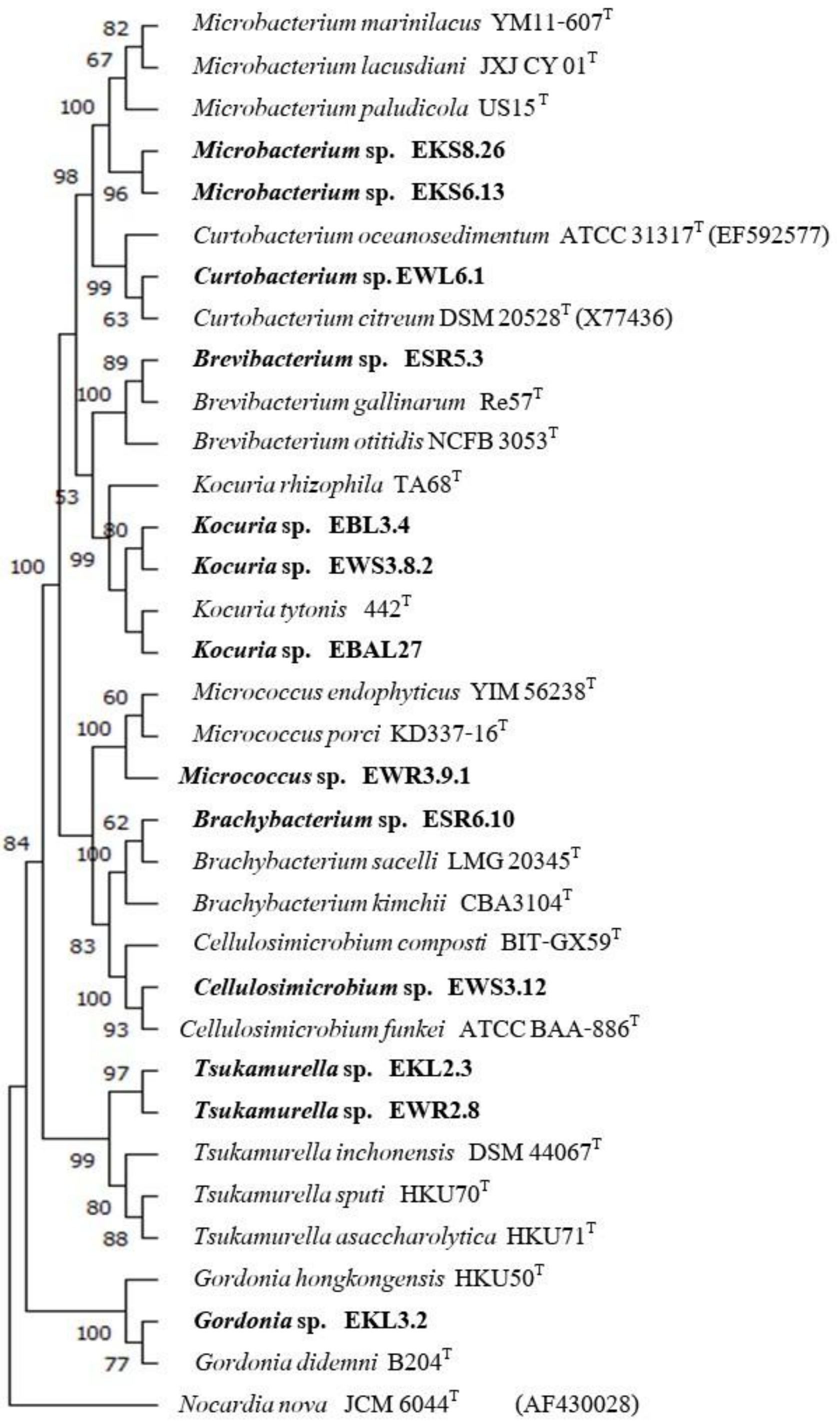

Figure S4. The maximum likelihood phylogenetic tree is based on the 16S rRNA gene sequences of 13 strains from 9 genera and their closely related members, with *Nocardia nova* JCM6044^T serving as the out-group. Bootstrap values based on 1000 replicates are shown at the branch nodes.

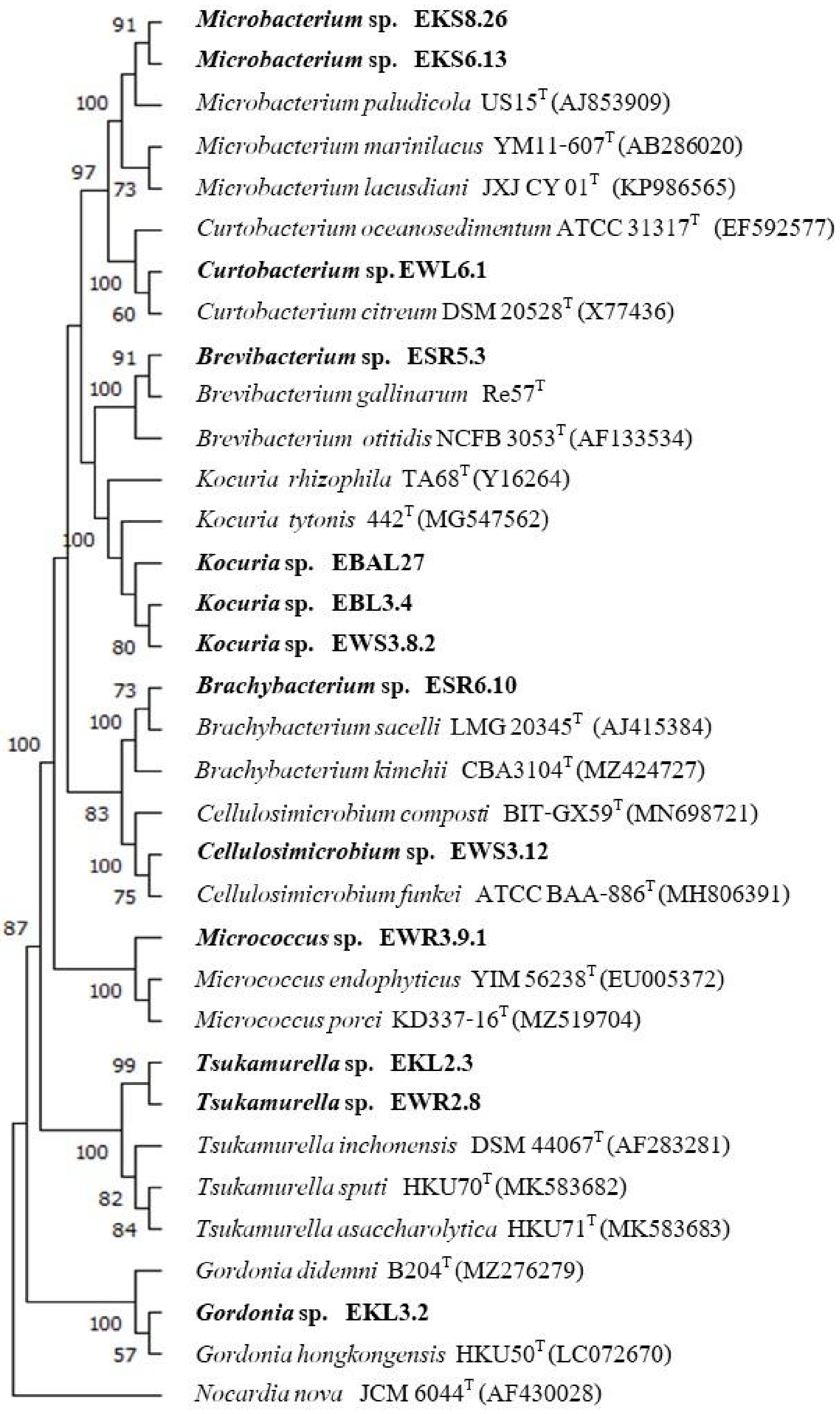

Figure S5. The maximum parsimony phylogenetic tree is based on the 16S rRNA gene sequences of 13 strains from 9 genera and their closely related members, with *Nocardia nova* JCM6044^T serving as the out-group. Bootstrap values based on 1000 replicates are shown at the branch nodes.

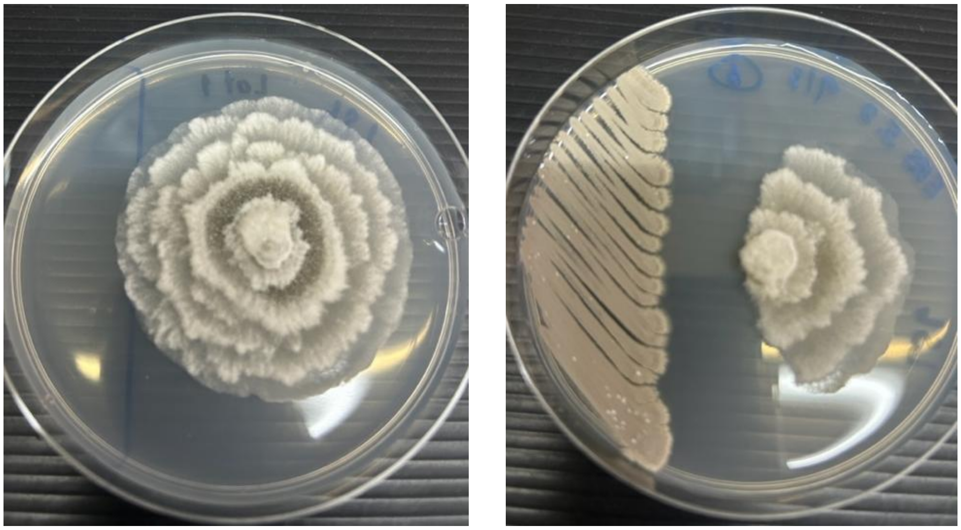

A B

Figure S6. The fungal inhibition of *Streptomyces* sp. EBS2.9 against *Pseudoplagiostroma eucalypti* LS6: A) Control without actinobacteria; B) Strain EBS2.9 shows good inhibition.

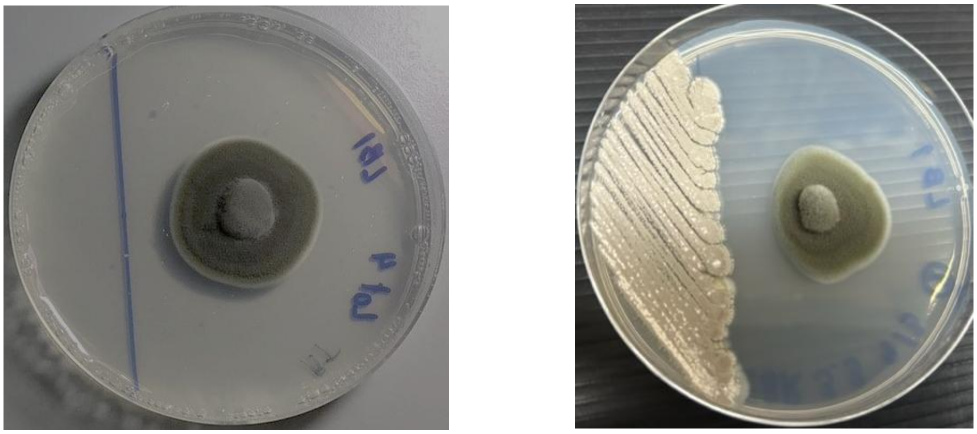

A B

Figure S7. The fungal inhibition of *Streptomyces* sp. EKL7.15 against *Cladosporium* sp. LB1: A) Control without actinobacteria; B) Strain EKL7.15 shows good inhibition.

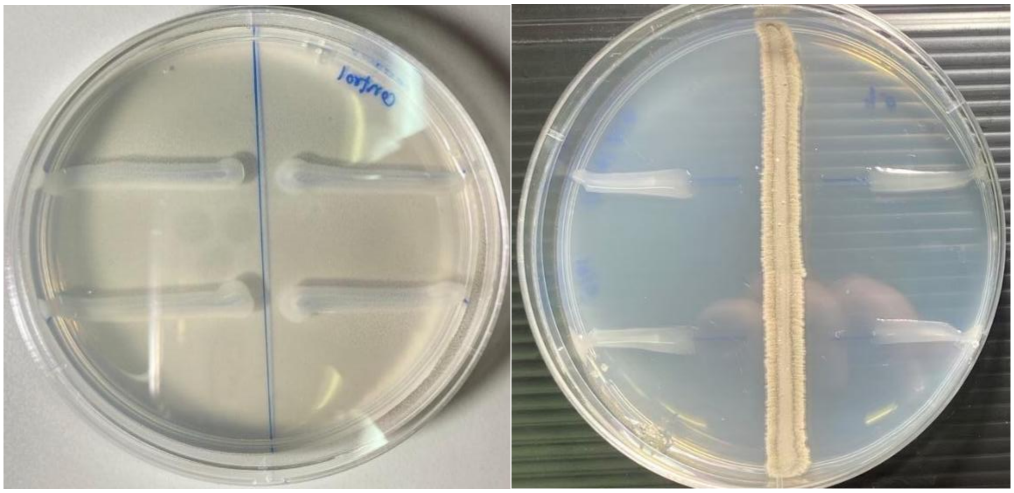

A B

Figure S8. The bacterial inhibition test of *Streptomyces* sp. EWL3.4 against *Ralstonia solanacearum* TISTR 2069: A) Control without actinobacteria; B) Strain EWL3.4 shows moderate inhibition.

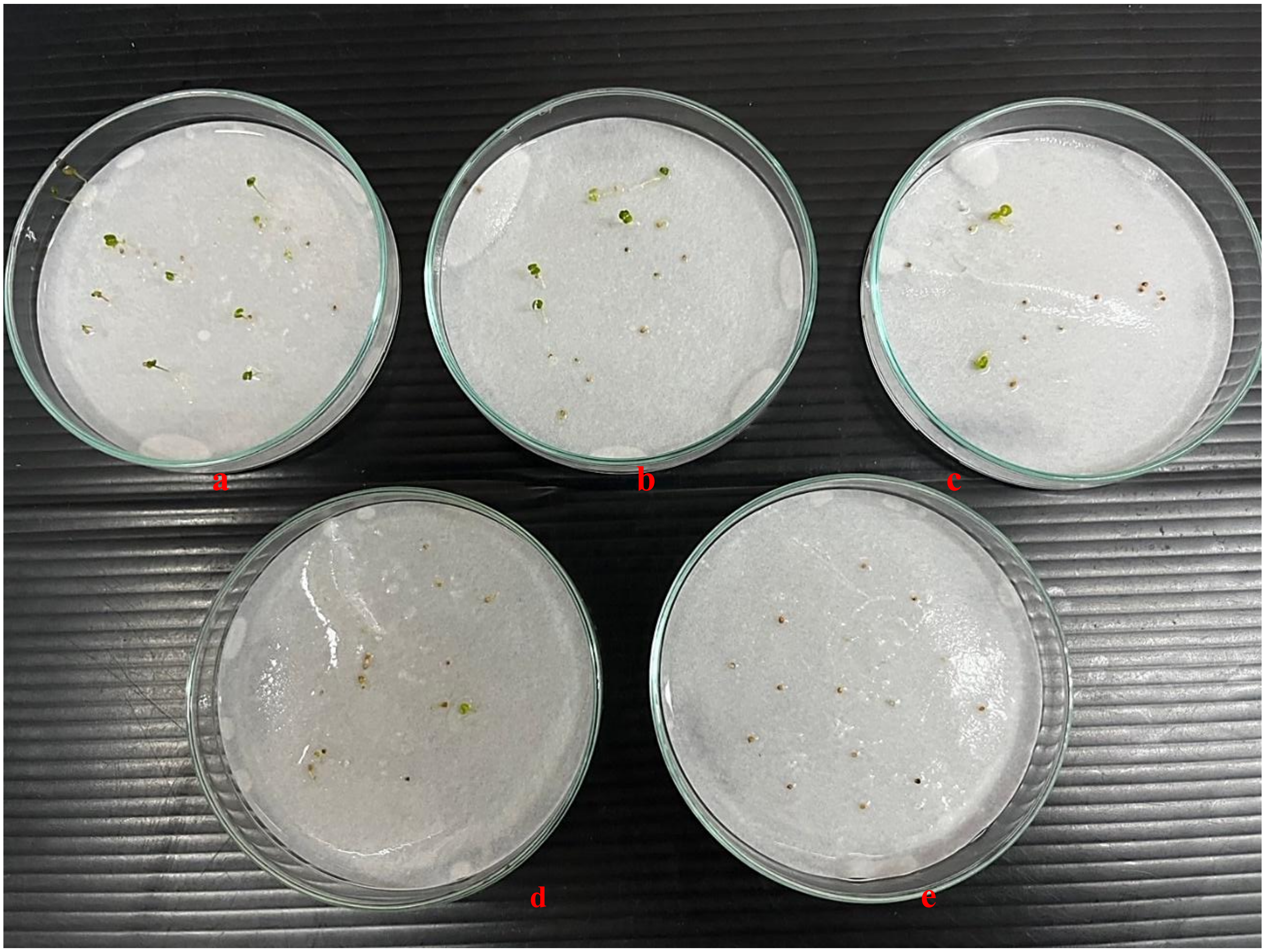

A)

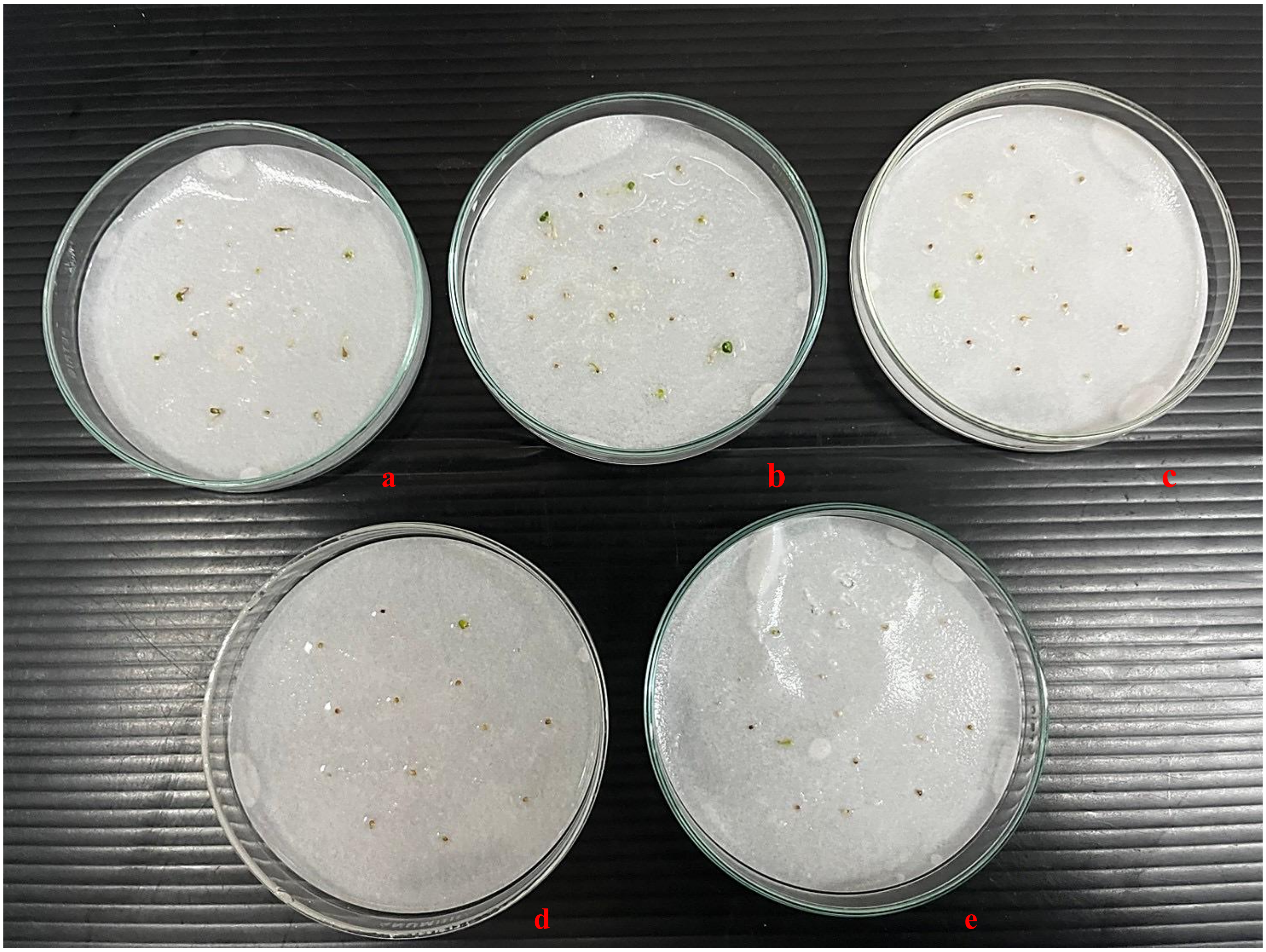

B)

Figure S9. Seed germination test of *Eucalyptus* at 0 (a), 50 (b), 100 (c), 150 (d), and 200 (e) (mM NaCl). A) Seeds were treated with water (control). B) Seeds were treated with *Streptomyces* strain EWL5.1.
